# Restricted visitation policies in acute care settings during the COVID-19 pandemic: a scoping review

**DOI:** 10.1186/s13054-021-03763-7

**Published:** 2021-09-25

**Authors:** Stephana J. Moss, Karla D. Krewulak, Henry T. Stelfox, Sofia B. Ahmed, Melanie C. Anglin, Sean M. Bagshaw, Karen E. A. Burns, Deborah J. Cook, Christopher J. Doig, Alison Fox-Robichaud, Robert Fowler, Laura Hernández, Michelle E. Kho, Maia Kredentser, Kira Makuk, Srinivas Murthy, Daniel J. Niven, Kendiss Olafson, Ken Kuljit S. Parhar, Scott B. Patten, Oleksa G. Rewa, Bram Rochwerg, Bonnie Sept, Andrea Soo, Krista Spence, Sean Spence, Sharon Straus, Andrew West, Jeanna Parsons Leigh, Kirsten M. Fiest

**Affiliations:** 1 Department of Critical Care Medicine, Cumming School of Medicine, University of Calgary, Calgary, AB Canada; 2 Department of Medicine, Cumming School of Medicine, University of Calgary, Calgary, AB Canada; 3 Department of Critical Care Medicine, Faculty of Medicine and Dentistry, University of Alberta, and Alberta Health Services, Edmonton, Canada; 4 Interdepartmental Division of Critical Care, University of Toronto, Toronto, ON Canada; 5 Department of Medicine, McMaster University, Hamilton, ON Canada; 6 Department of Medicine, University of Toronto, Toronto, ON Canada; 7 School of Rehabilitation Science, McMaster University, Hamilton, ON Canada; 8 Department of Clinical Health Psychology, University of Manitoba, Manitoba, Canada; 9 Department of Pediatrics, University of British Columbia, British Columbia, Canada; 10 Department of Medicine, University of Manitoba, Manitoba, Canada; 11 Department of Psychiatry, Cumming School of Medicine, Cuthbertson and Fischer Chair in Pediatric Mental Health, University of Calgary, Calgary, AB Canada; 12 Department of Critical Care Medicine, University of Alberta, Edmonton, AB Canada; 13 Department of Critical Care Medicine, Cumming School of Medicine, University of Calgary and Alberta Health Services, Calgary, AB Canada; 14 Chinook Regional Hospital, Lethbridge County, Alberta, Canada; 15 Canadian Society of Respiratory Therapists, Saint John, NB Canada; 16 School of Health Administration, Faculty of Health, Dalhousie University, Halifax, NS Canada

**Keywords:** COVID-19, Hospital Policy, Acute care, Family presence, Visitors

## Abstract

**Background:**

Restricted visitation policies in acute care settings because of the COVID-19 pandemic have negative consequences. The objective of this scoping review is to identify impacts of restricted visitation policies in acute care settings, and describe perspectives and mitigation approaches among patients, families, and healthcare professionals.

**Methods:**

We searched Medline, Embase, PsycINFO, Healthstar, CINAHL, Cochrane Central Register of Controlled Trials on January 01/2021, unrestricted, for published primary research records reporting any study design. We included secondary (e.g., reviews) and non-research records (e.g., commentaries), and performed manual searches in web-based resources. We excluded records that did not report primary data. Two reviewers independently abstracted data in duplicate.

**Results:**

Of 7810 citations, we included 155 records. Sixty-six records (43%) were primary research; 29 (44%) case reports or case series, and 26 (39%) cohort studies; 21 (14%) were literature reviews and 8 (5%) were expert recommendations; 54 (35%) were commentary, editorial, or opinion pieces. Restricted visitation policies impacted coping and daily function (*n* = 31, 20%) and mental health outcomes (*n* = 29, 19%) of patients, families, and healthcare professionals. Participants described a need for coping and support (*n* = 107, 69%), connection and communication (*n* = 107, 69%), and awareness of state of well-being (*n* = 101, 65%). Eighty-seven approaches to mitigate impact of restricted visitation were identified, targeting families (*n* = 61, 70%), patients (*n* = 51, 59%), and healthcare professionals (*n* = 40, 46%).

**Conclusions:**

Patients, families, and healthcare professionals were impacted by restricted visitation polices in acute care settings during COVID-19. The consequences of this approach on patients and families are understudied and warrant evaluation of approaches to mitigate their impact. Future pandemic policy development should include the perspectives of patients, families, and healthcare professionals.

*Trial registration*: The review was registered on PROSPERO (CRD42020221662) and a protocol peer-reviewed prior to data extraction.

**Supplementary Information:**

The online version contains supplementary material available at 10.1186/s13054-021-03763-7.

## Introduction

Family members of hospitalized patients are increasingly recognized as crucial to delivery of patient- and family-centred care to aid in mobility, offer emotional support to patients, and inform the health care team about the person they are caring for—before and beyond their illness [1]. Family members are not passive bystanders and play an important role in the care of patients [2]. They may be the first to recognize subtle changes in a patient, help provide a locus of familiarity for the patient, assist in improving processes of care associated with transitions in care, and may act as proxies or advocates for patient treatment decisions [3, 4]. Family presence at the bedside can improve communication and build trust among patients, families, and healthcare professionals [5].

Restricted visitation in acute care settings may occur during times of public health crises such as the COVID-19 pandemic [6]. Policies that mandate restricted family member visitation in acute care settings have been commonly enacted throughout the COVID-19 pandemic to help limit spread of the severe acute respiratory syndrome coronavirus 2 (SARS-CoV-2) virus, reduce use of personal protective equipment (PPE), and organize care [7, 8]. These restrictions have been reported to result in consequences for patients and families alike, such as distress [9], grief [10], impaired coping [10], and reduced quality-of-life [11]. Restricted visitation policies have also been reported to impact mental health and well-being of healthcare professionals, including peritraumatic dissociation [12], moral distress (when one feels the ethically correct action is different from what one is doing) [13, 14], burnout (exhaustion from excessive, prolonged stress and general stressors in the work environment), and compassion fatigue (triggered by continual use of empathy and emotional energy) [15].

Despite intentions to ensure patient, family and healthcare professional safety, there is a growing body of evidence suggesting that restricted visitation policies enacted in acute care settings due to the COVID-19 pandemic have had unintended consequences warranting further exploration [16–20]. The purpose of this scoping review is to synthesize the literature reporting on restricted visitation policies in acute care settings enacted because of the COVID-19 pandemic, describe perspectives on and impacts of restricted visitation policies among patients, families, and healthcare professionals, and identify mitigation approaches aimed at improving patient- and family-centred care during periods of restricted visitation.

## Methods

The research question and methods for study selection and data charting were prespecified (PROSPERO ID: CRD42020221662), published (*accepted, BMJ Open)*, and performed as recommended by Arksey and O’Malley [21] and refined using the Scoping Review Methods Manual of the Joanna Briggs Institute [22] and Levac et al. [23]. Investigators adhered to and reported on the PRISMA-ScR Extension for Scoping Reviews [24].

### Review questions

1. What are the impacts of restricted hospital visitation policies due to the COVID-19 pandemic on patients, family members or healthcare professionals of hospitalized patients?
2. What approaches have been taken to mitigate the impact on patients, family members, or healthcare professionals?

### Eligibility criteria

The components of population, exposure, comparator, outcome, study design, and timeframe are as follows:

- *Population* Patients, family members (i.e., relatives, close friends), or healthcare professionals (e.g., nurses, physicians, respiratory therapists) of adult patients (> 17 years of age, or as defined in the individual study) who were hospitalized at acute care facilities (i.e., tertiary academic or community hospitals, or specialized care centers part of a larger hospital) during the COVID-19 pandemic.
- *Exposure* Restricted visitation for hospitalized patients because of the COVID-19 pandemic.
- *Comparator* Any comparator.
- *Outcomes* Perspectives (i.e., views or prospects), experiences (i.e., encounters), or quantitative impacts (listed below) among patients, family members or healthcare professionals, as well as approaches or strategies taken to mitigate impact (e.g., support groups, virtual communication platforms).
- *Study Design* Any observational or interventional primary research study, including focus groups and qualitative inquires, as well as reviews, commentaries, editorials, opinions, case studies and case reports, or reports from expert advisory groups
- *Timeframe* Publications from December 01, 2019 to January 01, 2021.

### Search strategy

We performed systematic searches that were unrestricted by date and language in Medline, Embase, PsycINFO, Healthstar, the Cumulative Index to Nursing and Allied Health Literature (CINAHL) database, and the Cochrane Central Register of Controlled Trials. Experienced librarians developed (N.D.) and PRESS reviewed (D.L.) all searches that were performed on January 01, 2021 using subject headings, keywords, and related synonyms from a combination of the following terms: COVID-19; restricted visitation policies; patients, family members, and healthcare professionals; perspectives, experiences, and impacts. We searched bibliographies of identified records for additional relevant records. The full Medline search strategy is available in an online appendix (Additional file 1: Table S1). Given the evolving nature of the COVID-19 pandemic and the wealth of relevant, primary data reported in unpublished sources, we performed manual searches in web-based resources: Google, Google Scholar, three journals (i.e., *Journal of the American Medical Association, New England Journal of Medicine, The Lancet*), and websites (e.g., United States Center for Disease Control, United States National Health Council, World Health Organization). We also performed manual searches in ProQuest (for dissertations) and medRxiv (the preprint server for health sciences). The terms “COVID-19” and “Hospital Policy” were searched separately, and the first five pages screened with no limitations by study design, language, or year. Sources and dates for grey literature and key journal searches are presented in Additional file 1: Table S2. References were exported and managed using EndNote X9 (Clarivate Analytics).

### Record selection

We selected records that reported perspectives (i.e., mental views or prospects) on or impacts (i.e., any diagnosed psychopathologies or symptoms of psychopathologies, neurocognitive disorders or symptoms of neurocognitive disorders; health-related quality of life, self-efficacy [ability to function and maintain relationships], general well-being [coping, sense of meaning, purpose, optimism and hopefulness]) of restricted visitation policies enacted in acute care settings in response to the COVID-19 pandemic. We included primary (e.g., quantitative, qualitative) and secondary (e.g., reviews, recommendations) research, as well as non-research records (e.g., commentaries, editorials, opinions). We defined restricted visitation policies in acute care settings as restrictions to visitation for hospitalized (i.e., tertiary academic and community hospitals [including specialized care centers]) patients to limit the spread of COVID-19, reduce use of PPE, or help organize care [7, 8].

We included adult patients (> 17 years of age, or as defined in the individual record), their family members (i.e., relatives, close friends) or healthcare professionals (e.g., nurses, physicians, respiratory therapists) caring for adult patients. We excluded records if they described a restricted visitation policy for hospitalized children (as the restricted visitation policies and exceptions to these policies vary according to the patient-family member/provider relationship) or those in a specialized care center or long-term care facility (e.g., senior residences). We included records regardless of patient location (e.g., emergency, ward, ICU) or COVID-19 status.

After a calibration exercise to ensure reliability among reviewers, we used a two-stage unblinded process to select records. In the first stage, reviewers (S.M., M.A., L.H., K.M.) independently in duplicate reviewed titles and abstracts of publications identified through the search strategy. Any record selected for inclusion by any reviewer progressed to the next stage. Following a second calibration exercise prior to the second stage, two reviewers (S.M., M.A., L.H., K.M.) independently in duplicate reviewed all full-texts of remaining records, selecting those that satisfied all inclusion criteria. In this stage, two-reviewer agreement was needed for a record to move forward, with discrepancies being resolved in consultation with a third reviewer (K.K.).

### Data charting

In another calibration exercise to achieve > 75% inter-rater agreement, we trained the same reviewers from record selection (S.M., M.A., L.H., K.M.) to independently abstract data in duplicate using a standardized form. We abstracted the following data: record identifiers and type (e.g., purpose, sample size, measures), participants (i.e., patients, family members, healthcare professionals), exposure (i.e., restricted visitation policy), and outcome (i.e., perspectives, impacts) information, as well as information on approaches or strategies (e.g., education, support groups) taken to mitigate impact, and conclusions and recommendations. We contacted corresponding authors if reported policies were unclear and we subsequently categorized all policies by a classification scheme based on the number of visitors allowed per adult patient in acute care settings (by Valley and colleagues, Additional file 1: Figure S1) [25].

In addition, we assessed approaches or strategies to mitigate impact using a six-step model aimed at addressing our research objectives based on the method described by Kastner et al. [26]: (1) steps or guiding principles to conduct the approach (e.g., elements, or a step-wise protocol); (2) derivation of the approach from empirical evidence (i.e., if derived from observation and experiment, or published theory); (3) minimum expertise to conduct the approach (i.e., whether additional personnel are required [e.g., social worker, psychiatrist]); (4) limitations to the approach (e.g., dependence on sufficient supply of PPE, requirement of stable WiFi connection or personal device); (5) reproducibility of the approach (i.e., operationalized, evidenced by use in multiple sites); (6) feasibility of the approach to other contexts (i.e., generalizable, considering internal validity should precede external validity). Two reviewers (S.M., M.A.) independently in duplicate summed strategies using a scoring system developed by our team (range: 0, lowest; 6, highest) wherein one point was assigned for each of these six domains if mentioned in the record. Following discussion of discrepancies, a second round of summation by the same two reviewers (S.M., M.A.) was taken to reach 100% agreement.

### Collating, summarizing, and reporting results

Two reviewers (S.M., M.A., L.H., K.M.) independently and in duplicate analyzed data in a two-stage process according to validated guidelines for narrative synthesis of quantitative studies [24] and thematic synthesis of qualitative studies for reviews on health research [27]. Qualitative thematic analysis was performed to compare and contrast findings across studies as we had to include literature of variable types to conduct a rich, comprehensive review due to lack of published, peer-reviewed research evaluating the effects of visitation restrictions. We evaluated all articles in both stages by identifying key outcomes and themes described. Impacts reported (or hypothesized for non-primary research records) to be associated with restricted visitation policies were grouped by theme and classified as patient-, family-, or provider-related. Considering heterogeneity of the quantitative data, we grouped studies according to outcomes measured and summarized data as counts with proportions using STATA 16.1 (StataCorp, TX). We evaluated contextual (e.g., setting, population) and methodological (e.g., tools, timing) factors to explain variability in quantitative outcomes. We evaluated qualitative studies (that reported on perspectives) by inductive line-by-line coding, followed by development of descriptive themes representing themes reported in the record, and generation of analytical themes to develop new interpretive constructs, explanations, or hypotheses. The qualitative analysis focused on identifying key concepts that overlapped between records to refine our findings into core themes for future research.

## Results

**Fig. 1:**
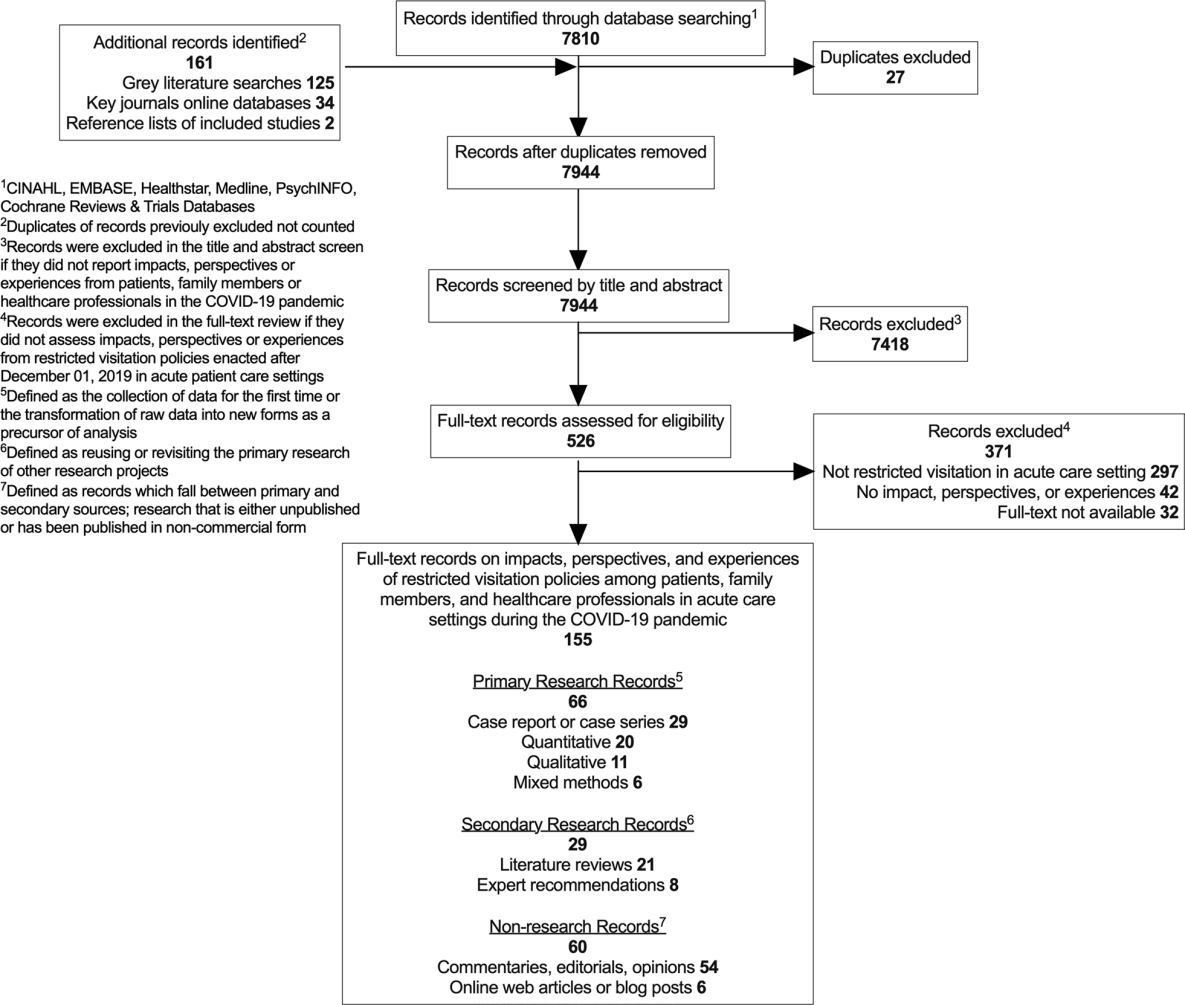
Record selection flow chart

We identified 7810 unique records, of which 526 full texts were reviewed and 155 eligible records included. Records were written in five languages (142 [92%] English; 4 [3%] French; 4 [3%] Korean; 3 [2%] Italian; 2 [1%] Spanish) and translated to English for review (Fig. 1). Records that reported qualitative findings that required translation (3/4 [75%] French; 1/3 [33%] Italian; 2/2 [100%] Spanish) were translated by fluent individuals. Records that reported quantitative findings only that required translation (1/4 French [25%]; 4/4 [100%] Korean; 2/3 [66%] Italian) were translated online (Yandex.translate). Most (*n* = 297/371, 80%) records were excluded because they did not report a restricted visitation policy in an acute care setting.

### Description of records

Of the 155 records included in this review (Additional file 1: Table S3), most were primary research (*n* = 66, 43%) including case reports or case series (*n* = 29, 44%) and cohort studies (*n* = 26, 39%). Secondary records (*n* = 29, 19%) included literature reviews (*n* = 21, 72% [*n* = 1 systematic review, 5%; *n* = 20 narrative reviews, 95%) and expert recommendations (*n* = 8, 28%). Among the 60 (39%) unpublished, non-research records retrieved, 90% (*n* = 54) were commentary, editorial, or opinion pieces and 10% (*n* = 6) were online web articles or blog posts; all non-research records (100%) reported primary, relevant data. Additional file 1: Figure S2 illustrates the countries and acute care settings, and Additional file 1: Figure S3 presents the characteristics of restricted visitation policies among the included records. Records primarily originated from the United States (*n* = 72, 46%), United Kingdom (*n* = 13, 8%), or Italy (*n* = 11, 7%), reported on restricted visitation policies enacted hospital-wide (*n* = 38, 25%) or in palliative care settings (*n* = 36, 23%), and were published or posted January through December 2020. Of the 30 (19%) records reporting on the impacts of restricted visitation policies in hospital, most focused on the impacts on healthcare professionals (*n* = 14, 47%) with few records assessing the impacts on patients (*n* = 8, 27%) and families (*n* = 7, 23%). One hundred and forty records (90%) described perspectives on restricted visitation policies. These were predominantly perspectives among healthcare professionals (*n* = 74, 53%), followed by patients (*n* = 13, 9%), and families (*n* = 8, 6%). Outcomes (impacts and perspectives) are summarized below and in Additional file 1: Table S4.

### The impact of restricted visitation

**Fig. 2:**
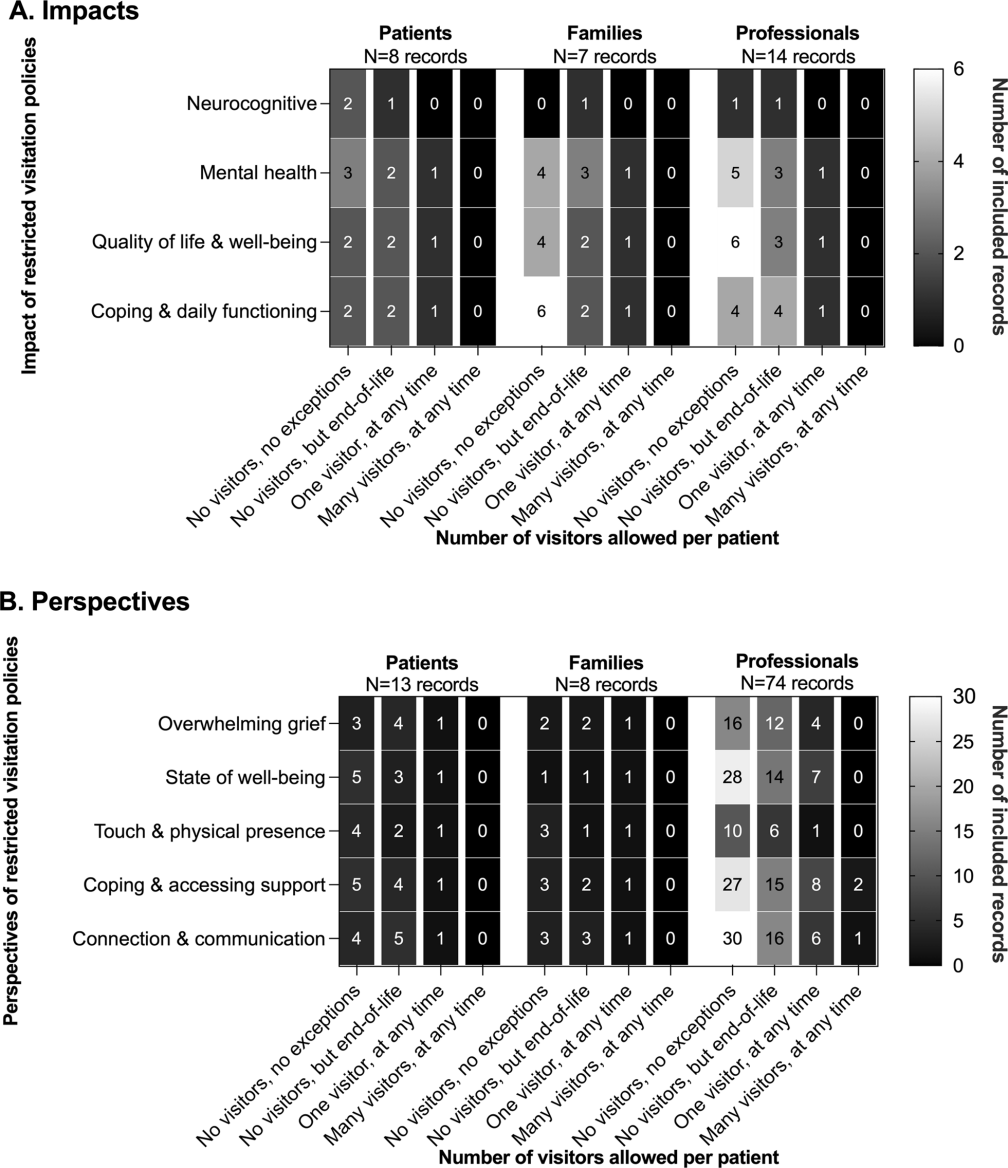
Total number of reported **A** impacts^1^ and **B** perspectives^2^ of restricted visitation policies in acute care settings during the COVID-19 pandemic on patients, family members, and healthcare professionals, by number of visitors allower per patient*. ^1^Eighteen unique included records specified restricted visitation policies and assessed impact on participants quantitatively. ^2^Seventy-eight unique included records specified restricted visitation policies and reported perspectives from participants

Thirty (19% of 155) records reported impacts of restricted visitation policies that were assessed by measuring neurocognitive (e.g., delirium) (*n* = 6/30, 20%) and mental health outcomes (e.g., depression) (*n* = 23/30, 77%), quality of life and well-being (e.g., life satisfaction) (*n* = 22/30, 73%), and coping and daily function (e.g., disabilities, access to support) (*n* = 23/30, 77%) (Table 1). Eighteen records (*n* = 18/30, 60%) specified restricted visitation policies that included mandating no visitors without exceptions (*n* = 13/30, 43%), no visitors except during end-of-life care (*n* = 4/30, 13%), or one visitor at any time (*n* = 1/30, 3%). Twelve records (*n* = 12/30, 40%) did not specify the restrictions on visitation. Figure 2a depicts impacts of restricted visitation policies by type of policy restriction from all included records that reflect published literature at that point in time. The impact of no visitations without exception differed by participant group. These included mental health outcomes for patients (*n* = 3/13, 23%), coping and daily functioning for families (*n* = 6/13, 46%), and quality of life and well-being for healthcare professionals (*n* = 6/13, 46%). Impacts on patients, families, and professionals from restricted visitation policies among primary research records are synthesized in Table 2.

**Table 1:** Measured outcomes on impacts of restricted visitation policies during the COVID-19 pandemic from patients, family members, and healthcare professionals

Impacts^a^	Patients	Family members	Healthcare professionals
	*N* = 16	*N* = 11	*N* = 18
**Neurocognitive**	**3 (18.8%)**	**1 (9.1%)**	**1 (5.6%)**
Delirium	3 (100%)^b^	0 (0.0%)	0 (0.0%)
Euphoria	0 (0.0%)	0 (0.0%)	1 (100%)^b^
Exaltation	0 (0.0%)	0 (0.0%)	1 (100%)^b^
Insomnia	0 (0.0%)	1 (100%)^b^	1 (100%)^b^
**Mental health**	**12 (75%)**	**8 (72.7%)**	**9 (50.0%)**
Anxiety	4 (33.3%)^b^	3 (37.5%)	5 (55.6%)^b^
Burnout	0 (0.0%)	0 (0.0%)	5 (55.6%)^b^
Depression	1 (8.3%)	0 (0.0%)	3 (33.3%)
Distress	3 (25.0%)	4 (50.0%)^b^	1 (11.1%)
Grief	2 (16.7%)	4 (50.0%)^b^	1 (11.1%)
Mental health disorders	2 (16.7%)	0 (0.0%)	1 (11.1%)
Peritraumatic stress disorder	0 (0.0%)	0 (0.0%)	1 (11.1%)
Posttraumatic stress disorder	2 (16.7%)	0 (0.0%)	1 (11.1%)
Psychosocial isolation	1 (8.3%)	0 (0.0%)	0 (0.0%)
**Quality of life and well-being**	**8 (50%)**	**8 (72.7%)**	**10 (55.6%)**
Ethical climate	0 (0.0%)	0 (0.0%)	1 (10.0%)
Fear, physical	1 (12.5%)	1 (12.5%)	2 (10.0%)
Quality of life	2 (25.0%)	3 (35.6%)^b^	4 (40.0%)^b^
Recovery	3 (37.5%)^b^	0 (0.0%)	0 (0.0%)
Satisfaction	2 (25.0%)	2 (25.0%)	1 (10.0%)
Well-being	2 (25.0%)	2 (25.0%)	2 (20.0%)
**Coping and daily functioning**	**9 (56.3%)**	**10 (90.9%)**	**12 (66.7%)**
Access to support	5 (55.6%)	7 (70.0%)^b^	5 (41.7%)^b^
Disabilities, hearing and sight	0 (0.0%)	2 (20.0%)	0 (0.0%)
Education and guidelines	0 (0.0%)	0 (0.0%)	3 (25.0%)
Leadership	0 (0.0%)	0 (0.0%)	4 (33.3%)
Palliative and spiritual care	4 (44.4%)	2 (20.0%)	0 (0.0%)
Post-discharge care	4 (44.4%)	3 (30.0%)	0 (0.0%)

Percentages do not add up to 100 due to the possibility of multiple outcomes per record

^a^Categories determined from data charting of included records

^b^Impacts that were predominantly reported

**Table 2:** Number of reported impacts^a^ on and perspectives^b^ from restricted visitation policies in acute care settings during the COVID-19 pandemic on patients, family members, and healthcare professionals among primary research records^c^ included in the review, by the number of visitors allowed per patient

Population	Impacts *N* = 17 (30%)				Perspectives *N* = 39 (70%)				
	Neurocognitive	Mental health	Quality of life and well-being	Coping and daily functioning	Overwhelming grief	State of well-being	Touch and physical presence	Coping and accessing support	Connection and communication
	*N* = 2 (12%)	*N* = 16 (94%)	*N* = 15 (88%)	*N* = 17 (100%)	*N* = 16 (41%)	*N* = 21 (54%)	*N* = 8 (21%)	*N* = 28 (71%)	*N* = 25 (64%)
Patients *N* = 13 (23%)	2 (100%)	6 (38%)	4 (27%)	5 (29%)	6 (38%)	6 (29%)	5 (63%)	9 (32%)	8 (32%)
Family members *N* = 9 (16%)	0 (0%)	6 (38%)	8 (53%)	8 (47%)	4 (25%)	2 (10%)	4 (50%)	5 (18%)	6 (24%)
Professionals *N* = 35 (63%)	0 (0%)	4 (25%)	3 (20%)	5 (29%)	14 (88%)	17 (81%)	5 (63%)	23 (82%)	22 (88%)

^a^Seventeen primary research records specified restricted visitation policies and assessed impact on participants quantitatively

^b^Thity-nine primary research records specified restricted visitation policies and reported perspectives from participants qualitatively

^c^Sixty-six primary research records were included in the scoping review; fifty-six of which specified restricted visitation policies

^d^Impacts or perspectives reported from ≥ 50% of records for patients, families, or healthcare professionals

### Perspectives on restricted visitation

Table 3 summarizes perspectives from patients, families, and healthcare professionals on restricted visitation policies in acute care settings during the COVID-19 pandemic from 140 included records (90% of 155). Table 2 also summarizes perspectives as reported in primary research records. The most common perspectives were related to connection and communication (e.g., consistency and clarity) (*n* = 70/140, 50%), coping and accessing support (e.g., availability and processes) (*n* = 68/140, 49%), and state of well-being (e.g., burnout) (*n* = 61/140, 44%). Relatively few records reported perspectives related to overwhelming grief (e.g., complicated grief) (*n* = 45/140, 32%) and loss of touch and physical connection (*n* = 29/140, 21%). Of the 81 (57%) records that specified enacted policies, 46 (57%) mandated no visitors without exceptions. Common perspectives from patients were state of well-being (*n* = 5/46, 11%) and coping and accessing support (*n* = 5/46, 11%). Figure 2b illustrates perspectives on restricted visitation policies by type of policy restriction. Perspectives from family members that experienced no visitor restrictions focused on touch and physical presence (*n* = 3/46, 7%), coping and accessing support (*n* = 3/46, 7%), and connection and communication [with isolated patients] (*n* = 3/46, 7%). Healthcare professionals mainly commented on intentional practices to facilitate [virtual] connection and communication [for isolated patients] to sustain personalized patient care (*n* = 30/46, 65%).

**Table 3:** Themes and subthemes on perspectives on restricted visitation policies during the COVID-19 pandemic from patients, family members, and healthcare professionals

Perspectives^a^	Patients	Family members	Healthcare professionals
	*N* = 26	*N* = 13	*N* = 139
**Overwhelming grief**	**8 (30.8%)**	**5 (38.5%)**	**62 (44.6%)**
Absent	1 (12.5%)	1 (20.0%)	7 (11.3%)
Anticipatory	0 (0.0%)	0 (0.0%)	3 (4.8%)
Common	1 (12.5%)	1 (20.0%)	5 (8.1%)
Complicated	3 (37.5%)	3 (60.0%)^b^	34 (54.8%)^b^
Delayed	3 (37.5%)	1 (20.0%)	5 (8.1%)
Disenfranchised	4 (50.0%)^b^	3 (60.0%)^b^	16 (25.8%)
Exaggerated	0 (0.0%)	0 (0.0%)	8 (12.9%)
Inhibited	2 (25.0%)	0 (0.0%)	6 (9.7%)
Process of grieving	4 (50.0%)^b^	1 (20.0%)	12 (19.4%)
**State of well-being**	**13 (50.0%)**	**5 (38.5%)**	**83 (59.7%)**
Anxiety	0 (0.0%)	0 (0.0%)	9 (10.8%)
Burden	2 (15.4%)	1 (20.0%)	19 (22.9%)
Burnout	0 (0.0%)	0 (0.0%)	6 (7.2%)
Depression	1 (7.7%)	1 (20.0%)	6 (7.2%)
Distress	6 (46.2%)^b^	5 (100.0%)^b^	23 (27.7%)^b^
Emotional well-being	2 (15.4%)	1 (20.0%)	15 (18.1%)
Mental health disorders	2 (15.4%)	0 (0.0%)	17 (20.5%)
Posttraumatic stress disorder	0 (0.0%)	0 (0.0%)	3 (3.6%)
Professional support	4 (30.8%)	2 (40.0%)^b^	23 (27.7%)^b^
Psychosocial isolation	1 (7.7%)	0 (0.0%)	9 (10.8%)
Stress	0 (0.0%)	1 (20.0%)	5 (6.0%)
Trauma	0 (0.0%)	0 (0.0%)	5 (6.0%)
**Touch and physical presence**	**7 (26.9%)**	**5 (38.5%)**	**29 (20.9%)**
Eye contact	0 (0.0%)	0 (0.0%)	4 (13.8%)
Humanization	1 (14.3%)	2 (40.0%)	10 (34.5%)
Personal protective equipment	3 (42.9%)	3 (60.0%)	19 (65.5%)^b^
Physical presence	4 (57.1%)^b^	5 (100%)^b^	15 (51.7%)
Physical touch	2 (28.6%)	1 (20.0%)	8 (27.6%)
**Coping and accessing support**	**12 (46.2%)**	**7 (53.9%)**	**88 (63.3%)**
Abandonment and inattention	4 (33.3%)	3 (42.9%)	12 (13.6%)
Availability for support	5 (41.7%)^b^	2 (28.6%)	32 (36.3%)
Education and training	0 (0.0%)	0 (0.0%)	8 (9.1%)
Emotion-focused	1 (8.3%)	1 (14.3%)	10 (11.4%)
Problem-focused	3 (25.0%)	2 (28.6%)	17 (19.3%)
Process of coping	2 (16.7%)	2 (28.6%)	38 (43.2%)^b^
**Connection and communication**	**13 (50.0%)**	**8 (61.5%)**	**86 (61.9%)**
Access and limitations	1 (7.7%)	0 (0.0%)	36 (41.9%)^b^
Compassion delivery	3 (23.1%)	1 (12.5%)	28 (32.6%)
Consistency and clarity	6 (46.2%)^b^	6 (75.0%)^b^	33 (38.4%)
e-Health/telehealth	6 (46.2%)^b^	6 (75.0%)^b^	36 (41.9%)^b^
Education and training	2 (15.4%)	1 (12.5%)	15 (17.4%)
Non-verbal cues	1 (7.7%)	0 (0.0%)	4 (4.7%)

Percentages do not add up to 100 due to the possibility of multiple outcomes per record

^a^Perspectives determined from inductive thematic analysis of included records

^b^Perspectives that were predominantly reported

### Approaches to mitigate the impact of restricted visitation

Additional file 1: Table S5 summarizes 87 approaches (from 87 unique records, 56% of 155 records included) to mitigate the impact of restricted visitation policies. Details on approaches to mitigate the impact by categories considered in our summation procedure are provided in Additional file 1: Table S6. Approaches were targeted to mitigate impact among families (*n* = 61/87, 70%), patients (*n* = 51/87, 59%), or healthcare professionals (*n* = 40/87, 46%). Most records that reported approaches to mitigate impact provided steps or guiding principles for professionals (*n* = 37/40, 93%), families, (*n* = 51/61, 84%), or patients (*n* = 41/51, 80%). Authors of many approaches to mitigate impact of restricted visitation policies reported that approaches were derived, at least in part, from peer-reviewed empirical evidence (i.e., primary research) (patients, *n* = 29/51, 57%; families, *n* = 37/61, 61%; professionals, *n* = 26/40, 65%). Similarly, approaches to mitigate impact were used in multiple sites (patients, *n* = 26/51, 51%; families, *n* = 37/61, 61%; professionals, *n* = 23/40, 56%) and could be applied to other stakeholders (patients, *n* = 26/51, 51%; families, *n* = 36/61, 59%; professionals, *n* = 23/40, 56%).

Using our summation procedure, 14 records (*n* = 14/87, 16%) reported approaches to mitigate the impact of restricted visitation policies that addressed at least five out of six criteria targeting patients (*n* = 7, 50%), families (*n* = 12, 86%), and healthcare professionals (*n* = 10, 71%). These (*n* = 14) approaches used seven main strategies, including: (1) telehealth and videoconferencing platforms [for family members to visit isolated patients] (*n* = 3/14, 21%); (2) providing palliative care [at end-of-life] (*n* = 3/14, 21%); (3) communicating difficult news [over telephone] (*n* = 2/14, 14%); (4) plans to preserve continuity of care (*n* = 3/14, 21%) and (5) a triage system [for limited personnel and resources] (*n* = 1/14, 7%); (6) a mental health hotline for professionals (*n* = 1/14, 7%); and (7) and psychological interventions [to protect against post-traumatic stress and complicated grief] (*n* = 1/14, 7%) for professionals and families of isolate patients.

## Discussion

We have synthesized the evidence examining the impacts of and perspectives on restricted visitation policies in acute care settings enacted in response to the COVID-19 pandemic among patients, their families, and healthcare professionals. Across stakeholders, frequently reported impacts occurred among mental health outcomes, quality of life and well-being, and coping and daily function. Common perspectives were related to states of well-being, connection and communication, and coping and accessing support. We identified many approaches to mitigate the impact of restricted visitation policies. Fourteen comprehensive approaches focused on using telehealth and videoconferencing platforms to enable family visitation, providing palliative care, communicating difficult news by telephone, establishing contingency plans and a triage system for limited personnel and resources, and providing mental health hotlines/psychological interventions for healthcare professionals and families.

Restricted visitation policies in the COVID-19 pandemic are likely to have significant short- and long-term consequences on patients, families, and healthcare professionals, and more broadly the healthcare system, but studies carefully demonstrating these potential effects are limited. Restricting visitation is necessary from a public health perspective but can have unintended but deleterious consequences [28] as patients and their families are in a state of heightened psychological distress owing to the lethality of the SARS-CoV-2 virus [29]. In line with existing literature, we identified that patients are isolated and distressed and restricted families are having to navigate the shared decision-making process differently [30, 31]. The COVID-19 pandemic has also placed a heavy toll on healthcare professionals, who grappled with fatigue while navigating unfamiliar virtual modalities (to connect competent patients with their loved ones), cared for their own colleagues who fell ill, and comforted dying, isolated patients [32]. However, caring for patients at the end of life, clinicians also expressed their humanity, tried to ensure dignity-conserving care, adopt new roles, and catalyze connections [33]. More data on the impact of the COVID-19 pandemic on patients and families is needed to inform healthcare professionals how to better care for patients, with attention to promoting patient- and family-centred care and mitigate against potential health inequities [32, 34].

Existing evidence, though limited, does not support that restricted visitation in acute care settings reduces hospital-related transmission of COVID-19. Healthcare professionals may account for 10–20% of nosocomial COVID-19 diagnoses as they are exposed to the SARS-CoV-2 virus and at high risk of infection and thus contributing to further spread [35]. A systematic review of nosocomial infection reported that only 2% were from a source other than a healthcare professional [36]. Patients admitted to hospital without COVID-19 were also unlikely to acquire infection in hospital, with a recent study from an academic health centre in the United States “within a region of moderate community coronavirus disease” reporting an incidence of 12/11482 [37]. In one study, only 1/697 patients positive for COVID-19 were thought to have acquired the infection in hospital from an asymptomatic visitor [38]. Evidence that visitors to acute care settings are a source of nosocomial transmission or at higher risk of becoming infected is lacking. Stakeholders in policy development should carefully weigh the potential harms of restricted visitation policies against the risk of viral transmission when considering and revising these policies during the COVID-19 pandemic [39].

Restricted visitation policies in acute care settings are not unique to the COVID-19 pandemic. Restricted visitation policies enacted during seasonal influenza (to prevent outbreaks of respiratory viruses) [40] were relaxed given evidence that presence of a family member (or surrogate decision-maker) had beneficial effects for patients (e.g., reduction in delirium and anxiety), their support persons (e.g., satisfaction with care), and healthcare professionals [2, 41]. A pre-(COVID-19)-pandemic systematic review found that accommodating, as opposed to restrictive, ICU visitation policies did not increase acquired infections or septic complications [41]. A subsequent analysis found that more liberal visitation policies had been adopted by 73% of hospitals, compared with only 32% of hospitals in 2015 [42]. Extensive evidence shows that family members are critical to delivery of patient-centred care by, for examples, participating in rounds, advocating for the patient, overcoming language barriers, and assisting with transitions to critical care [43–46]. As healthcare systems adapt restricted visitation policies to the ongoing COVID-19 pandemic, they may unintentionally impact patient engagement with families in provision of care including shared decision-making with the healthcare team [34].

An important strength to our review is that we searched multiple databases without restrictions and several sources of unpublished literature to report major impacts and perspectives on restricted visitation policies in acute care settings, along with proposed approaches to improve ensuing impact. Our review also has limitations. First, during the COVID-19 pandemic, there has been dissemination of results pre-publication, and it is possible despite exhaustive attempts, that some reports could be missed. The results of the literature searches were greater than anticipated. We worked closely with a health research librarian (i.e., information specialist) to ensure that timely completion of the review was feasible. The rapidly evolving COVID-19 evidence base means that study findings may change with time. Second, nearly half of the included studies reported the impacts of the pandemic in the United States. Policy making processes and allocation of resources may vary across healthcare systems and jurisdictions. Consequently, our results may not be generalizable to other regions. Third, some impacts on patients, families, and healthcare professionals may manifest in a delayed fashion and published reports may have inadequate follow-up time to identify late consequences (e.g., mental health problems, workplace attrition) of these policies. Fourth, we could not report on local customization, adaptation, or specific exceptions to restricted visitation policies. A limitation of the published work to date identified in our review is the absence of research formally evaluating the effects of visitation restrictions. Such work is important to inform ongoing management of COVID-19, and policy development for future pandemics, or situations such as multiple casualty incidents. Fifth, while we contacted several authors for information on policies that were enacted but it was not possible to extract accurate data on restricted visitation policies from all records or granular detail (e.g., assessment tools used) for each outcome. Finally, our review focused on reporting the restricted visitation policies in acute care settings and approaches to mitigate their impact that could be adapted or adopted through quality improvement initiatives and tested in future research, contextualized in light of issues such as status of the pandemic or vaccination prevalence.

## Conclusions

The COVID-19 pandemic has resulted in restrictions that have been implemented to reduce potential nosocomial infection or spread but without clear evidence of benefit and with potential adverse consequences. In particular, the negative consequences on hospitalized patients and their families are understudied or unknown. Further evaluation of the impact of restricted visitation and potential efforts at mitigating negative effects are important for ongoing pandemic planning and for other events that may be associated with strain on healthcare systems such as mass casualty incidents.

## Supplementary Information

**Additional file 1**. Supplementary material to “Restricted visitation policies in acute care settings during the COVID-19 pandemic: a scoping review”.

## Data Availability

All data generated or analysed during this review are included in the published records.
