# Supplementary material for: Restricted visitation policies in acute care settings during the COVID-19 pandemic: a scoping review
Source: Crit Care. 2021 Sep 25;25:347. doi: 10.1186/s13054-021-03763-7 (PMC8465762; doi:10.1186/s13054-021-03763-7)
Supplement: Supplementary file 1 — Additional file 1. Supplementary material to “Restricted visitation policies in acute care settings during the COVID-19 pandemic: a scoping review”. [file 13054_2021_3763_MOESM1_ESM.docx]

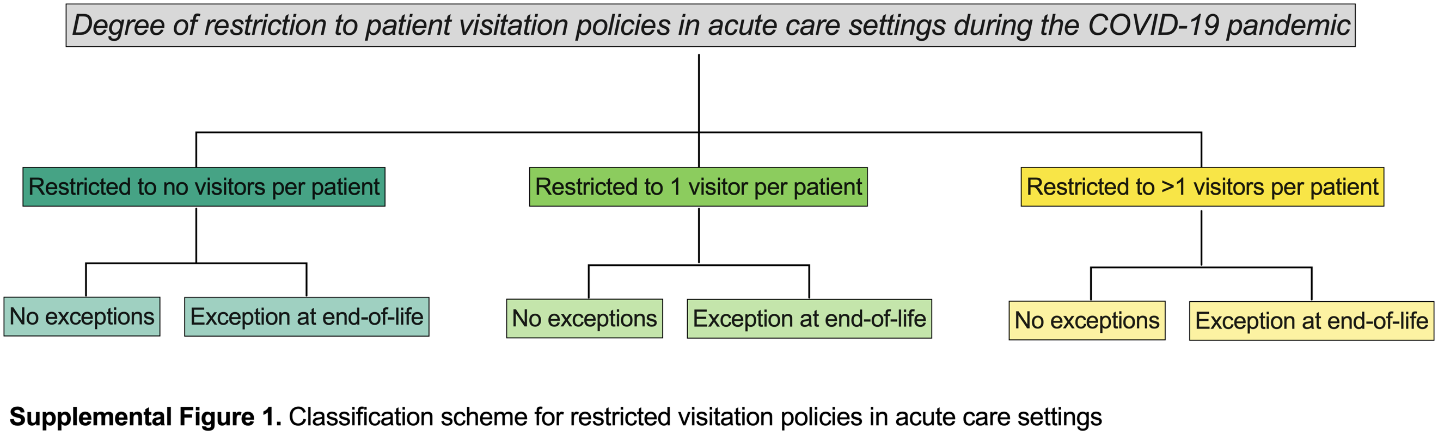


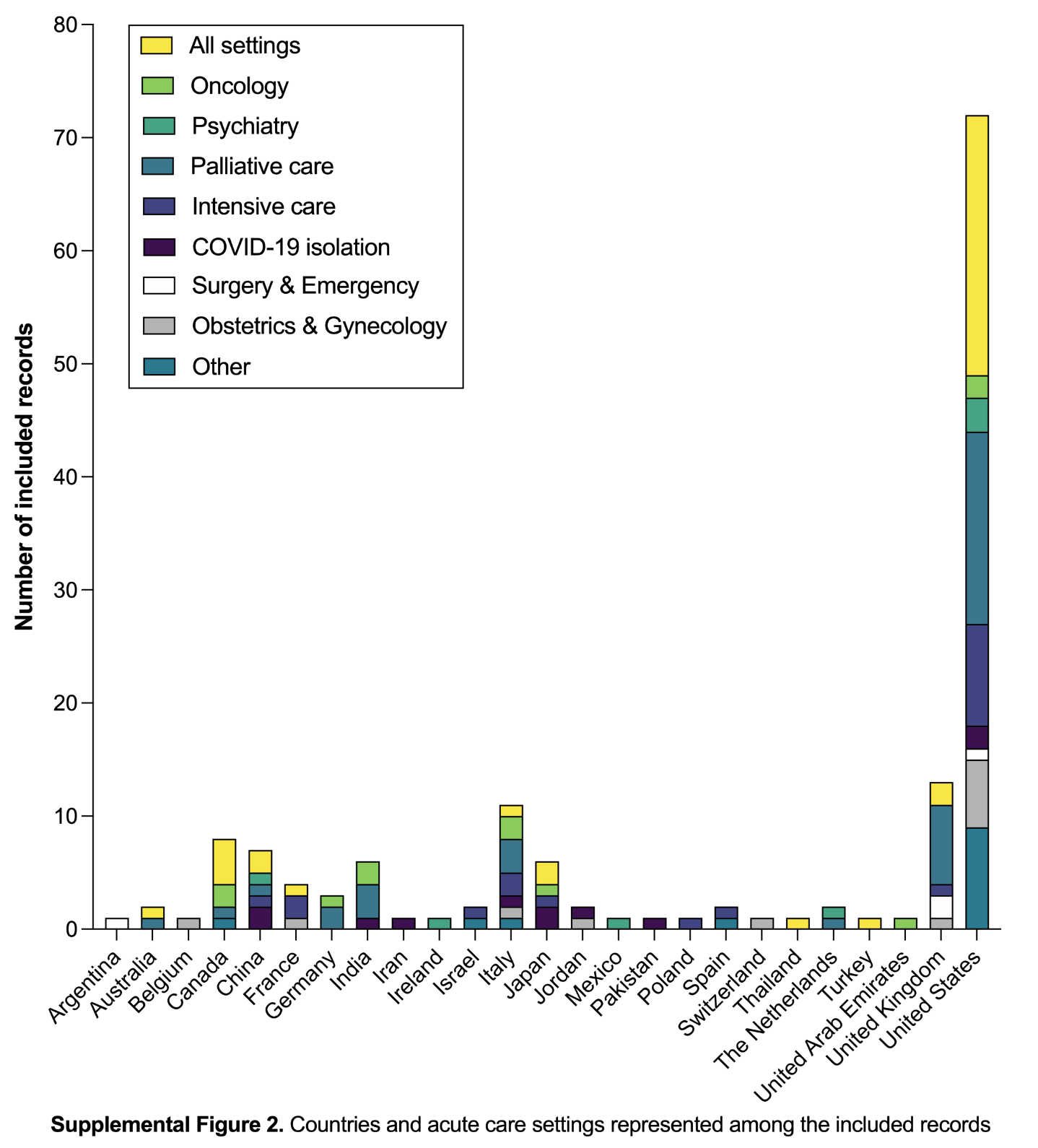

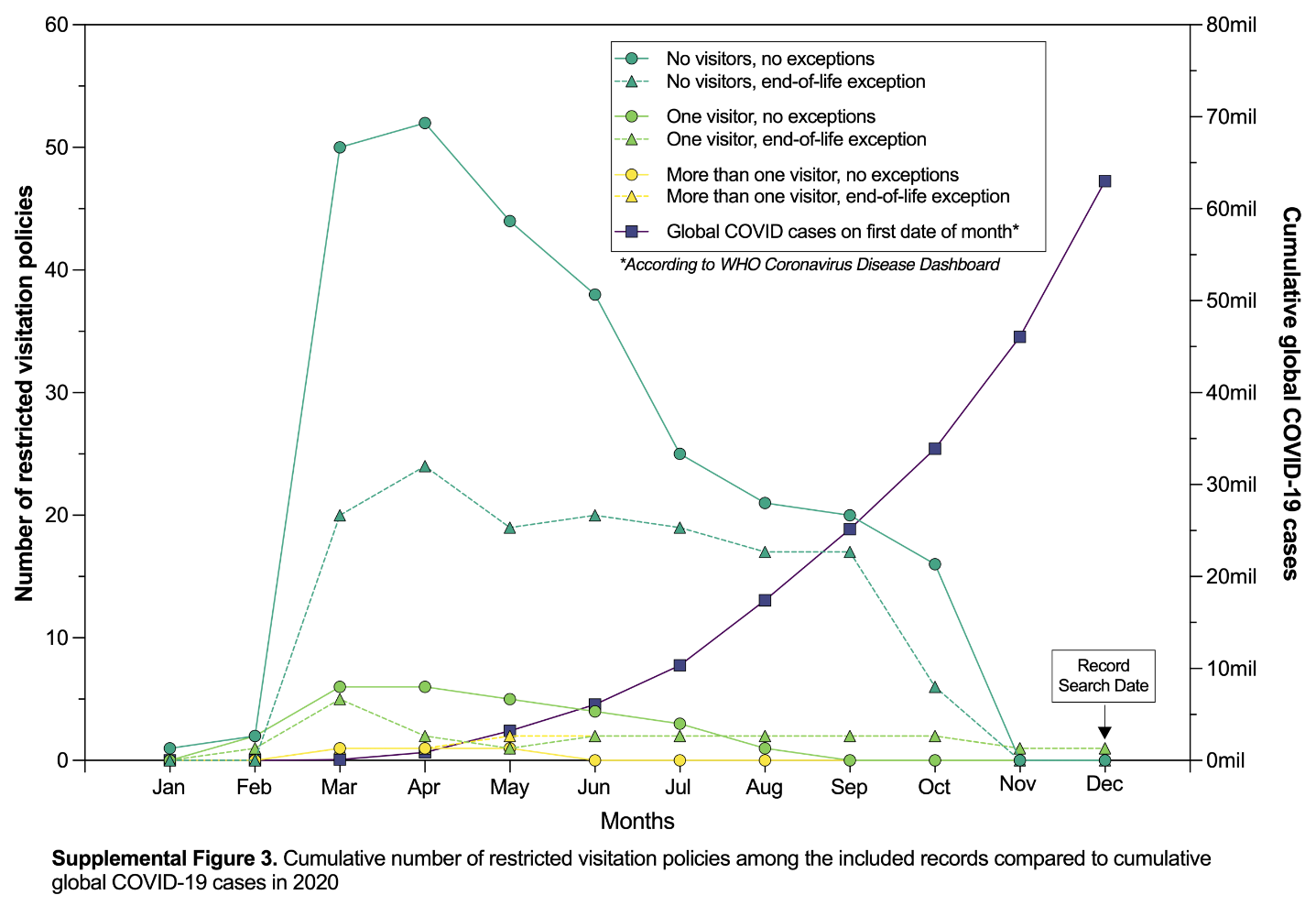


*Number of restricted visitation policies reflect published literature at that point in time.*

**Table S1.** Search strategy used in Medline

| **Search component** | **Search terms** |
| --- | --- |
| COVID-19 pandemic | 1. exp Disease Outbreaks/ 2. exp Coronaviridae Infections/ 3. exp Coronavirus/ 4. (2019-ncov* or 2019ncov* or coronaviru* or corona viru* or covid or covid-19 or covid19* or ncov* or novel cov* or covid-2019 or covid2019 or SARS-COV-2* or SARS-COV2* or SARSCOV-2* or SARSCOV2* or SARSCOV19 or SARS-COV-19 or SARSCOV-19 or SARS-COV-2019 or SARSCOV2019 or SARSCOV-2019 or Wuhan pneumonia or Wuhan virus or severe acute respiratory syndrome coranaviru* or severe acute respiratory syndrome cov 2 or severe acute respiratory syndrome or severe acute respiratory disease).tw,kf. 5. or/1-4 |
| Restricted visitation policies | 1. Visitors to patients/ 2. Restrictions/ 3. Policies/ 4. Materials Management, Hospital/ 5. Health Care Rationing/ 6. Eligibility Determination/ 7. Guideline/ or Practice Guideline/ 8. Decision Making/ or Decision Making, Organizational/ 9. Disaster Planning/ 10. Health Planning/ or Health Plan Implementation/ or Health Priorities/ or Health Resources/ or Health Planning Guidelines/ 11. Clinical Protocols/ 12. Medical Ethics/ or Clinical Ethics/ 13. Patient Admission/ 14. ((health adj2 ration*) or (patient* adj2 (manage* or admi*)) or ((disaster or health) adj2 plan) or visit* or restrict* or decide* or decision* or allocat* or prioritiz* or prioritis* or ethic* or protocol* or guideline* or polic* or criteria).mp 15. or/6-19 |
| Patients, family members, and healthcare professionals | 1. Exp Caregivers/ 2. Exp Family/ 3. Exp Patient/ 4. Exp Patient Care/ 5. Exp Hospitalization/ 6. Family.mp 7. Families.mp 8. Caregiver*.mp 9. “caregiv*”.mp 10. Carer*.mp 11. (spouse or husband* or wife or wives).mp 12. “Next of Kin”.mp 13. Support person.mp 14. “loved one*”.mp 15. “Significant other*”.mp 16. Partner*.mp 17. Relative*.mp 18. (Proxy or proxies or surrogate* or friend or friends).mp 19. Surrogate.mp 20. Friend.mp 21. Exp Patient Care Management/ 22. Exp Delivery of Health Care/ 23. Exp Patient Care Team/ 24. Healthcare Work*.mp 25. Healthcare Provider*.mp 26. Healthcare Practitioner*.mp 27. (nurse* or doctor* or physician or clinician).mp 28. or/21-47 |
| Perspectives, experiences, and impacts | 1. Mental health/ 2. Mental disorder/ 3. Psychiat*.mp 4. ((mental* or psychiatric or psychologic* or behavior* or behaviour* or mood* or panic* or affective or emotion* or neurotic or psychoneurotic or psycho-neurotic or adjustment or reactive) adj2 (health* or ill* or well* or disease* or disorder* or syndrome* or hygiene or symptom* or disturbance* or dysfunction* or attack* or trauma* or condition* or state* or status*)).tw,kf. 5. or/49-52 6. Depression/ 7. Depressive Disorder/ 8. Depressive disorder*.mp. 9. Depress*.mp 10. Major depressive disorder.mp. 11. MDD.mp. 12. or/54-59 13. Posttraumatic stress disorder/ 14. (Post adj2 traumatic stress).mp. 15. (Post adj2 traumatic syndrome).mp. 16. Posttraumatic stress.mp. 17. PTSD.mp. 18. or/61-65 19. Anxiety disorders/ 20. Anxiety disorder*.mp. 21. Anxiety*.mp. 22. Generalized anxiety.mp. 23. Generalized anxiety disorder*.mp. 24. GAD.mp. 25. or/67-72 26. Exp Adaptation, Psychological/ 27. Exp Posttraumatic Growth, Psychological/ 28. Exp Social Determinants of Health/ 29. Exp Sense of Coherence/ 30. (well-being or wellness or cop* or quality of life or self-esteem or self-efficacy or behaviour or behavior or adapt* or skill*).mp 31. or/74-78 32. 53 or 60 or 66 or 73 or 79 |
| All | 1. 5 and 20 and 48 and 80 |

No limiters or restrictions were applied to any database searches.

**Table S2.** Sources and dates for grey literature and key journal searches

| **Source** | **Date searched** | **Citations screened by title and abstract** | **Records included in the scoping review** |
| --- | --- | --- | --- |
| *Grey literature* | | | |
| Center for Disease Control | January 1, 2021 | 2 | 0 |
| Google | January 1, 2021 | 18 | 1 |
| Google Scholar | January 1, 2021 | 10 | 3 |
| National Health Council | January 1, 2021 | 2 | 0 |
| National Institute for Communicable Diseases | January 1, 2021 | 1 | 0 |
| National Institute for Health and Care Excellence | January 1, 2021 | 1 | 0 |
| medRxiv | January 1, 2021 | 4 | 2 |
| ProQuest | January 1, 2021 | 84 | 0 |
| World Health Organization | January 1, 2021 | 3 | 0 |
| *Key journals* | | | |
| Journal of the American Medical Association | January 1, 2021 | 17 | 7 |
| New England Journal of Medicine | January 1, 2021 | 3 | 0 |
| The Lancet | January 1, 2021 | 14 | 1 |

^1^The preprint server for Health Sciences, operated by Cold Spring Harbor Laboratory

^2^The official of dissertation and theses repository of the Library of Congress

**Table S3.** Characteristics of included records

| **Research type** | **Record type** | **Country** | **Clinical specialty** | **Population** | **COVID-19 patients** | **First author** | **Year** |
| --- | --- | --- | --- | --- | --- | --- | --- |
| Primary research | Case report or case series | Canada | Oncology | Professionals |  | Stilos, K. | 2020 |
| Primary research | Case report or case series | Germany | Palliative care | Patients, families, and professionals | 1 | Anneser, J. | 2020 |
| Primary research | Case report or case series | Italy | Cardiology | Professionals |  | Agostoni, P. | 2020 |
| Primary research | Case report or case series | Italy | Intensive care | Professionals |  | Lissoni, B. | 2020 |
| Primary research | Case report or case series | Italy | Oncology | Professionals |  | Zeneli, A. | 2020 |
| Primary research | Case report or case series | Italy | Palliative care | Professionals |  | Tanzi, S. | 2020 |
| Primary research | Case report or case series | Japan | Intensive Care | Patients | 1 | Soh, M. | 2020 |
| Primary research | Case report or case series | Jordan | Obstetrics & Gynecology | Professionals | 4 | Alsharaydeh, I. | 2020 |
| Primary research | Case report or case series | NR | All settings | Professionals |  | Morley, G. | 2020 |
| Primary research | Case report or case series | Spain | Neurology | Professionals |  | Benaque, A. | 2020 |
| Primary research | Case report or case series | United Kingdom | Intensive care | Professionals |  | Webb, H. | 2020 |
| Primary research | Case report or case series | United Kingdom | Palliative care | Professionals |  | Fusi-Schmidhauser, T. | 2020 |
| Primary research | Case report or case series | United States | All settings | Patients and professionals | 3 | Sinvani, L. | 2020 |
| Primary research | Case report or case series | United States | All settings | Professionals |  | Hart, J.L. | 2020 |
| Primary research | Case report or case series | United States | All settings | Professionals |  | Overton, J. | 2020 |
| Primary research | Case report or case series | United States | All settings | Professionals |  | Wei, E. | 2020 |
| Primary research | Case report or case series | United States | Cardiology | Professionals |  | Rubin, G.A. | 2020 |
| Primary research | Case report or case series | United States | Intensive care | Professionals |  | Dhala, A. | 2020 |
| Primary research | Case report or case series | United States | Intensive care | Professionals |  | Griffin, K.M. | 2020 |
| Primary research | Case report or case series | United States | Neurology | Professionals |  | Leira, E.C. | 2020 |
| Primary research | Case report or case series | United States | Obstetrics & Gynecology | Professionals | 81 | London, V. | 2020 |
| Primary research | Case report or case series | United States | Palliative care | Patients and professionals | 1 | Ritchey, K. C. | 2020 |
| Primary research | Case report or case series | United States | Palliative care | Families and professionals |  | Holland, D.E. | 2020 |
| Primary research | Case report or case series | United States | Palliative care | Professionals |  | Hron, J.D. | 2020 |
| Primary research | Case report or case series | United States | Palliative care | Patients and professionals | 1 | Pahuja, M. | 2020 |
| Primary research | Case report or case series | United States | Palliative care | Professionals |  | Rosa, W. E. | 2020 |
| Primary research | Case report or case series | United States | Palliative care | Professionals |  | Schoenherr, L.A. | 2020 |
| Primary research | Case report or case series | United States | Palliative care | Professionals |  | Wallace, C.L. | 2020 |
| Primary research | Case report or case series | United States | Psychiatry | Patients | 2 | Fahed, M. | 2020 |
| Primary research | Cross-sectional survey | China | COVID-19 isolation | Professionals |  | Chen, H. | 2020 |
| Primary research | Cross-sectional survey | France | Intensive care | Professionals |  | Azoulay, E. | 2020 |
| Primary research | Cross-sectional survey | France | Intensive care | Professionals |  | Azoulay, E. | 2020 |
| Primary research | Cross-sectional survey | Germany | Oncology | Patients and professionals |  | Buntzel, J. | 2020 |
| Primary research | Cross-sectional survey | Global | Obstetrics & Gynecology | Professionals |  | Semaan, A. | 2020 |
| Primary research | Cross-sectional survey | Global | Palliative care | Professionals |  | Janssen, D.J.A. | 2020 |
| Primary research | Cross-sectional survey | India | Oncology | Patients |  | Mitra, M. | 2020 |
| Primary research | Cross-sectional survey | India | Palliative care | Professionals |  | Jain, T. | 2020 |
| Primary research | Cross-sectional survey | Italy | Obstetrics & Gynecology | Patients | 24 | Molgora, S. | 2020 |
| Primary research | Cross-sectional survey | Italy | Palliative care | Professionals |  | Costantini, M. | 2020 |
| Primary research | Cross-sectional survey | Jordan | COVID-19 isolation | Patients | 66 | Samrah, S.M. | 2020 |
| Primary research | Cross-sectional survey | Singapore | Oncology | Patients, families, and professionals |  | Ng, K.Y.Y. | 2020 |
| Primary research | Cross-sectional survey | Turkey | All settings | Professionals |  | Bostan, S. | 2020 |
| Primary research | Cross-sectional survey | United Kingdom | Obstetrics & Gynecology | Patients | 29 | Karavadra, B. | 2020 |
| Primary research | Cross-sectional survey | United States | All settings | Professionals |  | Arnetz, J.E. | 2020 |
| Primary research | Cross-sectional survey | United States | Obstetrics & Gynecology | Professionals |  | Peña, J.A. | 2020 |
| Primary research | Cross-sectional survey | United States | Oncology | Professionals |  | BrintzenhofeSzoc, K. | 2020 |
| Primary research | Cross-sectional survey | United States | Palliative Care | Families and professionals | 44 | Kuntz, J.G. | 2020 |
| Primary research | Cross-sectional survey + Semi-structured interviews | Israel | COVID-19 isolation | Patients and families | 90 | Dorman-Ilan, S. | 2020 |
| Primary research | Cross-sectional Survey + Semi-structured Interviews | Pakistan | COVID-19 isolation | Patients |  | Rizvi J. | 2020 |
| Primary research | Cross-sectional survey + Semi-structured interviews | United Kingdom | All settings | Patients, families, and professionals |  | British Red Cross | 2020 |
| Primary research | Cross-sectional survey + Semi-structured interviews | United States | All settings | Professionals |  | Hennein, R. | 2020 |
| Primary research | Cross-sectional Survey + Semi-structured Interviews | United States | Intensive care | Families |  | Sasangohar, F. | 2020 |
| Primary research | Retrospective cohort study | Japan | All settings | Patients | 10 | Kandori, K. | 2020 |
| Primary research | Retrospective cohort study | United States | Palliative care | Patients, families, and professionals |  | Lai, L. | 2020 |
| Primary research | Retrospective Cohort Study + Semi-structured Interviews | United States | Surgery & Emergency | Professionals |  | Zeh, R.D. | 2020 |
| Primary research | Semi-structured interviews | Canada | All settings | Patients, families, and professionals | 7 | Cook, D.J. | 2020 |
| Primary research | Semi-structured interviews | China | All settings | Professionals |  | Jia, Y. | 2020 |
| Primary research | Semi-structured interviews | China | COVID-19 isolation | Patients | 16 | Sun, N. | 2020 |
| Primary research | Semi-structured interviews | China | COVID-19 isolation | Professionals |  | Tan, R. | 2020 |
| Primary research | Semi-structured interviews | India | COVID-19 isolation | Professionals |  | Mohindra, R. | 2020 |
| Primary research | Semi-structured interviews | India | Oncology | Patients and families |  | Atreya, S. | 2020 |
| Primary research | Semi-structured interviews | Iran | COVID-19 isolation | Professionals |  | Karimi, Z. | 2020 |
| Primary research | Semi-structured interviews | Italy | Palliative care | Families |  | Mercadante, S. | 2020 |
| Primary research | Semi-structured interviews | The Netherlands | Psychiatry | Patients | 2 | DeJong, A.J. | 2020 |
| Primary research | Semi-structured interviews | United States | All settings | Professionals |  | Butler, C.R. | 2020 |
| Primary research | Semi-structured interviews | United States | Neurology | Patients and families |  | Creutzfeldt, C.J. | 2020 |
| Secondary research | Expert recommendations | Canada | All settings | Patients, families, and professionals |  | Canadian Foundation for Healthcare Improvement | 2020 |
| Secondary research | Expert recommendations | Canada | All settings | Professionals |  | McMahon, M. | 2020 |
| Secondary research | Expert recommendations | Germany | Palliative care | Professionals |  | Munch, U. | 2020 |
| Secondary research | Expert recommendations | India | Palliative care | Professionals |  | Damani, A. | 2020 |
| Secondary research | Expert recommendations | Singapore | Oncology | Professionals |  | Tey, J. | 2020 |
| Secondary research | Expert recommendations | United States | All settings | Professionals |  | Cohen, S.P. | 2020 |
| Secondary research | Expert recommendations | United States | Intensive care | Professionals |  | Owens, I.T. | 2020 |
| Secondary research | Expert recommendations | United States | Neurology | Professionals |  | Rubin, M.A. | 2020 |
| Secondary research | Literature review | Australia | Palliative care | Professionals |  | Cabarkapa, S. | 2020 |
| Secondary research | Literature review | Canada | Palliative care | Professionals |  | Arya, A. | 2020 |
| Secondary research | Literature review | France | All settings | Professionals |  | Chevance, A. | 2020 |
| Secondary research | Literature review | India | Palliative care | Professionals |  | Rao, S.R. | 2020 |
| Secondary research | Literature review | Israel | Intensive care | Professionals |  | Sprung, C.L. | 2020 |
| Secondary research | Literature review | Italy | All settings | Professionals |  | Mistraletti, G. | 2020 |
| Secondary research | Literature review | Poland | Intensive care | Professionals |  | Kotfis, K. | 2020 |
| Secondary research | Literature review | Singapore | COVID-19 isolation | Professionals |  | Fan, P.E.M. | 2020 |
| Secondary research | Literature review | United Arab Emirates | Oncology | Professionals |  | Al-Shamsi, H.O. | 2020 |
| Secondary research | Literature review | United Kingdom | Palliative care | Professionals |  | Etkind, S.N. | 2020 |
| Secondary research | Literature review | United Kingdom | Palliative care | Professionals |  | Selman, L.E. | 2020 |
| Secondary research | Literature review | United Kingdom | Surgery & Emergency | Professionals |  | Al-Jabir, A. | 2020 |
| Secondary research | Literature review | United States | All settings | Professionals |  | Anderson-Shaw, L.K. | 2020 |
| Secondary research | Literature review | United States | All settings | Professionals |  | Dayal, A.K. | 2020 |
| Secondary research | Literature review | United States | All settings | Professionals |  | Feinstein, R.E. | 2020 |
| Secondary research | Literature review | United States | All settings | Professionals |  | Gupta, A. | 2020 |
| Secondary research | Literature review | United States | All settings | Professionals |  | Yamamoto, V. | 2020 |
| Secondary research | Literature review | United States | Intensive care | Professionals |  | Devlin, J.W. | 2020 |
| Secondary research | Literature review | United States | Oncology | Professionals |  | Alhalabi, O. | 2020 |
| Secondary research | Literature review | United States | Palliative care | Professionals |  | Fadul, N. | 2020 |
| Secondary research | Literature review | United States | Palliative care | Professionals |  | Wynne, K.J. | 2020 |
| Grey literature | Commentary, editorial or opinion | Argentina | All settings | Professionals |  | Belli, L.F. | 2020 |
| Grey literature | Commentary, editorial or opinion | Australia | All settings | Professionals |  | Chew, C. | 2020 |
| Grey literature | Commentary, editorial or opinion | Canada | All settings | Professionals |  | Bourgeault, I.L. | 2020 |
| Grey literature | Commentary, editorial or opinion | Canada | Allergy and immunology | Professionals |  | Abrams, E.M. | 2020 |
| Grey literature | Commentary, editorial or opinion | China | All settings | Professionals |  | Feng, D. | 2020 |
| Grey literature | Commentary, editorial or opinion | China | Palliative care | Professionals |  | Lam, P.T. | 2020 |
| Grey literature | Commentary, editorial or opinion | China | Psychiatry | Professionals |  | Chen, J. | 2020 |
| Grey literature | Commentary, editorial or opinion | France | Obstetrics & Gynecology | Professionals |  | Viaux, S. | 2020 |
| Grey literature | Commentary, editorial or opinion | Ireland | Psychiatry | Professionals |  | Glancy, D. | 2020 |
| Grey literature | Commentary, editorial or opinion | Italy | Intensive care | Patients, families, and professionals |  | Bambi, S. | 2020 |
| Grey literature | Commentary, editorial or opinion | Italy | Oncology | Professionals |  | Pietrantonio, F. | 2020 |
| Grey literature | Commentary, editorial or opinion | Italy | Palliative care | Professionals |  | Romano, M. | 2020 |
| Grey literature | Commentary, editorial or opinion | Mexico | Psychiatry | Professionals |  | Landa-Ramirez, E. | 2020 |
| Grey literature | Commentary, editorial or opinion | NR | All settings | Professionals |  | Morley, G. | 2020 |
| Grey literature | Commentary, editorial or opinion | NR | Intensive care | Professionals |  | Moore, B. | 2020 |
| Grey literature | Commentary, editorial or opinion | Singapore | Intensive care | Professionals |  | Liew, M.F. | 2020 |
| Grey literature | Commentary, editorial or opinion | Spain | Intensive care | Professionals |  | Estella, A. | 2020 |
| Grey literature | Commentary, editorial or opinion | Switzerland | Obstetrics & Gynecology | Professionals |  | Horsch, A. | 2020 |
| Grey literature | Commentary, editorial or opinion | Thailand | All settings | Professionals |  | Turale, S. | 2020 |
| Grey literature | Commentary, editorial or opinion | United Kingdom | Palliative care | Professionals |  | Cheng, J.O.S. | 2020 |
| Grey literature | Commentary, editorial or opinion | United Kingdom | Palliative care | Professionals |  | Knights, D. | 2020 |
| Grey literature | Commentary, editorial or opinion | United Kingdom | Palliative care | Professionals |  | Yardley, S. | 2020 |
| Grey literature | Commentary, editorial or opinion | United Kingdom | Surgery & Emergency | Professionals |  | Arjomandi Rad, A. | 2020 |
| Grey literature | Commentary, editorial or opinion | United Kingdom | Palliative care | Professionals |  | Moore, K.J. | 2020 |
| Grey literature | Commentary, editorial or opinion | United States | All settings | Professionals |  | Cho, H.J. | 2020 |
| Grey literature | Commentary, editorial or opinion | United States | All settings | Professionals |  | Ferrell, B.R. | 2020 |
| Grey literature | Commentary, editorial or opinion | United States | All settings | Professionals |  | Frampton, S. | 2020 |
| Grey literature | Commentary, editorial or opinion | United States | All settings | Professionals |  | Garrett, J.R. | 2020 |
| Grey literature | Commentary, editorial or opinion | United States | All settings | Professionals |  | Hall, D.E. | 2020 |
| Grey literature | Commentary, editorial or opinion | United States | All settings | Professionals |  | Hossain, F. | 2020 |
| Grey literature | Commentary, editorial or opinion | United States | All settings | Professionals |  | Lau, J. | 2020 |
| Grey literature | Commentary, editorial or opinion | United States | All settings | Professionals |  | Lee, D.R. | 2020 |
| Grey literature | Commentary, editorial or opinion | United States | All settings | Professionals |  | McKee, M. | 2020 |
| Grey literature | Commentary, editorial or opinion | United States | All settings | Professionals |  | Fang, J. | 2020 |
| Grey literature | Commentary, editorial or opinion | United States | Cardiology | Professionals |  | Stephens, E.H. | 2020 |
| Grey literature | Commentary, editorial or opinion | United States | Catholic Bioethics | Professionals |  | The National Catholic Bioethics Center | 2020 |
| Grey literature | Commentary, editorial or opinion | United States | Intensive care | Professionals |  | Dudzinski, D.M. | 2020 |
| Grey literature | Commentary, editorial or opinion | United States | Intensive care | Professionals |  | Montauk, T.R. | 2020 |
| Grey literature | Commentary, editorial or opinion | United States | Intensive care | Professionals |  | Tingey, J.L. | 2020 |
| Grey literature | Commentary, editorial or opinion | United States | Neurology | Patients | 6 | Valdes, E. | 2020 |
| Grey literature | Commentary, editorial or opinion | United States | Neurology | Professionals |  | Correa, D.J. | 2020 |
| Grey literature | Commentary, editorial or opinion | United States | Obstetrics & Gynecology | Professionals |  | Arora, K.S. | 2020 |
| Grey literature | Commentary, editorial or opinion | United States | Obstetrics & Gynecology | Professionals |  | Choi, K.R. | 2020 |
| Grey literature | Commentary, editorial or opinion | United States | Obstetrics & Gynecology | Professionals |  | Ott, M.A. | 2020 |
| Grey literature | Commentary, editorial or opinion | United States | Obstetrics & Gynecology | Professionals |  | Stephens, A.J. | 2020 |
| Grey literature | Commentary, editorial or opinion | United States | Palliative care | Professionals |  | Abbott, J. | 2020 |
| Grey literature | Commentary, editorial or opinion | United States | Palliative care | Professionals |  | Apoeso, O. | 2020 |
| Grey literature | Commentary, editorial or opinion | United States | Palliative care | Professionals |  | Chua, I.S. | 2020 |
| Grey literature | Commentary, editorial or opinion | United States | Palliative care | Professionals |  | Fausto, J. | 2020 |
| Grey literature | Commentary, editorial or opinion | United States | Palliative care | Professionals |  | Mehta, A.K. | 2020 |
| Grey literature | Commentary, editorial or opinion | United States | Palliative care | Professionals |  | Morris, S.E. | 2020 |
| Grey literature | Commentary, editorial or opinion | United States | Palliative care | Professionals |  | Rosa, W.E. | 2020 |
| Grey literature | Commentary, editorial or opinion | United States | Psychiatry | Professionals |  | Cherepanov, E. | 2020 |
| Grey literature | Commentary, editorial or opinion | United States | Psychiatry | Professionals |  | LeBlanc, L.A. | 2020 |
| Grey literature | Online web article or blog post | Canada | Oncology | Professionals |  | Nelson, R. | 2020 |
| Grey literature | Online web article or blog post | Italy | COVID-19 isolation | Professionals |  | Brucato, A. | 2020 |
| Grey literature | Online web article or blog post | NR | All settings | Professionals |  | Ford, S. | 2020 |
| Grey literature | Online web article or blog post | United States | All settings | Professionals |  | Cleveland Clinic | 2020 |
| Grey literature | Online web article or blog post | United States | COVID-19 isolation | Patients | 1 | Evans, G. | 2020 |
| Grey literature | Online web article or blog post | United States | Intensive care | Professionals |  | Maves, R.C. | 2020 |

Organized in order of research type; record type; country; then clinical specialty

**Table S4.** Outcomes reported from included records

|  |  | **Impacts of restricted visitation^1^** | | | | | **Perspectives from restricted visitation^2^** | | | | |
| --- | --- | --- | --- | --- | --- | --- | --- | --- | --- | --- | --- |
| **First author** | **Restricted visitation policy** | **Neurocognitive** | **Mental Health** | **Quality of life & Well-being** | **Coping & Daily Functioning** | **Overwhelming Grief** | | **State of Well-being** | **Connection & Communication** | **Coping & Accessing Support** | **Touch & Physical Presence** |
| Agostoni, P. | No visitors, no exceptions |  |  |  |  |  | | **✓** |  |  |  |
| Al-Jabir, A. | No visitors, no exceptions |  |  |  |  |  | |  |  | **✓** |  |
| Alhalabi, O. | No visitors, no exceptions |  |  |  |  |  | |  | **✓** | **✓** |  |
| Benaque, A. | No visitors, no exceptions |  |  | **✓** | **✓** |  | |  | **✓** |  |  |
| Bostan, S. | No visitors, no exceptions |  | **✓** |  |  |  | |  |  |  |  |
| Bourgeault, I.L. | No visitors, no exceptions |  |  |  |  | **✓** | | **✓** | **✓** |  | **✓** |
| Brucato, A. | No visitors, no exceptions |  |  |  |  |  | | **✓** | **✓** | **✓** |  |
| Chen, J. | No visitors, no exceptions |  |  |  |  |  | | **✓** | **✓** |  |  |
| Cheng, J.O.S. | No visitors, no exceptions |  |  |  |  | **✓** | | **✓** | **✓** | **✓** | **✓** |
| Chew, C. | No visitors, no exceptions |  |  |  |  | **✓** | |  | **✓** | **✓** | **✓** |
| Cohen, S.P. | No visitors, no exceptions |  |  |  |  |  | | **✓** |  | **✓** |  |
| Creutzfeldt, C.J. | No visitors, no exceptions |  |  |  |  | **✓** | |  | **✓** | **✓** | **✓** |
| Dhala, A. | No visitors, no exceptions |  |  |  |  |  | |  | **✓** |  |  |
| Dorman-Ilan, S. | No visitors, no exceptions |  | **✓** | **✓** | **✓** |  | | **✓** | **✓** | **✓** |  |
| Dudzinski, D.M. | No visitors, no exceptions |  |  |  |  | **✓** | | **✓** |  | **✓** |  |
| Evans, G. | No visitors, no exceptions |  |  |  |  |  | | **✓** |  |  | **✓** |
| Fadul, N. | No visitors, no exceptions |  |  |  |  | **✓** | |  | **✓** |  |  |
| Fan, P.E.M. | No visitors, no exceptions |  |  |  |  |  | | **✓** | **✓** | **✓** |  |
| Fang, J. | No visitors, no exceptions |  |  |  |  |  | | **✓** | **✓** |  |  |
| Fausto, J. | No visitors, no exceptions |  |  |  |  |  | | **✓** |  | **✓** |  |
| Feinstein, R.E. | No visitors, no exceptions |  |  |  |  |  | | **✓** |  | **✓** |  |
| Glancy, D. | No visitors, no exceptions |  |  |  |  |  | | **✓** |  | **✓** |  |
| Hennein, R. | No visitors, no exceptions |  | **✓** |  |  | **✓** | | **✓** | **✓** | **✓** |  |
| Horsch, A. | No visitors, no exceptions |  |  |  |  |  | | **✓** |  |  | **✓** |
| Jia, Y. | No visitors, no exceptions |  |  |  |  |  | | **✓** | **✓** | **✓** |  |
| Kandori, K. | No visitors, no exceptions | **✓** |  |  |  |  | |  |  |  |  |
| Karavadra, B. | No visitors, no exceptions |  |  |  |  |  | | **✓** |  | **✓** | **✓** |
| Karimi, Z. | No visitors, no exceptions |  |  |  |  | **✓** | |  |  | **✓** |  |
| Kuntz, J.G. | No visitors, no exceptions |  | **✓** | **✓** | **✓** |  | |  | **✓** | **✓** | **✓** |
| Lau, J. | No visitors, no exceptions |  |  |  |  | **✓** | | **✓** | **✓** | **✓** | **✓** |
| McKee, M. | No visitors, no exceptions |  |  |  |  |  | |  | **✓** |  |  |
| Mehta, A.K. | No visitors, no exceptions |  |  |  |  |  | | **✓** | **✓** |  |  |
| Mercadante, S. | No visitors, no exceptions |  |  |  |  | **✓** | |  |  |  | **✓** |
| Mohindra, R. | No visitors, no exceptions |  |  |  |  |  | | **✓** | **✓** | **✓** |  |
| Ng, K.Y.Y. | No visitors, no exceptions |  | **✓** | **✓** | **✓** |  | |  |  |  |  |
| Owens, I.T. | No visitors, no exceptions |  |  | **✓** |  | **✓** | | **✓** | **✓** |  | **✓** |
| Pahuja, M. | No visitors, no exceptions |  |  |  |  |  | | **✓** | **✓** | **✓** |  |
| Pietrantonio, F. | No visitors, no exceptions |  |  |  |  | **✓** | |  | **✓** | **✓** |  |
| Rao, S.R. | No visitors, no exceptions |  |  |  |  | **✓** | | **✓** | **✓** | **✓** | **✓** |
| Rosa, W. E. | No visitors, no exceptions |  |  | **✓** |  | **✓** | |  | **✓** | **✓** |  |
| Rubin, G.A. | No visitors, no exceptions |  |  |  |  |  | |  | **✓** |  |  |
| Rubin, M.A. | No visitors, no exceptions |  |  |  |  |  | |  | **✓** | **✓** |  |
| Sasangohar, F. | No visitors, no exceptions |  | **✓** | **✓** | **✓** |  | |  |  |  |  |
| Sinvani, L. | No visitors, no exceptions | **✓** |  |  |  | **✓** | | **✓** | **✓** | **✓** |  |
| Soh, M. | No visitors, no exceptions |  | **✓** |  |  |  | |  |  |  |  |
| Sun, N. | No visitors, no exceptions |  |  |  |  | **✓** | | **✓** | **✓** | **✓** | **✓** |
| Tan, R. | No visitors, no exceptions |  |  |  |  |  | | **✓** |  | **✓** |  |
| Tanzi, S. | No visitors, no exceptions |  |  |  |  |  | | **✓** | **✓** | **✓** |  |
| Turale, S. | No visitors, no exceptions |  |  |  |  | **✓** | | **✓** |  | **✓** |  |
| Wei, E. | No visitors, no exceptions |  |  |  |  | **✓** | | **✓** | **✓** |  |  |
| Zeh, R.D. | No visitors, no exceptions |  | **✓** | **✓** | **✓** |  | | **✓** | **✓** | **✓** |  |
| Abbott, J. | No visitors, exception end-of-life |  |  |  |  |  | |  | **✓** | **✓** |  |
| Anneser, J. | No visitors, exception end-of-life |  |  |  |  | **✓** | |  | **✓** |  |  |
| Apoeso, O. | No visitors, exception end-of-life |  |  |  |  |  | | **✓** |  |  |  |
| Bambi, S. | No visitors, exception end-of-life | **✓** | **✓** | **✓** | **✓** | **✓** | | **✓** | **✓** | **✓** | **✓** |
| BrintzenhofeSzoc. | No visitors, exception end-of-life |  | **✓** | **✓** | **✓** |  | |  | **✓** | **✓** |  |
| British Red Cross | No visitors, exception end-of-life |  | **✓** | **✓** | **✓** |  | |  | **✓** | **✓** |  |
| Cleveland Clinic | No visitors, exception end-of-life |  |  |  |  |  | | **✓** | **✓** | **✓** |  |
| Correa, D.J. | No visitors, exception end-of-life |  |  |  |  |  | |  |  |  |  |
| Dayal, A.K. | No visitors, exception end-of-life |  |  |  |  |  | |  | **✓** |  |  |
| Estella, A. | No visitors, exception end-of-life |  |  |  |  |  | | **✓** | **✓** |  |  |
| Fahed, M. | No visitors, exception end-of-life |  |  |  |  |  | | **✓** |  | **✓** |  |
| Fusi-Schmidhauser, T. | No visitors, exception end-of-life |  |  |  |  | **✓** | |  |  | **✓** |  |
| Griffin, K.M. | No visitors, exception end-of-life |  |  |  |  |  | | **✓** |  | **✓** |  |
| Hall, D.E. | No visitors, exception end-of-life |  |  |  |  | **✓** | |  | **✓** | **✓** |  |
| Moore, B. | No visitors, exception end-of-life |  |  |  |  | **✓** | | **✓** | **✓** | **✓** | **✓** |
| Overton, J. | No visitors, exception end-of-life |  |  |  |  | **✓** | |  |  |  |  |
| Ritchey, K. C. | No visitors, exception end-of-life |  |  |  |  | **✓** | | **✓** | **✓** | **✓** | **✓** |
| Selman, L.E. | No visitors, exception end-of-life |  |  |  |  | **✓** | | **✓** | **✓** | **✓** | **✓** |
| Stilos, K. | No visitors, exception end-of-life |  |  |  |  | **✓** | | **✓** | **✓** | **✓** | **✓** |
| Valdes, E. | No visitors, exception end-of-life |  |  |  |  | **✓** | |  | **✓** |  |  |
| Viaux, S. | No visitors, exception end-of-life |  |  |  |  |  | | **✓** |  |  |  |
| Wallace, C.L. | No visitors, exception end-of-life |  |  |  |  | **✓** | | **✓** | **✓** | **✓** |  |
| Webb, H. | No visitors, exception end-of-life |  |  |  | **✓** | **✓** | | **✓** | **✓** | **✓** |  |
| Yardley, S. | No visitors, exception end-of-life |  |  |  |  | **✓** | | **✓** | **✓** | **✓** | **✓** |
| Arora, K.S. | One visitor, at any time |  |  |  |  |  | | **✓** |  | **✓** |  |
| Cook, D.J. | One visitor, at any time |  | **✓** | **✓** | **✓** | **✓** | | **✓** | **✓** | **✓** | **✓** |
| Feng, D. | One visitor, at any time |  |  |  |  |  | |  |  |  |  |
| Lee, D.R. | One visitor, at any time |  |  |  |  | **✓** | | **✓** | **✓** | **✓** |  |
| Leira, E.C. | One visitor, at any time |  |  |  |  |  | |  | **✓** |  |  |
| Liew, M.F. | One visitor, at any time |  |  |  |  |  | | **✓** |  |  |  |
| London, V. | One visitor, at any time |  |  |  |  |  | | **✓** |  | **✓** |  |
| Montauk, T.R. | One visitor, at any time |  |  |  |  | **✓** | | **✓** | **✓** | **✓** |  |
| Nelson, R. | One visitor, at any time |  |  |  |  | **✓** | | **✓** | **✓** | **✓** |  |
| Pena, J.A. | One visitor, at any time |  |  |  |  |  | |  |  | **✓** |  |
| Tey, J. | One visitor, at any time |  |  |  |  |  | |  | **✓** | **✓** |  |
| Alsharaydeh, I. | Many visitors, at any time |  |  |  |  |  | |  |  | **✓** |  |
| Zeneli, A. | Many visitors, at any time |  |  |  |  |  | |  | **✓** | **✓** |  |
| Abrams, E.M. | Unspecified |  |  |  |  |  | |  | **✓** | **✓** |  |
| Al-Shamsi, H.O. | Unspecified |  |  |  |  |  | | **✓** | **✓** | **✓** |  |
| Anderson-Shaw, L.K. | Unspecified |  |  |  |  | **✓** | | **✓** | **✓** | **✓** |  |
| Arjomandi Rad, A. | Unspecified |  |  |  |  |  | | **✓** | **✓** | **✓** |  |
| Arnetz, J.E. | Unspecified |  |  | **✓** |  |  | |  |  | **✓** |  |
| Arya, A. | Unspecified |  |  |  |  | **✓** | |  | **✓** | **✓** |  |
| Atreya, S. | Unspecified |  | **✓** | **✓** | **✓** |  | | **✓** | **✓** | **✓** |  |
| Azoulay, E. | Unspecified | **✓** | **✓** | **✓** | **✓** |  | |  |  |  |  |
| Azoulay, E. | Unspecified |  | **✓** | **✓** |  |  | |  |  |  |  |
| Belli, L.F. | Unspecified |  |  |  |  | **✓** | | **✓** |  | **✓** |  |
| Buntzel, J. | Unspecified |  | **✓** | **✓** | **✓** |  | |  |  |  |  |
| Butler, C.R. | Unspecified |  |  |  |  | **✓** | | **✓** | **✓** | **✓** |  |
| Cabarkapa, S. | Unspecified |  |  |  |  |  | | **✓** | **✓** | **✓** |  |
| Canadian Foundation for Healthcare Improvement | Unspecified |  |  |  |  |  | | **✓** |  |  |  |
| Chen, H. | Unspecified |  |  |  | **✓** |  | |  | **✓** |  |  |
| Cherepanov, E. | Unspecified |  |  |  |  | **✓** | | **✓** |  |  |  |
| Chevance, A. | Unspecified |  |  |  |  |  | | **✓** |  |  | **✓** |
| Cho, H.J. | Unspecified |  |  |  |  |  | |  | **✓** |  |  |
| Choi, K.R. | Unspecified |  |  |  |  |  | |  |  | **✓** |  |
| Chua, I.S. | Unspecified |  |  |  |  | **✓** | | **✓** | **✓** | **✓** | **✓** |
| Costantini, M. | Unspecified |  |  |  | **✓** | **✓** | |  | **✓** | **✓** |  |
| Damani, A. | Unspecified |  |  |  |  | **✓** | |  | **✓** | **✓** |  |
| DeJong, A.J. | Unspecified |  |  |  |  |  | | **✓** | **✓** |  |  |
| Devlin, J.W. | Unspecified |  |  |  |  | **✓** | |  |  | **✓** | **✓** |
| Etkind, S.N. | Unspecified |  |  |  |  | **✓** | | **✓** |  | **✓** |  |
| Ferrell, B.R. | Unspecified |  |  |  |  | **✓** | |  | **✓** | **✓** |  |
| Ford, S. | Unspecified |  |  |  |  | **✓** | |  |  | **✓** |  |
| Frampton, S. | Unspecified |  |  |  |  |  | | **✓** | **✓** | **✓** |  |
| Garrett, J.R. | Unspecified |  |  |  |  |  | | **✓** | **✓** |  |  |
| Gupta, A. | Unspecified |  |  |  |  |  | |  | **✓** |  |  |
| Hart, J.L. | Unspecified |  |  |  |  |  | |  | **✓** | **✓** |  |
| Holland, D.E. | Unspecified |  |  |  |  | **✓** | | **✓** | **✓** | **✓** | **✓** |
| Hossain, F. | Unspecified |  |  |  |  | **✓** | | **✓** | **✓** | **✓** |  |
| Hron, J.D. | Unspecified |  |  |  |  |  | | **✓** | **✓** |  |  |
| Jain, T. | Unspecified |  |  |  |  |  | | **✓** |  | **✓** | **✓** |
| Janssen, D.J.A. | Unspecified |  |  |  |  | **✓** | | **✓** | **✓** | **✓** | **✓** |
| Knights, D. | Unspecified |  |  |  |  | **✓** | | **✓** |  |  | **✓** |
| Kotfis, K. | Unspecified |  |  |  |  |  | | **✓** | **✓** | **✓** |  |
| Lai, L. | Unspecified |  |  |  | **✓** |  | |  |  |  |  |
| Lam, P.T. | Unspecified |  |  |  |  | **✓** | |  |  | **✓** |  |
| Landa-Ramirez, E. | Unspecified |  |  |  |  |  | | **✓** | **✓** | **✓** | **✓** |
| LeBlanc, L.A. | Unspecified |  |  |  |  | **✓** | | **✓** | **✓** | **✓** |  |
| Lissoni, B. | Unspecified |  |  |  |  | **✓** | | **✓** |  | **✓** |  |
| Maves, R.C. | Unspecified |  |  |  |  | **✓** | | **✓** | **✓** | **✓** | **✓** |
| McMahon, M. | Unspecified |  |  |  |  |  | | **✓** |  |  |  |
| Mistraletti, G. | Unspecified |  |  |  |  | **✓** | | **✓** | **✓** | **✓** | **✓** |
| Mitra, M. | Unspecified |  | **✓** | **✓** | **✓** |  | |  |  |  |  |
| Molgora, S. | Unspecified |  | **✓** | **✓** | **✓** |  | |  |  |  |  |
| Moore, K.J. | Unspecified |  |  |  |  |  | |  |  |  | **✓** |
| Morley, G. | Unspecified |  |  |  |  | **✓** | | **✓** | **✓** | **✓** |  |
| Morley, G. | Unspecified |  |  |  |  | **✓** | | **✓** | **✓** | **✓** | **✓** |
| Morris, S.E. | Unspecified |  |  |  |  |  | |  | **✓** |  |  |
| Munch, U. | Unspecified |  |  |  |  | **✓** | | **✓** | **✓** | **✓** |  |
| Ott, M.A. | Unspecified |  |  |  |  |  | | **✓** |  |  |  |
| Rizvi J. | Unspecified |  |  |  |  |  | | **✓** | **✓** | **✓** |  |
| Romano, M. | Unspecified |  |  |  |  | **✓** | | **✓** | **✓** | **✓** |  |
| Rosa, W.E. | Unspecified |  |  |  |  |  | | **✓** | **✓** | **✓** | **✓** |
| Samrah, S.M. | Unspecified |  | **✓** |  | **✓** |  | |  |  |  |  |
| Schoenherr, L.A. | Unspecified |  |  |  |  | **✓** | | **✓** | **✓** | **✓** |  |
| Semaan, A. | Unspecified |  | **✓** | **✓** | **✓** |  | |  |  |  |  |
| Sprung, C.L. | Unspecified |  |  |  |  |  | | **✓** | **✓** |  |  |
| Stephens, A.J. | Unspecified |  |  |  |  |  | |  |  | **✓** | **✓** |
| Stephens, E.H. | Unspecified |  |  |  |  |  | | **✓** | **✓** |  |  |
| The Catholic Bioethics Center | Unspecified |  |  |  |  | **✓** | | **✓** | **✓** | **✓** |  |
| Tingey, J.L. | Unspecified |  |  |  |  |  | | **✓** | **✓** |  |  |
| Wynne, K.J. | Unspecified |  |  |  |  | **✓** | | **✓** | **✓** | **✓** |  |
| Yamamoto, V. | Unspecified |  |  |  |  |  | | **✓** | **✓** | **✓** |  |

Organized in order of restricted visitation policy; first author last name

Red text indicates primary research record

^1^Categories determined from data charting of included records

^2^Themes determined from inductive thematic analysis of included records

**Table S5.** Summary of evidence and important approaches to mitigate impact of restricted visitation during the COVID-19 pandemic

| **Questions used to guide evidence synthesis^1^** | **Patients** | **Family members** | **Healthcare professionals** |
| --- | --- | --- | --- |
|  | **N=51** | **N=61** | **N=40** |
| Steps or guiding principles described | 41 (80.4%) | 51 (83.6%) | 37 (92.5%) |
| Derived from empirical evidence in full or part | 29 (56.9%) | 37 (60.7%) | 26 (65.0%) |
| Minimum expertise considered | 15 (29.4%) | 21 (34.4%) | 16 (40.0%) |
| Limitations reported | 14 (27.5%) | 16 (26.2%) | 10 (25.0%) |
| Approach is reproducible | 26 (51.0%) | 37 (61.0%) | 23 (56.0%) |
| Approach can be feasibly applied to other contexts | 26 (51.0%) | 36 (59.0%) | 23 (56.0%) |
| Total score^1^ (median, IQR) | 3 (2-4) | 4 (3-5) | 4 (3-5) |

| **First author** | **Population^1^** | **Description** | **Insights** | **Score^2^** |
| --- | --- | --- | --- | --- |
| Abbott, J. | Patients, families | Focused plan and crisis standards for palliative care and hospice services | Patients and families should be allowed to undertake reasonable risks of visitation with dying loved ones, with the understanding that this exposure could result in potential illness among visitors or may entail subsequent quarantine. | 6 |
| Arya, A. | Professionals | Multipronged approach focused on 'stuff, staff, space, systems, sedation, separation, communication, and equity' to provide quality palliative care | A multipronged approach can guide planning and ensure that the palliative care needs of patients and their family members are met. | 5 |
| Belli, L.F. | Families, professionals | Four 'moments' to guide the transmission of bad news at a distance | A comprehensive and structured approach reduces negative impact that difficult conversations over telephone have on health professionals. | 5 |
| Benaque, A. | Patients, families, professionals | Healthcare contingency plan | The contingency model of care helped to guarantee the continuity of care while preserving the safety of patients, families, and professionals. | 5 |
| Dhala, A. | Patients, families | Virtual Intensive Care Unit (vICU) | Virtual family visitation via the Consultant Bridge application, palliative care delivery, and specialist consultation for patients with COVID-19 exemplify the successful adaptation of the vICU implementation. | 5 |
| Fang, J. | Patients, families | Factors and features for videoconferencing applications | Expansion of in-hospital telehealth capabilities requires adapting existing systems based on these design principles for individual hospitals. | 5 |
| Feinstein, R.E. | Professionals | Implementation of the 'Healthcare Worker Mental Health COVID‐19 Hotline' | A coherent approach to developing a COVID‐19 Mental Health Hotline was helpful and led to success. | 5 |
| Fusi-Schmidhauser, T. | Families | Tailored management plan for patients with COVID-19 who are not suitable for mechanical ventilation | Palliative care should be at the forefront to help make the best decisions, give care to families, and offer spiritual support. Nursing interventions and family involvement were adapted as per patients’ disease stage and infection control requirements. | 5 |
| Griffin, K.M. | Families, professionals | Communicating with families and facilitating staff wellness | A program that provides access to palliative care and ethics consultants, 24 hours a day, has helped facilitate conversations around end-of-life. | 5 |
| Hron, J.D. | Patients, families, professionals | Workflow for inpatient telepalliative consultation | Telepalliative skills without nonverbal techniques are novel and take time to develop and is a powerful tool that can promote good communication and high-quality care. | 6 |
| Kuntz, J.G. | Patients, families, professionals | Standard workflow enabling e-family meetings | Telemedicine for e-family meetings has strengthened the relationships between critical care and palliative care across our system particularly in the COVID-19 ICUs. | 5 |
| Landa-Ramirez, E. | Families, professionals | Intensive preparatory clinical actions in the emergency psychology department | It is important the psychology concerning the transmission of death notifications in the COVID-19 context. | 5 |
| Lissoni, B. | Families, professionals | Psychological interventions for ICU clinicians and families | The objective of psychological interventions in the acute phase is to sustain resources and reinforce the factors that may provide a protective action from posttraumatic stress and complicated grief. | 5 |
| Maves, R.C. | Patients, families, professionals | Operational steps to implement a triage system | Establishing the infrastructure necessary helped to equitably meet the clinical needs of the greatest number of patients with COVID-19 during a time of scarce resources. | 5 |

ICU, intensive care unit; IQR, interquartile range

Organized alphabetically by first author

Blue text indicates question satisfied in less than 50% of included reports that used an approach

^1^Included in the steps described in the record

^2^One point given for each of steps; derivation; expertise; limitations; operationalized; and generalizable

**Table S6.** Approaches to mitigate impact of restricted visitation policies during the COVID-19 pandemic

| **First author** | **Target^1*^** | **Description^2*^** | **Steps^3*^** | **Derivation^4*^** | **Expertise^5*^** | **Limitations^6*^** | **Operationalized^7#^** | **Generalizable^8#^** | **Insights^*^** | **Score^9^** |
| --- | --- | --- | --- | --- | --- | --- | --- | --- | --- | --- |
| Abbott, J. | Patients, families | Focused plan and crisis standards for palliative care and hospice services | 1. Actions to improve communication and documentation of patients’ goals of care and preferences for treatment prior to and during a COVID-19 surge 2. Plans to ensure alternative care processes and spaces for patients receiving comfort-focused care to accommodate expected surge over-flow from hospitals, nursing homes, and other care facilities 3. Create a virtual hotline for round-the-clock specialty-level advice and support to extend palliative care expertise during a surge | Empirical and theory | Training or education in palliative care | Resource limitations | Yes | Yes | Patients and families should be allowed to undertake reasonable risks of visitation with dying loved ones, with the understanding that this exposure could result in potential illness among visitors or may entail subsequent quarantine. | 6 |
| Hron, J.D. | Patients, families, professionals | Workflow for inpatient telepalliative consultation | 1. Confirm patient has in-room iPad or provides eligible patient an iPad with teleconferencing capability 2. Schedule appointment/visitation time with patient and/or family and notify bedside nurse 3. Contact patient and/or family to train them in videoconferencing technology 4. Send link or ID number to videoconference to all invited participants (patient, family, and/or professionals from other teams) 5. Arrive into video visit for provider pre-meeting and technological time-out to anticipate technological issues 6. Patient and/or family join video visit | Empirical only | Training or education in palliative care | Provider time; personal protective equipment | Yes | Yes | Telepalliative skills without nonverbal techniques are novel and take time to develop, and is a powerful tool that can promote good communication and high-quality care. | 6 |
| Dhala, A. | Patients, families | Virtual Intensive Care Unit (vICU) | 1. The vICU infrastructure provided a readily available and much more accessible means of connecting the patients with COVID-19 with their families 2. Offered this technology to the families for emotional support and improved patient care 3. Two vRNs (in the operation center) were tasked with reaching out to the bedside teams and collaborating with the bedside nurses, physicians, and unit managers to gain access to the patients’ families 4. Family members received links on their smartphones that instantly connected them with their loved one’s ICU room using the Consultant Bridge feature 5. The vICU patients logged approximately 20 to 40 calls per day using the Consultant Bridge during this period 6. Because restrictions on visitors also applied to non–COVID-19 patients and their families, the Consultant Bridge was used for all ICU patients 7. The results of a short postcall quality assessment survey showed overwhelming satisfaction with the access to the patient using vICU technology 8. Similar to regular visitations, we used the Consultant Bridge feature of vICU to enable palliative care | Empirical and theory | Not reported | Provider time; education and training | Yes | Yes | Virtual family visitation via the Consultant Bridge application, palliative care delivery, and specialist consultation for patients with COVID-19 exemplify the successful adaptation of the vICU implementation. | 5 |
| Feinstein, R.E. | Professionals | Implementation of the 'Healthcare Worker Mental Health COVID‐19 Hotline' | 1. Anticipate mental health needs 2. Use leadership capable of mobilizing the systems and resources 3. Convene a multidisciplinary team 4. Delegate tasks and set timelines 5. Choose a clinical service model 6. Motivate staff as a workforce of volunteers 7. Develop training and educational materials 8. Develop personal, local, and national resources 9. Develop marketing plans 10. Deliver the training 11. Launch a 24 hr/7days per week Healthcare Worker Mental Health COVID‐19 Hotline, and launch follow‐up sessions for staff 12. Structure data collection to determine effectiveness and outcomes 13. Obtain funding (not required) | Empirical and theory | Social workers and psychological therapists | Not reported | Yes | Yes | A coherent approach to developing a COVID‐19 Mental Health Hotline was helpful and led to success. | 5 |
| Kuntz, J.G. | Patients, families, professionals | Standard workflow enabling e-family meetings | 1. Identify a single point of contact for the family and schedule the meeting 2. Provide meeting link and instructions in email to family 3. Plan entry, 'donning' and positioning of the tablet device 4. Start the E-Family Meeting 5. Conducting the e-family meeting 6. Offer a virtual visit 7. Ending the meeting 8. Recover, 'doff,' and clean the tablet and stand | Empirical and theory | Not reported | Technology access; hearing and sight disabilities; inability to support families longitudinally after the video session | Yes | Yes | Telemedicine for e-family meetings has strengthened the relationships between critical care and palliative care across our system particularly in the COVID-19- cohorted intensive care units. | 5 |
| Landa-Ramirez, E. | Families, professionals | Intensive preparatory clinical actions in the emergency psychology department | 1. Online death notification education; an online course with the aim of training health professionals in providing death notifications 2. Remote crisis notification by telephone; to carry out a remote crisis intervention, eight psychologists from the emergency psychology department were trained to connect with the person, focus the call, relieve any emotional stress, promote coping strategies, and decide on the next steps 3. Support for health care professionals; voluntary psychological online assessments to identify the emotional state of health care staff 4. Information and relaxation techniques for emotional regulation, problem-solving techniques, stigma management, adjustment of negative thoughts, social support, and teamwork | Empirical and theory | Training or education in psychology | Not reported | Yes | Yes | It is important the psychology concerning the transmission of death notifications in the COVID-19 context. | 5 |
| Arya, A. | Professionals | Multipronged approach that is focused on 'stuff, staff, space, systems, sedation, separation, communication, and equity' to provide comprehensive palliative care | 1. Stuff • Stockpile comfort medications (morphine, haloperidol, midazolam and scopolamine) or symptom management kits, especially in long- term care and community settings • Suspend regulations that limit the availability and prescription of injectable morphine and hydromorphone • Stockpile equipment to deliver medications, including subcutaneous cannulae and delivery equipment (e.g., pumps or syringe drivers) • Stockpile personal protective equipment for palliative care professionals in long-term care and community settings 2. Staff • Identify and mobilize all clinicians with palliative care experience • Provide brief education for front-line professionals on symptom management for acute respiratory illness, emphasizing the safety of symptom-targeted opioids as an early option • Engage allied health to provide emotional support to patients, and grief and bereavement support to family members 3. Space • Optimize the use of beds in hospice and palliative care units, particularly for patients who do not have COVID-19, via direct admission from the emergency department or community • Identify separate wards and nonclinical areas in acute settings that might be appropriate for those expected to die — essentially palliative care units for patients with COVID-19 4. Systems • Adopt a triage system to determine which patients require specialist palliative care consultation and which patients can be seen virtually • Maximize the use of telemedicine, both for efficiency and reducing infection • Develop standardized order sets for acute, long-term care and community settings • Form palliative care provider “groups” that can provide mutual support and coverage if a provider becomes sick or is overwhelmed 5. Sedation • Be prepared to use palliative sedation for symptoms that are refractory to common comfort medications 6. Separation • Enable video calling to connect patients with family members who are separated because of travel and visitor restrictions 7. Communication • People with frailty or comorbid illness should update their advance care plans and indicate if they wish to avoid transfers to hospital or critical care in the event of serious illness • Before the expected surge in patients, professionals should review treatment plans when patients with advanced cancer, end-stage organ failure, frailty or dementia are requesting life support or cardiopulmonary resuscitation. These patients are unlikely to survive and recover after an admission to an intensive care unit. 8. Equity • Palliative care professionals should pay greater attention to patients who are marginalized. When the health care system is strained, systemic inequity will worsen. • Protocols for critical care triage may be implemented. Patients who are denied critical care should be the top priority for palliative care. All patients must be cared for. | Empirical only | Training or education in palliative care | Not reported | Yes | Yes | A multipronged approach can guide planning and ensure that the palliative care needs of patients and their family members are met. | 5 |
| Belli, L.F. | Families, professionals | Four 'moments' to guide the transmission of bad news at a distance | 1. At first, when making contact with the potential recipient of the news, it is necessary to clearly identify (mention name, surname and institution from which the call) and briefly explain the reason for the call which allows the person who is try on the other side to anticipate that this is important news 2. In a second moment of communication, it is important never lose sight of the fact that the communication of bad news by telephone is strictly verbal 3. In a third moment, after the presentation, you must explicitly anticipate that the reason for the call is communicate bad news 4. The fourth moment corresponds to the end of communication, must be accompanied by a space for responder doubts or questions that the person may have | Empirical only | Not reported | Resource limitations | Yes | Yes | A comprehensive and structured approach reduces negative impact that difficult conversations have on health professionals. | 5 |
| Benaque, A. | Patients, families, professionals | Healthcare contingency plan | 1. Safety: preserve safety of people, including professionals, people we care for, and indirectly also all society 2. Continuity of care: guarantee the continuity of care with the will to serve people, covering the needs of our patients and their families adapting our holistic care model process to the new reality 3. Ethical responsibility: once the previous two goals are fulfilled, we should attempt to go further and add positive value to what we already did before, living up to our ethical responsibility with the community 4. Importance of visibility and communication of all these changes: posted regular press releases and in social networks and maintained close communication with families of Day Care Unit users 5. The distribution of support and health education material was also prioritized as well promoting the position of Alzheimer’s Europe in defense of the rights of people with dementia, endangered in this crisis times | Empirical only | Not reported | Familial reluctancy | Yes | Yes | The Fundacio ́ ACE model of care adapted to the COVID-19 pandemic helped to guarantee the continuity of care while preserving the safety of patients, families, and professionals. | 5 |
| Fang, J. | Patients, families | Factors and features to consider for videoconferencing applications | 1. Security 2. Patient privacy 3. Usability  4. Administrative overhead 5. User interface | Empirical only | Not reported | Technology access; unfamiliar applications and platforms; provider time; education and training | Yes | Yes | Expansion of in-hospital telehealth capabilities requires adapting existing systems based on these design principles for individual hospitals. | 5 |
| Fusi-Schmidhauser, T. | Families | Tailored management plan for patients with COVID-19 who are not suitable for mechanical ventilation | 1. Patients were identified as being stable, unstable, or at the end of life using the early warning parameters for COVID-19 2. A COVID-19 specific assessment tool was developed locally, focusing on key symptoms observed in this population which assess dyspnoea, distress, and discomfort 3. This tool helped to guide the palliative care management as per patients’ disease stage 4. Family members are incorporated/considered in the unstable and end-of-life phases | Empirical only | Not reported | Personal protective equipment | Yes | Yes | Palliative care should be at the forefront to help make the best decisions, give care to families, and offer spiritual support. Nursing interventions and family involvement were adapted as per patients’ disease stage and infection control requirements. | 5 |
| Griffin, K.M. | Families, professionals | Communicating with families and facilitating staff wellness | 1. A limited number of patient surrogates are designated to communicate with physicians 2. Each family member is offered a second supportive call from a palliative care team member 3. The palliative care team discusses cases with each COVID-19 unit daily to assess for participating surrogates 4. In the setting of imminent death or immediately after death, a single family member in PPE is provided an opportunity to visit 5. If visitation is impossible, the staff can creatively use video conferencing to allow loved ones to visualize and speak to their dying family member 2. Engage mental healthcare professionals with expertise in the management of trauma and acute stress to offer individual and group support to staff | Empirical only | Training or education in psychology and palliative care | Not reported | Yes | Yes | A program that provides access to palliative care and ethics consultants, 24 hours a day, has helped facilitate conversations around end-of-life. | 5 |
| Lissoni, B. | Families, professionals | Psychological interventions for intensive care unit clinicians and family members | Interventions for staff- 1. The need for physical safety to be able to work with a sense of individual and collective self-efficacy 2. The need for belonging, that is, to feel a part of a cohesive team with shared working objectives 3. The need to be listened to 4. The need for decontamination and physical and emotional decompression.  Interventions for family member- 1. The need to receive information. Information that is provided correctly helps the family to give meaning to a situation that is out of control 2. The need for reassurance on the fact that their loved one is not abandoned and he or she is being taken care of not only from a clinical point of view but also relationally 3. The need to be listened to and emotionally supported 4. The need to preserve a relationship with their loved one 5. The need to be supported in the initial process of grief | Empirical only | Training or education in psychology | Not reported | Yes | Yes | The objective of psychological interventions in the acute phase is to sustain resources and reinforce the factors that may provide a protective action from posttraumatic stress and complicated grief. | 5 |
| Maves, R.C. | Patients, families, professionals | Operational steps to implement a triage system | 1. A hotline and electronic communication platform may help gauge community response and concerns during the event and will inform leaders regarding the emotional reassurances needed by members of the society 2. Include the use of technologic solutions to attempt to preserve as much intrapersonal contact as possible 3. Ancillary team members will need to assist with the burden of communication with ICU families 4. These professionals should remain in regular communication with family via telephone, text message, or other means as best possible 5. It is additionally important to anticipate and plan for supporting these professionals, who themselves will require support because of secondary moral distress 6. Existing resilience measures and support programs will need to be strengthened to protect the psychological well-being of patients, families, and the HCWs treating them both during and after these events 7. Work to preserve the integrity of family units, especially in cases of young children and during end-of-life 8. Ensure support for grieving families | Empirical only | Training in institutional social work, mental health, and palliative care | Not reported | Yes | Yes | Establishing the infrastructure necessary helped to equitably meet the clinical needs of the greatest number of patients with COVID-19 during a time of scarce resources. | 5 |
| Morley, G. | Families, professionals | Evidence-based recommendations for leaders to address moral distress | 1. See and seek moral distress • Look for ethical concerns and signs of moral distress • Inquire and consider whether an Ethics Consultation is indicated 2. Understand moral distress • Understand through active listening • Be receptive to diverse perspectives • Model a self-reflective process: be aware of your own biases, remember that ethical issues often are not black and white, and avoid responding with correction/rebuke 3. Pay attention and assess workplace climate • Acknowledge ethical challenges and moral distress • Assess the unit climate, culture, tone • Work to mitigate power differentials between caregivers • Explore and note repeated occurrences and problems • Assess professional risks of speaking up 4. Promote a receptive environment and engage team members • Encourage and create spaces for moral dialogue • Encourage and role-model respectful communication across disciplines • Promote team-based dialogue and discussion when ethical issues arise 5. Open opportunities for dialogue • Encourage debriefing • Ask whether members of the team might benefit from further discussion with an ethics expert: consider whether a Moral Distress Reflective Dialogue or Debrief is indicated • Utilize resources: bring team members to multidisciplinary meetings, invite bedside nurses to family meetings, and participate in Bioethics rounds 6. Reflect, evaluate, and revise • Establish self-care as a custom, ask team members how they are doing, and explore whether they need any additional support 7. Transform negative environments • Acknowledge that the environment is changing, be transparent and ready to answer questions | Empirical only | Strong leadership capabilities | Not reported | Yes | Yes | Leaders play an important role in providing support when moral distress is experienced by their teams and moral distress is more likely to occur in work environments that are perceived to have a poor ethical climate. | 5 |
| Ritchey, K. C. | Patients, families, professionals | Formal standard operating procedure for tablet use for inpatient consultations called 'Humanizing Technology for Communication Near the End of Life' | Elements- 1. Prepare family prior to the conference with what they may see 2. Provide regular reassurance and check-ins 3. Discuss the 'pitfalls of technology' 4. Consider elements of physical and human contact with front line staff  Steps- 1. If patient is sedated and unconscious, describe the comfort measures provided and how symptoms are being addressed 2. If the patient is on a mechanical ventilator, describe how that will look before starting the visit 3. Name everyone in the room and how they are connected to the patient 4. Guide the family on what things to say Check-in on emotions 5. Explain how the patient is being monitored 6. Set expectations about quality of video/sound/ etc. 7. Have a back-up plan if video connection fails 8. Name and address frustrations which may develop due to the use of technology 9. Consider a test call with family prior to visit to make sure technology works on both ends 10. Sing favorite songs 11. Have family watch elements of gentle/comfort-related care such as moistening lips, holding patient’s hand, etc. 12. Have the family share favorite stories, reminisce about the patient, describe the legacy the patient has created | Empirical only | Not reported | Single case study | Yes | Yes | Conceptualizing a formal standard operating procedure for tablet use as a business model innovation and identifying the processes necessary to deliver personalized virtual palliative care within a quality improvement framework was a useful template for reinventing palliative care service delivery. | 5 |
| Schoenherr, L.A. | Families | Identification of palliative care needs among patients with COVID-19 | 1. Patients were screened through a combination of chart review and brief provider interview 2. Palliative care consultations were provided via telemedicine for those with unmet needs identified | Empirical only | Training or education in palliative care | Not reported | Yes | Yes | Emotional, spiritual, and existential supports provided by our social worker, chaplain, and clinical nurse specialist, worked to mitigate trauma and complicated grief and formed trusting longitudinal relationships with patients and families during the course of prolonged hospitalizations. | 5 |
| Viaux, S. | Patients | Postpartum telephone interview with a psychologist | 1. Interviews were conducted at days 10–12 postpartum, plus another one 6–8 weeks later 2. Standardized interviews of approximately 30 min comprise a free exchange followed by a psychological assessment using questionnaires validated during the perinatal period 3. In the first interview, the conditions of discharge home are discussed and the mother's experience of childbirth is discussed and the mother's experience of childbirth is evaluated by means of the perinatal post-traumatic stress disorder questionnaire 4. The following are also evaluated during the two interviews: the Mother-Infant Bonding Scale, the Dyadic Adjustment Scale, and the Edinburgh Postnatal Depression Scale, using a score >12 as the threshold defining increased risk of postpartum depression, so as to propose suitable psychological or psychiatric support | Empirical only | Psychiatrist | Not reported | Yes | Yes | The organization implemented to reduce the risk of psychological vulnerability was successful in postpartum women, and should be extended to personnel who provide care at childbirth. | 5 |
| Wallace, C.L. | Families, professionals | Core pillars for professionals navigating grief | 1. Communication 2. Advance care planning 3. Self-care practices | Empirical only | Training or education in palliative care | Not reported | Yes | Yes | Palliative care professionals are well-positioned to serve as a resource to their colleagues in other specialties based on their training and expertise in working with patients near end-of-life. | 5 |
| Cho, H.J. | Patients | Choosing Wisely® partnered with patients and clinician societies to develop a Top 5 recommendations list for eliminating unnecessary testing and treatment | 1. Do not obtain nonurgent labs in separate blood draws if they can be batched together 2. Do not use bronchodilators unless there is active obstructive airway disease, and if needed, use metered dose inhalers instead of nebulizers 3. Do not use posterioranterior and lateral chest x-ray as initial imaging; use a portable chest x-ray instead 4. Avoid in-person evaluations in favor of virtual communication unless necessary 5. Do not delay goals of care conversations for hospitalized patients who are unlikely to benefit from life-sustaining treatments | Empirical and theory | Not reported | Not reported | Yes | Yes | Critical thinking to evaluate the potential harms to extended healthcare teams and to strive further to eliminate overuse from care is required. | 4 |
| Hart, J.L. | Families | Framework for barriers to family-centered care and toolbox of strategies to implement in the inpatient setting | Barrier- 1. Family spokesperson or health-care proxy unavailable during daytime hours 2. Family members without internet access or device capable of videoconferencing 3. Patient without device capable of videoconferencing 4. Family members do not speak the same primary language as clinical team 5. Family members or patient have limited technological literacy 6. Patient lacks communication aids such as glasses or hearing aids  Strategies- 1. Engagement of families with patients: synchronous communication 2. Engagement of families with patients: asynchronous communication 3. Engagement of families with patients: environment 4. Communication between clinical team and family | Empirical and theory | Not reported | Not reported | Yes | Yes | Innovative approaches that involve family members in inpatient care during the COVID-19 pandemic may lead to long-lasting progress in, rather than regression from, the standards of family-centered care the health-care community has recently achieved. | 4 |
| Knights, D. | Patients, families, professionals | Providing care under research limited settings | 1. Integrate palliative care into everyday practice • Support generalist colleagues to ensure the goal of care is reduction in suffering, not purely survival • Consider innovative methods of communicating with and supporting families, such as virtual hubs • Consider provision of physical ‘care bundles’ to support patients and reduce psychological distress of staff • Increase understanding and provision of advance care plans, including decisions around ceilings of treatment 2. Simplify biomedical management and multidisciplinary team work • Ensure easy access to tailored, contemporary clinical guidance and decision aids, including responses to medication and equipment shortages • Upskill generalist staff in holistic exploration of needs, symptom assessment and communication skills • Consider where task shifting or link nurse models may improve care 3. Use volunteers effectively • Consider creative integration of volunteers, third sector organisations and palliative services • Provide training, guidance and support for psychological support and befriending volunteers • Enhance coverage of care and family support; widen the community sense of accountability | Empirical and theory | Not reported | Not reported | Yes | Yes | Solutions to providing palliative care in resource limited settings should harness learning together to support each other and innovation in the face of adversity. | 4 |
| Wei, E. | Families, professionals | Reinventing patient and staff support | Keeping patients connecting with their families- 1. Daily phone calls and video visits 2. Support for end-of-life decisions and mourning  Supporting staff and their families- 1. Resources to address emotional and psychological trauma 2. Temporary lodging 3. Child care 4. New rituals to celebrate recovery 5. Coping with staff deaths 6. Complimentary meals, transportation, and uniforms | Empirical and theory | Not reported | Not reported | Yes | Yes | Health care workers who experience emotional and psychological trauma often try to suppress it, and the tradition of stoicism in the practice of medicine and the stigma often associated with behavioral health conditions prevent many health care workers from asking for help. | 4 |
| Fausto, J. | Patients, families | Palliative care inpatient response plan | 1. Identifying and addressing goals of care 2. Addressing code status to reduce the risk of unwanted or nonbeneficial cardiopulmonary resuscitation in the context of COVID-19 3. Identifying and addressing moderate or severe symptoms not adequately addressed through primary palliative care 4. Supporting family members in the difficult context of restricted visitation and possible self-quarantine | Empirical and theory | Not reported | May not be generalizable; undergoing constant modifications; has not been implemented | Yes | No | It is important that we share planning and experiences with each other to minimize the amount of unnecessary work in developing, adapting, and implementing strategies. | 4 |
| Hall, D.E. | Patients | Pastoral care for dying patients | 1. Pastoral care should be available for both those with and without active infection 2. Many people remain in hospital for reasons other than COVID-19, and there is little risk in visiting these people 3. More caution will be needed to visit the infected, but like all essential personnel admitted to health care facilities, local clergy should be screened for infection and trained in the proper use of personal protective equipment 4. Hospital administrators should work with local religious leaders to develop safe techniques for prayer, laying on of hands, anointing with oil and last rites | Empirical and theory | Training or education in palliative care | Personal protective equipment | No | No | In his letter to faithful Christians in Rome, St. Paul reassures them that neither death, nor life, nor powers, nor principalities, nor things present nor things to come, nor height, nor depth—nothing, not even COVID-19—can separate us from the love of God in Christ Jesus, whose hands and feet are made up by his people on earth called the Church. | 4 |
| British Red Cross | Patients, families | Involving carers and family despite visitation restrictions | 1. Family and carers being provided with a single point of contact who they can get in touch with for information about their loved one while they are in hospital 2. Guidance ensuring people admitted to hospital are asked if they have a carer who should be involved in decision-making about their care 3. If the patient does name a carer, attempts to contact and involve them in discussions about hospital care and discharge should be made at every step of the process 4. Carers themselves receiving a wellbeing check, being told how to access additional community services to support them after their friend or relative's discharge and making them aware of their right to a carer’s assessment from their local authority 5. Hospital discharge teams checking what support carers can provide and whether they have a contingency plan should their circumstances change 6. If they are not able to provide their usual level of support to the patient, this needs to be considered | Empirical only | Not reported | Not reported | Yes | Yes | Hospitals should give families and carers all recommendations and information so they have a point of contact for the follow-up support of their loved ones or clients, and national policy should be updated requiring hospitals to do this. | 4 |
| Canadian Foundation for Healthcare Improvement | Patients, families, professionals | Reintegrating family caregivers as essential partners in care | 1. Ensure a foundation of patient- and family-partnered care 2. Revisit policies on family presence with patient, family and caregiver partners at the table 3. Distinguish between family caregivers who are essential partners in care and visitors 4. Consider the needs of people who face specific risks without the presence of family caregivers as essential partners in care 5. Take a comprehensive, balanced approach to assessing risks 6. Establish a rapid appeal process 7. Increase the evidence to guide decisions regarding family caregiver presence | Empirical only | Not reported | Not reported | Yes | Yes | It is important to regularly examine and re-consider restrictive guidelines to ensure a comprehensive approach that takes into account the full range of risks from a patient and societal perspective. | 4 |
| Cleveland Clinic | Patients, families | Development of an 'Enterprise Virtual Visit' team to connect patients with their loved ones | 1. Less than 2 weeks after Cleveland Clinic’s visitation restrictions were put in place, the team deployed approximately 600 shared iPad devices to 363 nursing units across 20 hospital locations in Northeast Ohio and Florida 2. Units received up to four devices depending on unit size and number of inpatient beds 3. To virtually connect patients with their families, the team established a process for scheduling virtual visits 4. Nurses collect family contact information from patients and send it to the Office of Patient Experience 5. Members from the office then call each family directly to ensure family members have a device they can use for connecting, as well as an account with the appropriate mobile app 6. They coordinate an ideal time for the nurse and patient to virtually connect with the family and then schedule a virtual visit 7. With assistance from the bedside nurse, patients use mobile devices and virtual apps to visit with their families during the scheduled time | Empirical only | Not reported | Not reported | Yes | Yes | Providing the ability to remain connected in isolation and quarantine has reduced feelings of separation and loneliness, while aiding in the healing process. | 4 |
| Cohen, S.P. | Patients, professionals | Framework of factors for pain practitioners and institutions to balance goals of risk mitigation for health care professionals, risk mitigation for patients, conservation of resources, and access to pain management service | 1. Acuity 2. Co-morbid psychiatric (e.g. severe pain-related depression) and social (e.g. single mother of young children with limited resources) considerations 3. Pain level and accompanying functional impairment 4. Likelihood of the visit/ procedure providing meaningful benefit 5. Likelihood of the patient to seek scarce emergency services, or be started on opioids 6. Need for physical examination 7. Risk association with in-person visit or procedure 8. Work status (e.g. is the patient currently working or likely to return to work with adequate pain treatment? 9. Job (i.e. prioritizing first-responders will provide the greatest benefit for society) | Empirical only | Not reported | Not reported | Yes | Yes | Weighing the dynamic balance between access to pain care, which can have long-term personal and socioeconomic benefits, with the immediate goal of minimizing exposure risk for frontline healthcare professionals and vulnerable patients is required. | 4 |
| Devlin, J.W. | Patients, families | Society of Critical Care Medicine’s 'ABCDEF' safety bundle framework; F—Family Engagement and Empowerment | 1. For patients who are wakeful, daily telephone, facetime, or zoom communications with family is encouraged 2. Families should be encouraged to provide the ICU with family photos and provide clinicians with the E-stories and music the patient enjoys | Empirical only | Not reported | Not reported | Yes | Yes | Reemploying the use of evidence-based strategies that are appropriately adapted for use in the COVID-19 pandemic may be one of the best mechanisms by which to increase ventilator and ICU capacity and help critically ill adults with COVID-19 transition toward recovery and survivorship. | 4 |
| Frampton, S. | Patients, families, professionals | Planetree International and The Pioneer Network convened an international, multistakeholder coalition including patient, family, and elder advocates along with experts in quality, safety, and infectious disease to develop a new set of 'Person-Centered Guidelines for Preserving Family Presence During Challenging Times' | 1. Assess and continually reassess whether there is a need for restrictions based on cur- rent factual evidence 2. Minimize risk of physical presence by following appropriate infection control guidelines 3. Communicate proactively so families do not appear at a facility unaware of restrictions 4. Clearly state compassionate exceptions to restrictions 5. Minimize isolation in cases where family is unable to be physically present 6. Use a shared decision-making approach to communicate risks and benefits in cases where family can be physically present 7. Enlist family as members of the care team who share in the responsibility for abiding by established safety protocols 8. Enhance discharge education and follow-up to support successful transitions of care | Empirical only | Not reported | Not reported | Yes | Yes | Family presence can be practiced during the COVID-19 outbreak with well-designed guidelines that delineate clear, safe, and humane approaches that promote shared decision-making and respect the rights of all groups involved. | 4 |
| Holland, D.E. | Families | Guidelines related to grief and loss | 1. Assist families to identify the people they depend on during difficult times or ask, “Who are your go-to-people?” Then encourage them to connect with these people (family members and/or friends) by phone, Zoom video visits, social media, or in-person visits 2. Suggest family, friends, and loved ones express care in ways that do not involve personal interactions 3. Designate a family member who can help coordinate these activities when the family caregiver may not have the energy or desire to do so 4. Counsel families to utilize resources provided by mental health professionals 5. Encourage use of postmortem hospice services including bereavement services 6. Consider an immediate announcement of death and another announcement closer to the funeral/ memorial in order to begin closure and to gain more social support 7. Encourage the family to take part in an activity that has significance to them and the loved one they lost, such as planting flowers or a tree or preparing a favorite meal, in memory of the loved one | Empirical only | Not reported | Not reported | Yes | Yes | Clearly communicating up-to-date information and helping to set family expectations about the death and dying process during the COVID-19 pandemic helped to reduce risks for transmitting the virus within communities while supporting families. | 4 |
| Mistraletti, G. | Patients, families, professionals | Five essential aims to enable healthcare team to provide by telephone or video calls an optimal level of communication with patient’s relatives under circumstances of complete isolation | Components of effective communication- 1. Truthfulness 2. Consistency 3. Gradualness  Essential aims of effective communication- 1. Give understandable information about the disease and treatment options 2. Obtain information on the relatives’ expectations and the patient’s values and choices 3. Show empathy and participation 4. Allow relatives to express their emotions 5. Prevent misunderstandings and conflicts with the care team | Empirical only | Not reported | Not reported | Yes | Yes | A well-conducted phone call by the doctor on the clinical conditions can be a tool to care for those who cannot personally see their loved one, and to ease the sense of guilt linked to the feeling of abandoning his/her own loved one. | 4 |
| Selman, L.E. | Families, professionals | Recommendations for bereavement support | 1. Advance care planning 2. Proactive, sensitive, and regular communication with family members alongside accurate information provision 3. Enabling family members to say goodbye in person where possible 4. Supporting virtual communication 5. Providing excellent symptom management and emotional and spiritual support 6. Providing and/or sign-posting to bereavement services 7. An organizational and systemic approach which includes access to informal and professional support | Empirical only | Training or education in palliative care | Provider time | No | No | Understanding the risk of trauma and moral injury to staff in the current pandemic is essential for the early identification and prevention of harm. | 4 |
| Wynne, K.J. | Patients | Ethical principles to guide palliative care and symptom control in humanitarian contexts | 1. Inclusiveness 2. Communication 3. Transparency 4. Accountability 5. Consistency 6. Ensuring comfort | Empirical only | Training or education in palliative care | Limited literature to inform | No | No | WHO guidance on the integration of palliative care and symptom relief in humanitarian response is and will continue to be key to enabling what is a moral imperative; immediate steps to support better resolutions to ethical dilemmas of the provision of palliative care will require honest debate, concerted research effort, and international, national and local ethical guidance. | 4 |
| Abrams, E.M. | Patients, families, professionals | Pandemic shared decision making needs assessment | 1. Defining a shared decision making process 2. Developing a certification process for decision aids and providing incentives for their evaluation and maintenance 3. Promoting competency in shared decision making 4. Developing pandemic-specific shared decision making measures 5. Fostering a culture of shared decision making 6. Using shared decision making to help with documentation of quality improvement | Theory only | Not reported | Inability to support families longitudinally after the video session | Yes | Yes | Within an organizational framework the incorporation of shared decision making principles has the potential to modify health care and service allocation and reduce costs. | 4 |
| Brucato, A. | Patients, families | Communicating with patients and family caregivers | 1. I always try to put myself in the shoes of the person I am talking with, whether they are the patients themselves or their caregivers, as this helps me understand and empathize with their perspective and also to anticipate their feelings and response 2. I always consider the possibility that what I’m communicating might be interpreted very differently by my patients or their caregivers, albeit in good faith 3. While miscommunication is always problematic in itself, its outcomes can be quite serious in relation to disease prognosis, or to impending death 4. I believe it is very important to communicate clearly and explain, and then to explain again | Theory only | Training and experience in communication strategies | Not reported | Yes | Yes | It is important to include sessions on how to communicate with patients, and particularly with families and caregivers in the early phases of response planning for crises and emergencies. | 4 |
| Lai, L. | Professionals | Progressive web application called 'PalliCOVID' on palliative care | 1. Accurate: Content was reviewed by palliative care experts to reflect the best available scientific evidence 2. Practical: Recommendations were designed to be useful and implementable by nonpalliative care clinicians in a variety of care settings 3. Accessible: Content was presented in a format that was optimized for viewing on both mobile devices and desktop computer screens 4. Applicable: Content was specific to the care of patients with confirmed or suspected COVID-19 infection and took into account the need to limit face-to-face interactions because of enhanced infection- control measures and restricted visitor policies | Theory only | Not reported | Lack of information on user behaviour | Yes | Yes | Future dissemination strategies should specifically target nurses or have product design specifications to specific user populations; different roles such as nursing spend the majority of their time at patients’ bedsides and are more likely to be aware of patients’ specific symptoms and end-of-life needs than perhaps physicians or advanced practice professionals. | 4 |
| Apoeso, O. | Patients, families, professionals | Workflow from an extra geriatric attending, a fellow, and a nurse practitioner added to help care for patients at end of life | 1. Patient selection and triaging in collaboration with palliative care consult teams, 24 hour palliative care support line, emergency department, and referrals from community and other facilities 2. Bedside clinical care 3. Collaboration with interdisciplinary team and nursing staff to coordinate video/audio and physical visits and daily clinical updates for families | Unspecified | Training or education in palliative care | Not reported | Yes | Yes | A stand-alone hospital unit to provide end-of-life care promoted optimal physical and psychological symptom management, increased family satisfaction, and facilitated resource allocation. | 4 |
| Fan, P.E.M. | Patients, families | Bedside telephone and routine calls from management | 1. A bedside telephone is available in each isolation room 2. If they are unable to use the phone themselves, the nurse could dial the number for them to reach their families using the phone in the room 3. Senior Patient Experience Managers contact all patients admitted to the isolation wards on a daily basis 4. If the patient is unable to answer the call (for instance, if they do not have a personal phone or is not cognitively able to carry out a conversation), the patient's next of kin will be contacted instead 5. The purpose of the call is to provide support to the patient or their family; detect possible signs of distress and to find out if there are any concerns about hospitalization that may be addressed in a timely manner | Unspecified | Not reported | Telephone calls do not have visuals | Yes | Yes | The phone in the room and daily phone calls has enabled conversations between patients and families, and allayed their anxieties. | 4 |
| Munch, U. | Patients, families, professionals | Recommendations for the support of suffering, severely ill, dying or grieving persons | 1. Palliative care patients should be exempted from any ban on visitors 2. Families should be able to visit dying patients even on intensive care units or isolation wards, using adequate protective equipment 3. Alternative options, such as video telephone calls or via social media should be explored for patients in isolation 4. Families should also be enabled to say goodbye to the deceased with adequate protective equipment or should be offered alternative real or virtual options for remembrance and commemoration 5. Health care professionals coping with the exceptional stress should be continuously supported | Empirical and theory | Training or education in psychology | Not reported | No | No | Recommendations for the support of suffering, severely ill, dying or grieving persons require clear communication and leadership structures, communication training, psychosocial support, and framework conditions for clinical work. | 3 |
| Cherepanov, E. | Professionals | Peer support and camaraderie | Do- 1. Check in on each other and be aware of those who are in distress 2. Random acts of kindness, small compliments, and favors can go a long way 3. Be available when someone wants to talk 4. Be clear with setting time limits, and do not send mixed signals 5. Accept that one can remain distressed even after talking to peers 6. Respect the individual’s way of dealing with difficulties 7. Give feedback if stress affects the job performance or makes the provider unsafe  Don’t- 1. Don’t assume the role of psychotherapist 2. Avoid emotional incongruency, like being Pollyannaish 3. Don’t try to fix others or solve their life problems 4. Don’t suggest they relax 5. Don’t suggest that talking about feelings is going to make them feel better 6. Don’t tell a peer not to think about work at home or about home at work 7. Don’t neglect practical assistance when possible; offering to help with small tasks can go a long way 8. Avoid labeling peers’ behavior or feelings as pathological 9. Don’t forget that you also need supports | Empirical only | Not reported | Not intended to substitute of professional mental health support | No | No | Introducing a self-awareness framework prioritizes the awareness of the available choices and making situation-appropriate and informed decisions about balancing one’s own and others’ needs. | 3 |
| Chua, I.S. | Patients, families, professionals | Key elements of webside manner when conducting serious illness conversations by virtual visit | 1. Proper set up 2. Acquainting the participant 2. Maintaining conversation rhythm 4. Responding to emotion 5. Closing the visit 6. Technical difficulties 7. Lack of prerequisite technology to conduct virtual visits 8. Patients who are too ill to participate 9. Patients who find virtual visits too technically challenging | Empirical only | Not reported | Technology access; familial reluctancy; foreign languages | No | No | Effective webside manner skills are essential to help clinicians maintain their empathic connection as they virtually guide patients and families. | 3 |
| Gupta, A. | Families | Ensuring completion of advanced care directives | 1. Increased patient access to electronic medical records where they can document end-of-life wishes 2. Increase the frequency with which patients are requested to complete advanced care directives to increase patient uptake 3. Lack of patient familiarity with advanced care directives can be addressed through awareness and educational campaigns 4. To rectify advanced care directive implementation inequalities for racial and ethnic minorities, healthcare professionals must first be aware that they exist 5. Small-scale advanced care directive educational efforts will be exponentially more effective in a system that protects vulnerable populations and produces well-trained palliative care professionals | Empirical only | Training or education in palliative care | Not reported | No | No | As patients frequently opt-out of aggressive treatments, widespread adoption of advanced care directives may decrease competition for strained resources during the COVID-19 pandemic. | 3 |
| Horsch, A. | Professionals | Strategies to enhance provider morale | 1. Managers should ensure that time and space is given to help staff reflect on and make sense of the morally difficult decisions they must take 2. One such approach may be Schwartz rounds organised by team leaders, which could also be carried out in a virtual format 3. Schwartz rounds follow a structured format that allows healthcare professionals to discuss and reflect on the emotional work-related challenges of their day-to-day practice, in a safe and confidential space 4. A peer support programme that is available to all staff could be offered, including a discussion about moral injury and early warning signs to look out for 5. Staff reporting high and persistent levels of psychological distress or mental health problems should be identified early and offered appropriate specialist support | Empirical only | Strong leadership capabilities | Not reported | No | No | Health services should begin offering psychosocial support for staff to protect their mental wellbeing if they are to continue to provide high quality care. | 3 |
| McKee, M. | Patients, professionals | Overcoming barriers to care for deaf and hard of hearing patients during COVID-19 | 1. Clear face masks; these allow disabled patients or family members to lip-read the health care worker 2. Remote interpreters; remote interpreting can be supplied by existing hospital staff interpreters, contracted community-based medical interpreters, or video remote interpreting agencies 3. Captioning apps; these provide another in-person communication tool and be used either on the patients’ personal or institution’s devices 4. Virtual visits; videoconference platforms allow a third-party to provide closed captioning 5. Signage; signs can be printed with hearing loss icons or text and posted in highly visible areas in the patient rooms 6. Communication boards; to permit quick communication between clinicians and staff when other accommodations are unable to be arranged | Empirical only | Foreign language interpreter | Not reported | No | No | Deaf and hard of hearing patients have additional barriers added, and require effective clinician-patient communication. | 3 |
| Dudzinski, D.M. | Patients, families, professionals | Lessons/advice related to 'Ethics Lessons From Seattle’s Early Experience With COVID-19' | 1. Anticipate that healthcare workers will feel that they are abandoning patients when implementing restrictive visitation policies 2. Address those feelings at the same time you are announcing the changes 3. Innovative palliative care professionals seek donations for video baby monitors via Facebook | Theory only | Not reported | Not reported | Yes | Yes | Early physical distancing measures seem to be working; interdependence, generosity, kindness, and resilience are where hope lies. | 3 |
| Morris, S.E. | Families | Categories for caring for families before the patient's death to facilitate post-loss adjustment | 1. Communication skills (before and after) 2. Care processes (before and after) 3. Tools to promote connection (before only) | Theory only | Not reported | Not reported | Yes | Yes | There is an urgency from a public health perspective, to expand bereavement services in an attempt to mitigate poor bereavement outcomes, including prolonged grief disorder and other psychiatric disorders. | 3 |
| Atreya, S. | Patients | Teleconsultation for triaging | Not reported | Unspecified | Not reported | Single care center | Yes | Yes | Telehealth service was proactive where most calls were made during office hours, while out‐of‐office hours calls were initiated by family members; the politeness, the time given, and cordiality of the caller gave them a feeling that someone cared about their concerns. | 3 |
| Liew, M.F. | Professionals | Addressing psychological well-being of staff | Elements- 1. To provide emotional support, encouragement and appreciation to healthcare workers 2. Reduce stigmatization of healthcare workers by ill-informed members of the public  Solutions 1. Special provision of meals and drinks to boost morale; laundry service for used scrubs 2. Provision of regular updates of the local situation and status by the government and institution leadership 3. Frequent encouragement of HCW by divisional heads and senior leaders via emails, messaging apps and social media platforms, allowing staff to remain engaged 4. Timely articles and courageous stories of frontline staff 5. Appropriate media coverage of HCW at the frontline to increase empathy and reduce stigmatization | Unspecified | Not reported | Not reported | Yes | Yes | Intensive care units outside of China need to prepare for a potential surge of critically ill patients and counter the high transmissibility of COVID-19. | 3 |
| Webb, H. | Families | Videoconferencing | 1. Following collaboration with our Information Management and Technology team, four iPads were purchased and restricted to Facetime software 2. Relatives were offered the opportunity to have a ‘virtual visit’ through a video call 3. If accepted, a preparatory explanation of what would be seen was given to the relative in an attempt to minimise any distress 4. Afterwards staff on the unit in PPE would call with the iPad at the bedside | Unspecified | Not reported | Not reported | Yes | Yes | Videoconferencing technology has the potential to be extended to clinical use, for example when seeking advice for colleagues, and to the provision of training. | 3 |
| Kotfis, K. | Patients | COVID-19 delirium management considerations via the Society of Critical Care Medicine’s 'ABCDEF' safety bundle framework for F- family presence | 1. Orientate both patients and family regularly, provide phone conversations and video conferences, use technology devices, headphones, and tele-medicine tools 2. Provide visual and vocal contact with the family/ caregivers/friends, especially for all dying patients despite isolation, lack of time, and heavy workload | Empirical and theory | Not reported | Not reported | No | No | Implementation at the bedside of excellent delirium prevention and management should be a priority during the COVID-19 pandemic. | 2 |
| Lau, J. | Families, professionals | Telehealth solutions | Not reported | Empirical and theory | Not reported | Provider time; availability of technology | No | No | Telehealth enhanced patient–family communication, postdischarge follow-up, and palliative care for patients with COVID-19. | 2 |
| Zeneli, A. | Patients, families, professionals | Key policies and mitigating strategies and nursing response for cancer care management | 1. A coordinated emergency action plan permitting duty of care within the context of a pandemic, and care pathway revision 2. Rapid implementation of strategies and policies for a nursing response to the new care scenarios: personnel redistribution, nursing workflow revision, acquisition of new skills and knowledge, effective communication strategies, infection control policies, risk assessment and surveillance programmes, and continuous supplying of personal protective equipment 3. Within a pandemic context, clear nursing policies reinforcing the role of nurses as patient and caregiver educators are needed to promote infection prevention behaviour in the general population | Empirical and theory | Not reported | Not reported | No | No | In addition to contributing to the efficacy of the mitigating strategies, nurses also played an active role in ensuring the appropriate use of health resources and in increasing the benefit for patients and the community. | 2 |
| Estella, A. | Patients, families | Measures to improve communication between critically ill patients in isolation and families | 1. Through videoconferencing, healthcare professionals can first provide daily clinical information updates to patients’ family members, allowing families to see their loved ones through the screen of mobile phones 2. When patients recover a level of consciousness and are able to understand their condition and communicate with loved ones, this can be extended to allow communication between health-care professionals, patients, and their family 3. Provide patients with personal objects provided by family members such as photos, radios set to patients’ favorite stations, and even letters of encouragement from family members and anonymous volunteers who want to contribute to the care of patients, which are read to patients by the healthcare staff 4. Patients may be accompanied at the end of life by a single family member, after we determine that the family member has no symptoms of infection on arrival at the hospital, and after we explain the situation of terminal illness and the risks the family member assumes when he or she accompanies the patient at the end of life, and after we provide the family member with individual protective equipment that we help put on and later remove 5. After the patient’s death, there is clinical follow up with the family member, who must agree to comply with confinement measures at home, and to alert the healthcare team if symptoms appear in the next 14 days | Empirical only | Not reported | Not reported | No | No | Quality care in modern medicine must ensure and improve end-of-life care for the critically ill, whether or not the cause of hospital admission was COVID-19 infection. | 2 |
| Ott, M.A. | Patients | Urgent questions to address for sexual and reproductive health | 1. How are current policies affecting sexual and reproductive health rights and outcomes? What gaps or inequities are being created or exacerbated? 2. Are these policies just? Do they disproportionately hurt specific populations? Whom are we excluding? What services should be considered essential? 3. What action can be taken now to minimize restrictions on sexual and reproductive rights and on any resulting inequities? Are there alternative policies that preserve both important public health benefits and basic sexual and reproductive health rights and services? | Empirical only | Not reported | Not reported | No | No | It is important to have in place the structures, strategies and stakeholder partnerships needed to mitigate damage to basic human rights to sexual and reproductive health, and also the ethical framework necessary to make just and equitable decisions about balancing individual human rights and public health. | 2 |
| Owens, I.T. | Professionals | Interventions to support resilience and mental health for nurses | 1. When PPE is not in short supply, allow family support at the bed- side 2. Establish programs to provide smartphones and tablets to patients to enable them to communicate with families remotely 3. Mobilize all staff with palliative care experience to the hospital’s COVID-19 units and provide brief education for frontline staff on symptom management 4. Designate nonnursing staff whose primary role is facilitating communication between family and patients 5. Educate staff about palliative and end-of-life nursing care 6. Establish organizational policies and procedures that recognize and prevent risk factors for secondary traumatic stress, the emotional distress that may develop when individuals learn about the traumatic experience of others 7. Provide education on mental hygiene, making mental health services accessible to all staff and promoting mental health services as compassionate care for the caregiver 8. Recognize that many healthcare workers are the sole support of others in the family who may have lost employment 9. Implement mechanisms to assess the mental wellness of staff in real time 10. Increase communication 11. Establish a hotline staff can use for any type of need or concern; for example, lack of equipment, emotional support, short staffing, or financial counseling 12. Operationalize existing critical incident stress debriefing teams provided for professionals exposed to highly stressful and traumatic events | Empirical only | Not reported | Not reported | No | No | A nurse’s ability to survive depends on healthcare organizations understanding that this is not business as usual, and that the crisis is ongoing. | 2 |
| Rubin, G.A. | Families | Strategies to manage inpatient electrophysiology consultations | 1. Telephone consultation with COVID+ inpatients 2. Discussions over the phone with family/friends (who are not able to remain with patients during this time period) 3. Daily meetings also present a much-needed opportunity to decompress and maintain positive morale among staff | Empirical only | Not reported | Not reported | No | No | Regularly scheduled weekly electrogram conferences and case reviews helps to maintain a sense of camaraderie and 'normalcy' among professionals. | 2 |
| Choi, K.R. | Patients, families | Trauma-informed approach to perinatal care for women and infants | 1. Promoting women’s control and choice whenever possible 2. Acknowledging the effects of COVID-19 on their births and early parenting experiences 3. Using a collaborative approach to ensure that their mental, physical, emotional, and social needs are met 4. A trauma-informed care approach involves recognizing and responding to these and other symptoms of trauma and actively seeking to avoid triggers and retraumatization while providing care | Theory only | Training or education in psychology | Not reported | No | No | Nurses often have sustained contact and relationships with women and their infants during the continuum of maternity care, and are uniquely positioned to provide maternal and infant health interventions as members of the perinatal care team. | 2 |
| Cook, D.J. | Patients, families | Compassionate and consistent communication with families | 1. Clinicians implemented humanistic interventions to try to ameliorate patient isolation 2. Hand signals, cellphones, baby monitors, 2-way radios, and tablets were used to communicate between and among staff, patients, and families through glass doors 3. Biding at the bedside for dying patients whenever possible 4. Clinicians were compelled to bridge the distance between patients and relatives by telephoning families, particularly if patients could not call or text themselves | Theory only | Not reported | Familial reluctancy | No | No | Commemorating patients by learning about them as individuals, while inviting conversation and welcoming familiar, feasible rituals, may bring comfort to patients and their families. | 2 |
| DeJong, A.J. | Patients | Provider wellness techniques; general strategies of cognitive behavioural therapy | 1. Challenging unhelpful thoughts 2. Encouraging ake a balanced perspective, easing physical stress and tension 3. Engaging in helpful behavior | Theory only | Training or education in psychology | Not reported | No | No | The triad of thoughts, feelings, and behavior forms a firm basis for conducting therapeutic conversations from a behavioral therapeutic framework; it starts by making a functional analysis and challenging irrational thoughts and looking together for more rational thoughts that can reduce negative feelings. | 2 |
| Ferrell, B.R. | Patients, families, professionals | Spiritual care within palliative care | 1. Every palliative care clinician can become more attuned to their role as a spiritual care generalist, by intentionally focusing on the quality of their therapeutic presence and communication and by learning specific skills designed to uncover spiritual concerns of patients during times of both normalcy and crisis 2. Spiritual injury, spiritual distress, and suffering are likely to be exacerbated at the intersection of COVID-19, widespread health system strain, and the burden of serious illness and caregiver overload 3. All clinicians are responsible for identifying the need for spiritual specialty care and advocating for consultation and involvement as soon as possible 4. Given the complex psychosocial and spiritual impacts associated with COVID-19, as well as limited numbers of chaplains, it is vital that patients receive spiritual assessment and be invited to express themselves spiritually during encounters 5. Given the urgent need for spiritual assessment and care, we recommend that all health care professionals be educated through programs such as the Interprofessional Spiritual Care Education Curriculum 6. We must forge innovative and clear pathways for chaplains to provide reliable spiritual care services throughout the pandemic and beyond as chaplains make comfort rounds to families camped outside hospital doors, holding vigil for their loved ones | Theory only | Chaplain and spiritual care training | Not reported | No | No | Clinicians should be mindful to readdress spiritual needs throughout the care continuum for patients and families during escalation of illness, surrounding the time of death, and throughout the rehabilitation phase as applicable. | 2 |
| Garrett, J.R. | Families | Strategies to enhance family morale | 1. Design new visitor policies in these care contexts with morale in mind 2. Evaluate based on whether they could be effectively maintained indefinitely 3. Evaluate whether the potential for marginal improvements in infection control outweigh the definite reality of significant disruptions to family-centered care | Theory only | Training or education in bioethics | Not reported | No | No | Decision-making that aims to preserve and support morale must prioritize durable policies and procedures. | 2 |
| Hossain, F. | Families, professionals | Real Talk Real Time' virtual visits | 1. One group of creative nurses implemented virtual rounds for the families of patients who could not visit in person due to COVID-19 restrictions, and brought the families and patients together 2. Another grou focused on nurse self-care strategies 3. One way to self-care is by building moral resilience that is the courage and confidence to confront distressful and uncertain situations by following and trusting values and beliefs 4. Another way is through self-stewardship, which is the skill of tending to and nurturing one’s well-being 5. Cognitive processing therapy is a form of cognitive behavioral treatment to help victims of trauma, and there are four main steps which include education, information, developing skills, and changing beliefs 6. Through this, trauma therapists can help the nurses to identify possible symptoms of posttraumatic stress disorder and lead them to understand how receiving treatment can help | Theory only | Training or education in psychology | Not reported | No | No | Discounting or ignoring the mental health of nurses will have some adverse short- and-long-term consequences for the healthcare delivery system. | 2 |
| Janssen, D.J.A. | Families, professionals | Consensus recommendations for palliative care for patients with COVID-19 | 1. Advanced care planning should be routinely performed or reviewed by clinicians with patients and their loved ones at diagnosis of serious COVID-19 2. Adavanced care planning should be re-evaluated prior to discharge of recovered COVID-19 patients from hospital 3. Patients presenting with serious COVID-19 and distressing breathlessness despite optimal treatment of underlying causes should be given low-dose opioids for the palliative treatment of breathlessness 4. Patients presenting with serious COVID-19 and distressing breathlessness despite optimal treatment of underlying causes should be given benzodiazepines for the palliative treatment of breathlessness 5. Patients with serious COVID-19 in palliative care and distressing breathlessness should be given oxygen therapy for the palliative treatment of breathlessness when their transcutaneous oxygen saturation is <90% 6. Staff taking care of patients with serious COVID-19 should receive training in optimising clinician–patient communication whilst wearing PPE 7. Staff taking care of patients with serious COVID-19 should receive training in online clinician–family communication (while using telephone or video conferencing) 8. Healthcare professionals trained in providing palliative care should be involved in cases where hospitalised patients with serious COVID-19 have persistent symptoms and concerns despite optimal disease treatment 9. Healthcare professionals trained in providing palliative care should be involved in cases where patients with serious COVID-19 have persistent symptoms and concerns despite optimal disease treatment, and are being treated at home 10. Healthcare professionals providing spiritual care (such as chaplains) should be part of the treatment team of patients with serious COVID-19 with persistent symptoms and concerns despite optimal disease treatment (irrespective of setting, so in the hospital, community or long-term care facilities) 11. Healthcare professionals providing psychosocial care (such as psychologists and social workers) should be part of the treatment team of patients with serious COVID-19 with persistent symptoms and concerns despite optimal disease treatment (irrespective of setting, so in the hospital, community or long-term care facilities) 12. Family members/loved ones should be invited and supported (e.g. being provided with PPE if indicated) to visit the dying patient with COVID-19 in person 13. Family members/loved ones of deceased patients with COVID-19 should be offered bereavement support by healthcare professionals trained in palliative care or bereavement support | Theory only | Training or education in palliative care | Not reported | No | No | Future studies are needed to provide empirical evidence for these recommendations. | 2 |
| Agostoni, P. | Families | Daily phone calls to caregivers | Not reported | Unspecified | Not reported | Not reported | Yes | Yes | Reorganization of care in a dynamic, proactive manner, and to take rapid, arbitrary, innovative decisions was helpful. | 2 |
| Anderson-Shaw, L.K. | Patients, families | Cellphone use by non-critical patients | Not reported | Unspecified | Not reported | Not reported | Yes | Yes | The emotional toll for healthcare workers who have battled on the front lines is multifactorial and will last a lifetime; learning about their moral conflicts and distress will assist professionals now and in the future. | 2 |
| Damani, A. | Patients, families | Position statement from the Indian Association of Palliative Care | 1. Adapt the basic principles of pediatric palliative care such as family-centered care, effective pain, and symptom management, quality and dignity at end of life, honest communication, shared decision-making of treatment goals, and provision of grief and bereavement support to children and their families affected by the COVID-19 pandemic 2. The suffering of patients with cancer and other noncommunicable needs is heightened during this COVID-19 situation and should be recognized, and patients and families should be supported by the multidisciplinary palliative care teams (physical consultations when possible otherwise through telephone/video conference calls) 3. Telehealth can also be used as a source for identifying, assessing, and providing adequate psychological, social, and spiritual issues that contribute to increased distress. 4. Loss, grief, and bereavement can be complicated in patients with COVID-19 and should be assessed in all patients and their family members and interventions should be initiated early | Unspecified | Training or education in palliative care | Not reported | No | No | All professionals have a responsibility to provide good symptom management and end-of-life care in seriously ill patients with COVID-19 who are not eligible for or not responding to intensive care treatment. | 2 |
| Montauk, T.R. | Families | Steps to mitigate the trauma experienced by families | 1. Acknowledge the uniqueness and difficulty of the situation 2. Initiate video conferencing with mobile tools as early into the treatment regimen as possible—but only if the family is amenable 3. Do not be afraid to show emotion 4. Call it like it is | Unspecified | Training and experience in communication strategies | Not reported | No | No | It will be crucial for health care professionals to ensure families receive referrals for mental health services to relieve symptoms and prevent adverse outcomes associated with untreated psychiatric illness. | 2 |
| Dayal, A.K. | Patients, families | A 24/7 patient-family call center | Not reported | Empirical only | Not reported | Not reported | No | No | Flexibility to adapt resources and workforce to the situation, regular, frequent and calming communications, and hospital leaders committed and devote to the situation helped professionals to care for all patients. | 1 |
| Mehta, A.K. | Patients, families | Video visits for palliative care | Not reported | Empirical only | Not reported | Not reported | No | No | Video visits provide a form of face-to-face communication and allow multiple health care professionals to engage patients/ families/caregivers simultaneously to continue providing early palliative care services. | 1 |
| Glancy, D. | Patients | One-to-one and group support sessions | Not reported | Theory only | Training or education in psychology | Not reported | No | No | Many patients found the cessation of visits from family members with whom they had an ambivalent relationship helpful. | 1 |
| Rao, S.R. | Patients | Components of psychosocial care within palliative care | 1. Communication; achieved by pasting photos/names on to PPE for self‐introduction, by communicating verbally using words expressing empathy, and to have frequent, clear, and honest discussions regarding care goals 2. Delivery; essential to maintain trust and hope when the practices of therapeutic touch, attending to nonverbal communication, and physical presence are impeded | Theory only | Not reported | Not reported | No | No | Public health emergencies are known to affect psychological well‐being of individuals, and the effects can be short term, or can progress to psychopathology, with some groups being more vulnerable than others. | 1 |
| Tanzi, S. | Professionals | Addressing challenges for palliative care specialists | 1. Supporting the staff in communicating bad news by phone 2. Training the entire staff on symptom management and control (in particular agitation and dyspnea) 3. Supporting the entire staff in dealing with suffering and patients’ deaths 4. Guaranteeing a peaceful death necessarily inside the hospital 5. Changing the way of breaking bad news, with patient alone in a one-to-one conversation 6. Being the link with the psychologists outside the hospital 7. Being aware of our compassionate presence during the short visits, which restores dignity and humanity 8. Personalized communication to meet patients’ specific needs 9. Being the bridge between hospitalized patients and their families | Theory only | Not reported | Not reported | No | No | A hospital-based palliative care unit seems to be able to contribute significantly to the management of end-of-life care. | 1 |
| Anneser, J. | Patients, families, professionals | Tools for electronic communication | 1. Simple measures like the provision of free telephone cards (some patients do not bring a suitable charging cable for their mobile) may be helpful 2. Psychosocial and spiritual care of patients, relatives, and healthcare professionals are of paramount importance | Unspecified | Not reported | Not reported | No | No | Hospital palliative care professionals should share experiences from the past weeks and prepare for the challenges we will face during the next expected pandemic outbreaks. | 1 |
| Feng, D. | Professionals | Psychological adjustment for medical staff | Elements- 1. Maintain a normal pace of life 2. Adjust a good psychological status 3. Friendly and supportive social favor 4. Seek professional psychological help  Steps- 1. Reasonable analysis of the reasons for emotions; limited staff and complicated work; dual pressures of physical strength and energy; pressure from patients and family members; lack of medical protection supplies; complete worry about family members and themselves 2. Recognize and accept your emotions correctly; during the epidemic, medical staff are in a state of high workload for a long time, physically and mentally exhausted, and the doctor-patient relationship across the country is very tense, which will inevitably produce emotions such as irritability, worry, fear, anger and even helplessness; we should accept the existence of emotions, and take appropriate adjustment measures according to our own conditions, such as communicating more with others, encouraging each other, and even seeking help from a psychologist 4. Actively adjust your physical and mental state: help you adjust your emotions in various ways, including diverting your attention, abdominal breathing, progressive muscle relaxation exercises, meditation, hot baths, etc. | Unspecified | Not reported | Not reported | No | No | Strengthening the first diagnosis responsibility system, early detection, early diagnosis and timely isolation of suspected COVID-19 patients is important for patient and family management. | 1 |
| Overton, J. | Patients | Process for enhanced vigilance for compassionate care | 1. Nursing leaders were available at each entrance to assist, in real-time, with any difficult conversations 2. Reassigned employees were available to escort a number of patients to their appointments 3. Any messages were delivered with compassion and professionalism to our patients and their families 4. Explaining that we were placing their safety first and foremost | Unspecified | Not reported | Not reported | No | No | The onsite Patient Advocate, along with frontline staff, provided the important rationale for visitor restrictions; this shared decision making empowered those providing care on the front line. | 1 |
| Rosa, W. E. | Patients, families | Communication and whole-person care | 1. The use of technology should be encouraged to address patient isolation, improve communication with family and the interdisciplinary team, and improve assessment of personal or disease-related needs 2. Spiritual, religious, and other end-of-life care needs should be elicited upon consultation for the COVID-19–positive patient to ensure proactive person-centered care, even when the physical presence of the palliative nurse at the time of death may not be feasible 3. Additionally, facilities heavily impacted by COVID-19, such as long-term care, skilled nursing, and assisted living, would benefit from palliative nursing input to mitigate patient distress and assist in supporting staff members with coping strategies | Unspecified | Not reported | Not reported | No | No | Investment in palliative nurses and nursing during COVID-19 will strengthen the broader health system's resilience and capacity to respond effectively to future pandemics and public health crises. | 1 |
| Tey, J. | Professionals | Ensuring continuity of communication channels | 1. Electronic messaging systems 2. Emails and meetings using video or web conferencing 3. Plan for and maintain both hardware and software requirements 4. Fast internet connection | Unspecified | Not reported | Not reported | No | No | Sustained delivery of essential radiotherapy services can only be mitigated by national and institutional policy. | 1 |
| Costantini, M. | Families | Hospice psychologist telephoning patients’ relatives every day | Not reported | Theory only | Not reported | Not reported | No | No | The hospice sector is able to respond flexibly and rapidly to the COVID-19 pandemic; however, the potential of hospices in supporting the COVID-19 pandemic will be undermined unless the sector has access to appropriate protective equipment and setting-specific guidance. | 0 |
| Fadul, N. | Patients | Bereavement interventions and counselling | Not reported | Theory only | Not reported | Not reported | No | No | There are immediate needs for healthcare provider education on palliative care principles and how to triage patients when resources are scare, alternative methods for provision of palliative care such as telemedicine, telecounselling and online bereavement support groups, and to document outcomes in order to assist the healthcare system with preparedness for future pandemics. | 0 |
| Morley, G. | Professionals | Relational account of care to current nursing practice realities | Not reported | Theory only | Not reported | Not reported | No | No | Nurses’ voices and perspectives must be integrated into both local and global decision-making so as to minimize potential structural injustices. | 0 |
| Al-Shamsi, H.O. | Patients, families | Preparing psychosocial staff to be more utilized to assess and address distress | Not reported | Unspecified | Not reported | Not reported | No | No | It is important that supports are in place in each cancer program and hospital to assess the level of distress and intervene appropriately to the best of the available resources. | 0 |
| Alhalabi, O. | Patients, families | Frequent telemedicine visits with short-term follow-up | Not reported | Unspecified | Not reported | Not reported | No | No | The dynamic healthcare response to COVID-19 highlights that it is vital to document advanced care planning for each patient, sometimes aided by the use of telehealth. | 0 |
| Fahed, M. | Patients | Management of behavioral and psychological symptoms in dementia | Not reported | Unspecified | Not reported | Not reported | No | No | Caring for older adults in times of COVID-19 brings a specific set of challenges that highlight the complex interplay between facilities, units, staff members, and patients who are especially vulnerable to the virus. | 0 |
| Ford, S. | Professionals | Charity-run helpline | Not reported | Unspecified | Not reported | Not reported | No | No | Telephone support via a dedicated helpline was not a preferred method of support; more popular was informal peer support from colleagues, and individual face-to-face support with a mental health professional. | 0 |
| Leira, E.C. | Patients, families | Two-way audiovisual or telephone communication | Not reported | Unspecified | Not reported | Not reported | No | No | It is important to provide the best care possible to patients with stroke and their families and advocate for their best interests, at a time when social distancing limits social support in most settings. | 0 |
| Valdes, E. | Families | Family visitation prior to cardiopulmonary arrest/after brain death assessment and prior to discontinuation of organ support | Not reported | Unspecified | Not reported | Not reported | No | No | It is feasible to evaluate patients with catastrophic brain injury and declare brain death despite the COVID-19 pandemic, but this requires unique considerations. | 0 |

Organized in order of score; derivation; operationalized; generalizable

^1^Included in the steps described in the record

^2^Brief description of the approach

^3^Steps or guiding principles to conduct the approach (N.B. full steps are provided even if family visitation/presence is one or few components)

^4^Approach is derived from empirical evidence (i.e., through observation or experiment) or from published theory

^5^Minimum expertise required to conduct the approach

^6^Limitations to the approach

^7^Is the approach reproducible? (i.e., evidenced by use at multiple acute care settings)

^8^Can the approach can be feasibly applied to other contexts? (N.B. decisions were made based on that internal validity should precede external validity)

^9^One point was given for each of: steps described; based on empirical evidence; expertise considered; limitations reported; operationalized; and generalizable

^*^As reported in the record

^#^As determined by the review team

Online Appendix 1. Alphabetical references for included reports [1-155]

1. **The Right Not to be Forced to Die Alone — The National Catholic Bioethics Center**. In*.*; 2020.

2. **Re-integration of family caregivers as essential partners covid-19**. In*.*: Canadian Foundation for Healthcare Improvement; 2020.

3. **Virtual Visits Connect Hospitalized Patients With Family Members – Consult QD**. In*.*; 2020.

4. **590 people’s stories of leaving hospital during COVID-19**. In*.*: British Red Cross; 2020.

5. Abbott J, Johnson D, Wynia M: **Ensuring Adequate Palliative and Hospice Care During COVID-19 Surges**. *JAMA* 2020, **324**(14):1393-1394.

6. Abrams EM, Shaker M, Oppenheimer J, Davis RS, Bukstein DA, Greenhawt M: **The Challenges and Opportunities for Shared Decision Making Highlighted by COVID-19**. *The Journal of Allergy & Clinical Immunology in Practice* 2020, **8**(8):2474-2480.e2471.

7. Agostoni P, Mapelli M, Conte E, Baggiano A, Assanelli E, Apostolo A, Alimento M, Berna G, Guglielmo M, Muratori M *et al*: **Cardiac patient care during a pandemic: how to reorganise a heart failure unit at the time of COVID-19**. *European Journal of Preventive Cardiology* 2020, **27**(11):1127-1132.

8. Al-Jabir A, Kerwan A, Nicola M, Alsafi Z, Khan M, Sohrabi C, O'Neill N, Iosifidis C, Griffin M, Mathew G *et al*: **Impact of the Coronavirus (COVID-19) pandemic on surgical practice - Part 1**. *International Journal Of Surgery* 2020, **79**:168-179.

9. Al-Shamsi HO, Alhazzani W, Alhuraiji A, Coomes EA, Chemaly RF, Almuhanna M, Wolff RA, Ibrahim NK, Chua MLK, Hotte SJ *et al*: **A Practical Approach to the Management of Cancer Patients During the Novel Coronavirus Disease 2019 (COVID-19) Pandemic: An International Collaborative Group**. *Oncologist* 2020, **25**(6):e936-e945.

10. Alhalabi O, Subbiah V: **Managing Cancer Care during the COVID-19 Pandemic and Beyond**. *Trends in Cancer* 2020, **6**(7):533-535.

11. Alsharaydeh I, Rawashdeh H, Saadeh N, Obeidat B, Obeidat N: **Challenges and solutions for maternity and gynecology services during the COVID-19 crisis in Jordan**. *International Journal of Gynecology & Obstetrics* 2020, **150**(2):159-162.

12. Anderson-Shaw LK, Zar FA: **COVID-19, Moral Conflict, Distress, and Dying Alone**. *Journal of bioethical inquiry* 2020, **17**:777-782.

13. Anneser J: **Dying patients with COVID-19: What should Hospital Palliative Care Teams (HPCTs) be prepared for?** *Palliative & Supportive Care* 2020, **18**(4):382-384.

14. Apoeso O, Kuwata C, Goldhirsch SL, Piracha N, Reyes-Arnaldy A, De Leon J, Chai E: **Creating a Colocation Unit for End-of-Life Care during a Pandemic**. *Journal of palliative medicine* 2020, **Online ahead of print**.

15. Arjomandi Rad A, Vardanyan R: **Surgery in the COVID-19 era: implications for patient's mental health and practical recommendations for surgeons**. *British Journal of Surgery* 2020, **107**(10):e388.

16. Arnetz JE, Goetz CM, Arnetz BB, Arble E: **Nurse Reports of Stressful Situations during the COVID-19 Pandemic: Qualitative Analysis of Survey Responses**. *International journal of environmental research and public health* 2020, **17**(21):8126.

17. Arora KS, Mauch JT, Gibson KS: **Labor and Delivery Visitor Policies During the COVID-19 Pandemic: Balancing Risks and Benefits**. *JAMA: Journal of the American Medical Association* 2020, **323**(24):2468-2469.

18. Arya A, Buchman S, Gagnon B, Downar J: **Pandemic palliative care: beyond ventilators and saving lives**. *CMAJ: Canadian Medical Association Journal* 2020, **192**(15):E400-E404.

19. Atreya S, Kumar G, Samal J, Bhattacharya M, Banerjee S, Mallick P, Chakraborty D, Gupta S, Sarkar S: **Patients'/Caregivers' perspectives on telemedicine service for advanced cancer patients during the COVID-19 pandemic: An exploratory survey**. *Indian Journal of Palliative Care* 2020, **26**:40-44.

20. Azoulay E, Cariou A, Bruneel F, Demoule A, Kouatchet A, Reuter D, Souppart V, Combes A, Klouche K, Argaud L *et al*: **Symptoms of Anxiety, Depression, and Peritraumatic Dissociation in Critical Care Clinicians Managing Patients with COVID-19. A Cross-Sectional Study**. *American Journal of Respiratory & Critical Care Medicine* 2020, **202**(10):1388-E1313.

21. Azoulay E, De Waele J, Ferrer R, Staudinger T, Borkowska M, Povoa P, Iliopoulou K, Artigas A, Schaller SJ, Hari MS *et al*: **Symptoms of burnout in intensive care unit specialists facing the COVID-19 outbreak**. *Annals of intensive care* 2020, **10**(1):110.

22. Bambi S: **COVID-19 in Critical Care Units: Rethinking the Humanization of Nursing Care**. *Dimensions of Critical Care Nursing* 2020, **39**(5):239-241.

23. Belli LF: **[Recommendations for communicating bad news by phone during the SARS-CoV-2 pandemicRecomendacoes para a comunicacao de mas noticias por telefone durante a pandemia do SARS-CoV-2]**. *Recomendaciones para la comunicacion de malas noticias por telefono durante la pandemia por SARS-CoV-2* 2020, **44**:e69.

24. Benaque A, Gurruchaga MJ, Abdelnour C, Hernandez I, Canabate P, Alegret M, Rodriguez I, Rosende-Roca M, Tartari JP, Esteban E *et al*: **Dementia Care in Times of COVID-19: Experience at Fundacio ACE in Barcelona, Spain**. *Journal of Alzheimer's Disease* 2020, **76**(1):33-40.

25. Bostan S, Akbolat M, Kaya A, Ozata M, Gunes D: **Assessments of Anxiety Levels and Working Conditions of Health Employees Working in COVID-19 Pandemic Hospitals**. *Electronic Journal of General Medicine* 2020, **17**(5):1-5.

26. Bourgeault IL, Maier CB, Dieleman M, Ball J, MacKenzie A, Nancarrow S, Nigenda G, Sidat M: **The COVID-19 pandemic presents an opportunity to develop more sustainable health workforces**. *Human resources for health* 2020, **18**(1):83.

27. BrintzenhofeSzoc K, Krok-Schoen JI, Pisegna JL, MacKenzie AR, Canin B, Plotkin E, Boehmer LM, Shahrokni A: **Survey of cancer care providers' attitude toward care for older adults with cancer during the COVID-19 pandemic**. *Journal of geriatric oncology* 2020, **Online ahead of print**.

28. Brucato A: **Voices from the frontline: Notes from a COVID-19 Emergency Unit in Milan, Italy...Dr. Antonio Brucato**. *Journal of Communication in Healthcare* 2020, **13**:76-78.

29. Buntzel J, Klein M, Keinki C, Walter S, Buntzel J, Hubner J: **Oncology services in corona times: a flash interview among German cancer patients and their physicians**. *Journal of Cancer Research & Clinical Oncology* 2020, **146**(10):2713-2715.

30. Butler CR, Wong SPY, Wightman AG, O'Hare AM: **US Clinicians' Experiences and Perspectives on Resource Limitation and Patient Care During the COVID-19 Pandemic**. *JAMA Network Open* 2020, **3**(11):1-15.

31. Cabarkapa S, Nadjidai SE, Murgier J, Ng CH: **The psychological impact of COVID-19 and other viral epidemics on frontline healthcare workers and ways to address it: A rapid systematic review**. *Brain, Behavior, and Immunity - Health* 2020, **8**:100144.

32. Chen H, Sun L, Du Z, Zhao L, Wang L: **A cross-sectional study of mental health status and self- psychological adjustment in nurses who supported Wuhan for fighting against the COVID-19**. *Journal of clinical nursing* 2020, **29**:4161–4170.

33. Chen J, Xiong M, He Z, Shi W, Yue Y, He M: **The enclosed ward management strategies in psychiatric hospitals during COVID-19 outbreak**. *Globalization and Health* 2020, **16**(1):53.

34. Cheng JOS, Li Ping Wah-Pun Sin E: **The effects of nonconventional palliative and end-of-life care during COVID-19 pandemic on mental health-Junior doctors' perspective**. *Psychological Trauma:Theory, Pesearch, Practice and Policy* 2020, **12**(S1):S146-S147.

35. Cherepanov E: **Responding to the Psychological Needs of Health Workers During Pandemic: Ten Lessons from Humanitarian Work**. *Disaster medicine and public health preparedness* 2020, **Sep 2020**:1-19.

36. Chevance A, Gourion D, Hoertel N, Llorca PM, Thomas P, Bocher R, Moro MR, Laprevote V, Benyamina A, Fossati P *et al*: **Ensuring mental health care during the SARS-CoV-2 epidemic in France: A narrative review**. *Encephale* 2020, **46**(3 Supplement):S3-S13.

37. Chew C, Ko D: **Medical ethics in the era of COVID-19: Now and the future**. *Respirology* 2020, **25**(10):1033-1034.

38. Cho HJ, Feldman LS, Keller S, Hoffman A, Pahwa AK, Krouss M: **Choosing Wisely in the COVID-19 Era: Preventing Harm to Healthcare Workers**. *Journal of Hospital Medicine (Online)* 2020, **15**(6):360-362.

39. Choi KR, Records K, Low LK, Alhusen JL, Kenner C, Bloch JR, Premji SS, Hannan J, Anderson CM, Yeo S *et al*: **Promotion of Maternal-Infant Mental Health and Trauma-Informed Care During the COVID-19 Pandemic**. *JOGNN - Journal of Obstetric, Gynecologic, & Neonatal Nursing* 2020, **49**(5):409-415.

40. Chua IS, Jackson V, Kamdar M: **Webside Manner during the COVID-19 Pandemic: Maintaining Human Connection during Virtual Visits**. *Journal of Palliative Medicine* 2020, **23**(11):1507-1509.

41. Cohen SP, Baber ZB, Buvanendran A, McLean BC, Chen Y, Hooten WM, Laker SR, Wasan AD, Kennedy DJ, Sandbrink F *et al*: **Pain Management Best Practices from Multispecialty Organizations During the COVID-19 Pandemic and Public Health Crises**. *Pain Medicine* 2020, **21**(7):1331-1346.

42. Cook DJ, Takaoka A, Hoad N, Swinton M, Clarke FJ, Rudkowski JC, Heels-Ansdell D, Boyle A, Toledo F, Dennis BB *et al*: **Clinician Perspectives on Caring for Dying Patients During the Pandemic : A Mixed-Methods Study**. *Ann Intern Med* 2020, **Online ahead of print**.

43. Correa DJ, Labovitz DL, Milstein MJ, Monderer R, Haut SR, Milstein M: **Folding a neuroscience center into streamlined COVID-19 response teams: Lessons in origami**. *Neurology* 2020, **95**(13):583-592.

44. Costantini M, Sleeman KE, Peruselli C, Higginson IJ: **Response and role of palliative care during the COVID-19 pandemic: A national telephone survey of hospices in Italy**. *Palliative Medicine* 2020, **34**(7):889-895.

45. Creutzfeldt CJ, Schutz REC, Zahuranec DB, Lutz BJ, Curtis JR, Engelberg RA: **Family Presence for Patients with Severe Acute Brain Injury and the Influence of the COVID-19 Pandemic**. *Journal of palliative medicine* 2020, **Online ahead of print**.

46. Damani A, Ghoshal A, Rao K, Singhai P, Rayala S, Rao S, Ganpathy K, Krishnadasan N, Verginia L, Vallath N *et al*: **Palliative care in coronavirus disease 2019 pandemic: Position statement of the Indian association of palliative care**. *Indian Journal of Palliative Care* 2020, **26**:3-7.

47. Dayal AK, Razavi AS, Jaffer AK, Prasad N, Skupski DW: **COVID-19 in obstetrics 2020: the experience at a New York City medical center**. *Journal of Perinatal Medicine* 2020, **48**(9):892-899.

48. DeJong CAJ, DeJong Verhagen JG, Pols R, Verbrugge CAG, Baldacchino A: **Psychological Impact of the Acute COVID-19 Period on Patients With Substance Use Disorders: We are all in this Together**. *Basic and clinical neuroscience* 2020, **11**(2):207-216.

49. Devlin JW, O'Neal HR, Jr., Thomas C, Barnes Daly MA, Stollings JL, Janz DR, Ely EW, Lin JC: **Strategies to Optimize ICU Liberation (A to F) Bundle Performance in Critically Ill Adults With Coronavirus Disease 2019**. *Critical care explorations* 2020, **2**(6):e0139.

50. Dhala A, Sasangohar F, Kash B, Ahmadi N, Masud F: **Rapid Implementation and Innovative Applications of a Virtual Intensive Care Unit During the COVID-19 Pandemic: Case Study**. *Journal of medical Internet research* 2020, **22**(9):e20143.

51. Dorman-Ilan S, Hertz-Palmor N, Brand-Gothelf A, Hasson-Ohayon I, Matalon N, Gross R, Chen W, Abramovich A, Afek A, Ziv A *et al*: **Anxiety and Depression Symptoms in COVID-19 Isolated Patients and in Their Relatives**. *Frontiers in psychiatry* 2020, **11**:581598.

52. Dudzinski DM, Hoisington BY, Brown CE: **Ethics Lessons From Seattle's Early Experience With COVID-19**. *American Journal of Bioethics* 2020, **20**(7):67-74.

53. Estella A: **Compassionate Communication and End-of-Life Care for Critically Ill Patients with SARS-CoV-2 Infection**. *The Journal of clinical ethics* 2020, **31**(2):191-193.

54. Etkind SN, Bone AE, Lovell N, Cripps RL, Harding R, Higginson IJ, Sleeman KE: **The Role and Response of Palliative Care and Hospice Services in Epidemics and Pandemics: A Rapid Review to Inform Practice During the COVID-19 Pandemic**. *Journal of Pain and Symptom Management* 2020, **60**(1):e31-e40.

55. Evans G: **A Nurse's Story: Hospitalized with COVID-19: 'I started feeling ... I am going to come through this'...Kay Ball**. *Hospital Infection Control & Prevention* 2020, **47**(7):1-3.

56. Fadul N, Elsayem AF, Bruera E: **Integration of palliative care into COVID-19 pandemic planning**. *BMJ supportive & palliative care* 2020, **0**:1-5.

57. Fahed M, Barron GC, Steffens DC: **Ethical and Logistical Considerations of Caring for Older Adults on Inpatient Psychiatry During the COVID-19 Pandemic**. *American Journal of Geriatric Psychiatry* 2020, **28**(8):829-834.

58. Fan PEM, Aloweni F, Lim SH, Ang SY, Perera K, Quek AH, Quek HKS, Ayre TC: **Needs and concerns of patients in isolation care units - learnings from COVID-19: A reflection**. *World journal of clinical cases* 2020, **8**(10):1763-1766.

59. Fang J, Liu YT, Lee EY, Yadav K: **Telehealth Solutions for In-hospital Communication with Patients Under Isolation During COVID-19**. *The Western Journal of Emergency Medicine* 2020, **21**(4):801-806.

60. Fausto J, Hirano L, Lam D, Mehta A, Mills B, Owens D, Perry E, Curtis JR: **Creating a Palliative Care Inpatient Response Plan for COVID-19-The UW Medicine Experience**. *Journal of Pain & Symptom Management* 2020, **60**(1):e21-e26.

61. Feinstein RE, Kotara S, Jones B, Shanor D, Nemeroff CB: **A health care workers mental health crisis line in the age of COVID-19**. *Depression & Anxiety* 2020, **37**(8):822-826.

62. Feng D, Liu S, Bai Y, Tang Y, Han P, Wei W: **Management of urology during COVID-19 pandemic: A perspective from Sichuan Province, China**. *International Journal of Surgery* 2020, **81**:115-121.

63. Ferrell BR, Handzo G, Picchi T, Puchalski C, Rosa WE: **The Urgency of Spiritual Care: COVID-19 and the Critical Need for Whole-Person Palliation**. *Journal of Pain & Symptom Management* 2020, **60**(3):e7-e11.

64. Ford S: **Covid-19: Negative impact on nurse mental health**. *Nursing Times* 2020, **116**(5):6-7.

65. Frampton S, Agrawal S, Guastello S: **Guidelines for Family Presence Policies During the COVID-19 Pandemic**. *JAMA Health Forum* 2020, **1**(7):e200807-e200807.

66. Fusi-Schmidhauser T, Preston NJ, Keller N, Gamondi C: **Conservative Management of COVID-19 Patients-Emergency Palliative Care in Action**. *Journal of Pain & Symptom Management* 2020, **60**(1):e27-e30.

67. Garrett JR, McNolty LA: **More than Warm Fuzzy Feelings: The Imperative of Institutional Morale in Hospital Pandemic Responses**. *American Journal of Bioethics* 2020, **20**(7):92-94.

68. Glancy D, Reilly L, Cobbe C, Glynn M, Punchoo S, Foy K: **Lockdown in a specialised rehabilitation unit: the best of times**. *Irish journal of psychological medicine* 2020, **37**(3):169-171.

69. Griffin KM, Karas MG, Ivascu NS, Lief L: **Hospital Preparedness for COVID-19: A Practical Guide from a Critical Care Perspective**. *American Journal of Respiratory & Critical Care Medicine* 2020, **201**(11):1337-1344.

70. Gupta A, Bahl B, Rabadi S, Mebane A, 3rd, Levey R, Vasudevan V: **Value of Advance Care Directives for Patients With Serious Illness in the Era of COVID Pandemic: A Review of Challenges and Solutions**. *The American journal of hospice & palliative care* 2020, **2020**:1-8.

71. Hall DE: **We Can Do Better: Why Pastoral Care Visitation to Hospitals is Essential, Especially in Times of Crisis**. *Journal of Religion & Health* 2020, **59**(5):2283-2287.

72. Hart JL, Turnbull AE, Oppenheim IM, Courtright KR: **Family-Centered Care During the COVID-19 Era**. *Journal of Pain & Symptom Management* 2020, **60**(2):e93-e97.

73. Hennein R, Lowe S: **A hybrid inductive-abductive analysis of health workers' experiences and wellbeing during the COVID-19 pandemic in the United States**. *PloS one* 2020, **15**(10):e0240646.

74. Holland DE, Vanderboom CE, Dose AM, Moore D, Robinson KV, Wild E, Stiles C, Ingram C, Mandrekar J, Borah B *et al*: **Death and Grieving for Family Caregivers of Loved Ones With Life-Limiting Illnesses in the Era of COVID-19: Considerations for Case Managers**. *Professional case management* 2020, **0**(0):1-9.

75. Horsch A, Lalor J, Downe S: **Moral and mental health challenges faced by maternity staff during the COVID-19 pandemic**. *Psychological Trauma:Theory, Pesearch, Practice and Policy* 2020, **12**(S1):S141-S142.

76. Hossain F, Clatty A: **Self-care strategies in response to nurses' moral injury during COVID-19 pandemic**. *Nursing ethics* 2020, **2020**:1-10.

77. Hron JD, Parsons CR, Williams LA, Harper MB, Bourgeois FC: **Rapid Implementation of an Inpatient Telehealth Program during the COVID-19 Pandemic**. *Applied Clinical Informatics* 2020, **11**(3):452-459.

78. Jain T, Jain R: **To Study the barriers in palliative care to nonmalignant cases in COVID-19 crisis in a tertiary health-care center**. *Indian Journal of Palliative Care* 2020, **26**:76-80.

79. Janssen DJA, Ekstrom M, Currow DC, Johnson MJ, Maddocks M, Simonds AK, Tonia T, Marsaa K: **COVID-19: guidance on palliative care from a European Respiratory Society international task force**. *European Respiratory Journal* 2020, **56**(3):2002583.

80. Jia Y, Chen O, Xiao Z, Xiao J, Bian J, Jia H: **Nurses' ethical challenges caring for people with COVID-19: A qualitative study**. *Nursing ethics* 2020, **2020**:1-13.

81. Kandori K, Okada Y, Ishii W, Narumiya H, Maebayashi Y, Iizuka R: **Association between visitation restriction during COVID-19 pandemic and delirium incidence among emergency admission patients: a single-center retrospective observational cohort study in Japan**. In*.* medRxiv : the preprint server for health sciences; 2020.

82. Karavadra B, Stockl A, Prosser-Snelling E, Simpson P, Morris E: **Women's perceptions of COVID-19 and their healthcare experiences: a qualitative thematic analysis of a national survey of pregnant women in the United Kingdom**. *BMC Pregnancy & Childbirth* 2020, **20**(1):600.

83. Karimi Z, Fereidouni Z, Behnammoghadam M, Alimohammadi N, Mousavizadeh A, Salehi T, Mirzaee MS, Mirzaee S: **The Lived Experience of Nurses Caring for Patients with COVID-19 in Iran: A Phenomenological Study**. *Risk management and healthcare policy* 2020, **13**:1271-1278.

84. Knights D, Knights F, Lawrie I: **Upside down solutions: palliative care and COVID-19**. *BMJ supportive & palliative care* 2020, **0**:1-5.

85. Kotfis K, Williams Roberson S, Wilson JE, Dabrowski W, Pun BT, Ely EW: **COVID-19: ICU delirium management during SARS-CoV-2 pandemic**. *Critical care (London, England)* 2020, **24**(1):176.

86. Kuntz JG, Kavalieratos D, Esper GJ, Ogbu N, Jr., Mitchell J, Ellis CM, Quest T: **Feasibility and Acceptability of Inpatient Palliative Care E-Family Meetings During COVID-19 Pandemic**. *Journal of Pain & Symptom Management* 2020, **60**(3):e28-e32.

87. Lai L, Sato R, He S, Ouchi K, Leiter R, deLima Thomas J, Lawton A, Landman AB, Mark Zhang H: **Usage Patterns of a Web-Based Palliative Care Content Platform (PalliCOVID) During the COVID-19 Pandemic**. *Journal of pain and symptom management* 2020, **60**(4):e20-e27.

88. Lam PT: **Self-reflections after disbandment of palliative care unit during COVID-19 pandemic**. *Hong Kong medical journal = Xianggang yi xue za zhi* 2020, **26**(5):463.

89. Landa-Ramírez E, Domínguez-Vieyra NA, Hernández-Nuñez ME, Díaz-Vásquez LP, Santana-García IA: **Psychological Support During COVID-19 Death Notifications: Clinical Experiences From a Hospital in Mexico**. *Psychological Trauma: Theory, Research, Practice & Policy* 2020, **12**:518-520.

90. Lau J, Knudsen J, Jackson H, Wallach AB, Bouton M, Natsui S, Philippou C, Karim E, Silvestri DM, Avalone L *et al*: **Staying Connected In The COVID-19 Pandemic: Telehealth At The Largest Safety-Net System In The United States**. *Health Affairs* 2020, **39**(8):1437-1442.

91. LeBlanc LA, Lazo-Pearson JF, Pollard JS, Unumb LS: **The Role of Compassion and Ethics in Decision Making Regarding Access to Applied Behavior Analysis Services During the COVID-19 Crisis: A Response to Cox, Plavnick, and Brodhead**. *Behavior analysis in practice* 2020, **13**:604-608.

92. Lee DR, Chavez K: **More Than Words: Reflections to Build Resilience during the COVID-19 Pandemic**. *The Permanente journal* 2020, **24**:20.

93. Leira EC, Russman AN, Biller J, Brown DL, Bushnell CD, Caso V, Chamorro A, Creutzfeldt CJ, Cruz-Flores S, Elkind MSV *et al*: **Preserving stroke care during the COVID-19 pandemic: Potential issues and solutions**. *Neurology* 2020, **95**(3):124-133.

94. Liew MF, Siow WT, MacLaren G, See KC: **Preparing for CovID-19: Early experience from an intensive care unit in Singapore**. *Critical Care* 2020, **24**(1):83.

95. Lissoni B, Del Negro S, Brioschi P, Casella G, Fontana I, Bruni C, Lamiani G: **Promoting Resilience in the Acute Phase of the COVID-19 Pandemic: Psychological Interventions for Intensive Care Unit (ICU) Clinicians and Family Members**. *Psychological Trauma: Theory, Research, Practice & Policy* 2020, **12**:S105-S107.

96. London V, McLaren R, Stein J, Atallah F, Fisher N, Haberman S, McCalla S, Minkoff H: **Caring for Pregnant Patients with COVID-19: Practical Tips Getting from Policy to Practice**. *American Journal of Perinatology* 2020, **37**(8):850-853.

97. Maves RC, Downar J, Dichter JR, Hick JL, Devereaux A, Geiling JA, Kissoon N, Hupert N, Niven AS, King MA *et al*: **Triage of Scarce Critical Care Resources in COVID-19 An Implementation Guide for Regional Allocation: An Expert Panel Report of the Task Force for Mass Critical Care and the American College of Chest Physicians**. *Chest* 2020, **158**(1):212-225.

98. McKee M, Moran C, Zazove P: **Overcoming Additional Barriers to Care for Deaf and Hard of Hearing Patients During COVID-19**. *JAMA Otolaryngology–Head & Neck Surgery* 2020, **146**(9):781-782.

99. McMahon M, Nadigel J, Thompson E, Glazier RH: **Informing Canada's Health System Response to COVID-19: Priorities for Health Services and Policy Research**. *Healthcare Policy = Politiques de sante* 2020, **16**(1):112-124.

100. Mehta AK, Smith TJ: **Palliative Care for Patients With Cancer in the COVID-19 Era**. *JAMA Oncology* 2020, **6**(10):1527-1528.

101. Mercadante S, Adile C, Ferrera P, Giuliana F, Terruso L, Piccione T: **Palliative Care in the Time of COVID-19**. *Journal of Pain & Symptom Management* 2020, **60**(2):e79-e80.

102. Mistraletti G, Gristina G, Mascarin S, Iacobone E, Giubbilo I, Bonfanti S, Fiocca F, Fullin G, Fuselli E, Bocci MG *et al*: **How to communicate with families living in complete isolation**. *BMJ supportive & palliative care* 2020, **Online ahead of print**.

103. Mitra M, Basu M: **A Study on Challenges to Health Care Delivery Faced by Cancer Patients in India During the COVID-19 Pandemic**. *Journal of primary care & community health* 2020, **11**:2150132720942705.

104. Mohindra R, R R, Suri V, Bhalla A, Singh SM: **Issues relevant to mental health promotion in frontline health care providers managing quarantined/isolated COVID19 patients**. *Asian Journal of Psychiatry* 2020, **51**:102084.

105. Molgora S, Accordini M: **Motherhood in the Time of Coronavirus: The Impact of the Pandemic Emergency on Expectant and Postpartum Women's Psychological Well-Being**. *Frontiers in psychology* 2020, **11**:567155.

106. Montauk TR, Kuhl EA: **COVID-related family separation and trauma in the intensive care unit**. *Psychological trauma : theory, research, practice and policy* 2020, **12**(S1):S96-S97.

107. Moore B: **Dying during Covid-19**. *Hastings Center Report* 2020, **50**(3):13-15.

108. Moore KJ, Sampson EL, Kupeli N, Davies N: **Supporting families in end-of-life care and bereavement in the COVID-19 era**. *International psychogeriatrics* 2020, **32**(10):1245-1248.

109. Morley G, Grady C, McCarthy J, Ulrich CM: **Covid-19: Ethical Challenges for Nurses**. *Hastings Center Report* 2020, **50**(3):35-39.

110. Morley G, Sese D, Rajendram P, Horsburgh CC: **Addressing caregiver moral distress during the COVID-19 pandemic**. *Cleveland Clinic Journal of Medicine* 2020, **87**:1-5.

111. Morris SE, Moment A, Thomas Jd: **Caring for Bereaved Family Members During the COVID-19 Pandemic: Before and After the Death of a Patient**. *Journal of Pain & Symptom Management* 2020, **60**(2):e70-e74.

112. Munch U, Muller H, Deffner T, von Schmude A, Kern M, Kiepke-Ziemes S, Radbruch L: **[Recommendations for the support of suffering, severely ill, dying or grieving persons in the corona pandemic from a palliative care perspective : Recommendations of the German Society for Palliative Medicine (DGP), the German Interdisciplinary Association for Intensive and Emergency Medicine (DIVI), the Federal Association for Grief Counseling (BVT), the Working Group for Psycho-oncology in the German Cancer Society, the German Association for Social Work in the Healthcare System (DVSG) and the German Association for Systemic Therapy, Counseling and Family Therapy (DGSF)]**. *Empfehlungen zur Unterstutzung von belasteten, schwerstkranken, sterbenden und trauernden Menschen in der Corona-Pandemie aus palliativmedizinischer Perspektive : Empfehlungen der Deutschen Gesellschaft fur Palliativmedizin (DGP), der Deutschen Interdisziplinaren Vereinigung fur Intensiv- und Notfallmedizin (DIVI), des Bundesverbands Trauerbegleitung (BVT), der Arbeitsgemeinschaft fur Psychoonkologie in der Deutschen Krebsgesellschaft, der Deutschen Vereinigung fur Soziale Arbeit im Gesundheitswesen (DVSG) und der Deutschen Gesellschaft fur Systemische Therapie, Beratung und Familientherapie (DGSF)* 2020, **34**(4):303-313.

113. Nelson R: **No-visitor policies cause anxiety and distress for patients with cancer**. In: *The Lancet Oncology.* Elsevier.

114. Ng KYY, Zhou S, Tan SH, Ishak NDB, Goh ZZS, Chua ZY, Chia JMX, Chew EL, Shwe T, Mok JKY *et al*: **Understanding the Psychological Impact of COVID-19 Pandemic on Patients With Cancer, Their Caregivers, and Health Care Workers in Singapore**. *JCO global oncology* 2020, **6**:1494-1509.

115. Ott MA, Bernard C, Wilkinson TA, Edmonds BT: **Clinician Perspectives on Ethics and COVID‐19: Minding the Gap in Sexual and Reproductive Health**. *Perspectives on Sexual & Reproductive Health* 2020, **52**(3):145-149.

116. Overton J, Denton K, Frumovitz M, Lewis C, Christensen S, Bezerra J, Hernandez C, Walker MS, Finder JP, Proske AA *et al*: **Coronavirus (COVID-19): Patient experience-Administrative services on the frontline during crisis**. *Head & Neck* 2020, **42**(7):1477-1481.

117. Owens IT: **Supporting nurses' mental health during the pandemic**. *Nursing* 2020, **50**(10):54-57.

118. Pahuja M, Wojcikewych D: **Systems Barriers to Assessment and Treatment of COVID-19 Positive Patients at the End of Life**. *Journal of palliative medicine* 2020, **Online ahead of print**.

119. Peña JA, Bianco AT, Simpson LL, Bernstein PS, Roman AS, Goffman D, Schweizer WE, Overbey J, Stone JL: **A Survey of Labor and Delivery Practices in New York City during the COVID-19 Pandemic**. *American Journal of Perinatology* 2020, **37**(10):975-981.

120. Pietrantonio F, Garassino MC: **Caring for Patients With Cancer During the COVID-19 Outbreak in Italy**. *JAMA Oncology* 2020, **6**(6):821-822.

121. Rao SR, Spruijt O, Sunder P, Daniel S, Chittazhathu RK, Nair S, Leng M, Sunil Kumar MM, Raghavan B, Manuel AJ *et al*: **Psychosocial Aspects of COVID-19 in the Context of Palliative Care - A Quick Review**. *Indian journal of palliative care* 2020, **26**(Suppl 1):S116-S120.

122. Ritchey KC, Foy A, McArdel E, Gruenewald DA: **Reinventing Palliative Care Delivery in the Era of COVID-19: How Telemedicine Can Support End of Life Care**. *The American journal of hospice & palliative care* 2020, **37**(11):992-997.

123. Rizvi Jafree S, ul Momina A, Naqi SA: **Significant other family members and their experiences of COVID-19 in Pakistan: A qualitative study with implications for social policy**. *Stigma and Health* 2020, **5**(4):380-389.

124. Romano M: **[Between intensive care and palliative care at the time of CoViD-19.]**. *Fra cure intensive e cure palliative ai tempi di CoViD-19* 2020, **111**(4):223-230.

125. Rosa WE, Davidson PM: **Coronavirus disease 2019 (COVID-19): strengthening our resolve to achieve universal palliative care**. *International Nursing Review* 2020, **67**(2):160-163.

126. Rosa WE, Gray TF, Chow K, Davidson PM, Dionne-Odom JN, Karanja V, Khanyola J, Kpoeh JDN, Lusaka J, Matula ST *et al*: **Recommendations to Leverage the Palliative Nursing Role During COVID-19 and Future Public Health Crises**. *Journal of hospice and palliative nursing : JHPN : the official journal of the Hospice and Palliative Nurses Association* 2020, **22**(4):260-269.

127. Rubin GA, Wan EY, Saluja D, Thomas G, Slotwiner DJ, Goldbarg S, Chaudhary S, Turitto G, Dizon J, Yarmohammadi H *et al*: **Restructuring Electrophysiology During the COVID-19 Pandemic: A Practical Guide From a New York City Hospital Network**. *Critical Pathways in Cardiology* 2020, **19**(3):105-111.

128. Rubin MA, Bonnie RJ, Epstein L, Hemphill C, Kirschen M, Lewis A, Suarez JI, Ethics L, Humanities Committee ajcotAAoNANA, Child Neurology S *et al*: **AAN position statement: The COVID-19 pandemic and the ethical duties of the neurologist**. *Neurology* 2020, **95**(4):167-172.

129. Samrah SM, Al-Mistarehi A-H, Aleshawi AJ, Khasawneh AG, Momany SM, Momany BS, Abu Za'nouneh FJ, Keelani T, Alshorman A, Khassawneh BY: **Depression and Coping Among COVID-19-Infected Individuals After 10 Days of Mandatory in-Hospital Quarantine, Irbid, Jordan**. *Psychology research and behavior management* 2020, **13**:823-830.

130. Sasangohar F, Dhala A, Zheng F, Ahmadi N, Kash B, Masud F: **Use of telecritical care for family visitation to ICU during the COVID-19 pandemic: an interview study and sentiment analysis**. *BMJ quality & safety* 2020, **0**:1-7.

131. Schoenherr LA, Cook A, Peck S, Humphreys J, Goto Y, Saks NT, Huddleston L, Elia G, Pantilat SZ: **Proactive Identification of Palliative Care Needs Among Patients With COVID-19 in the ICU**. *Journal of Pain & Symptom Management* 2020, **60**(3):e17-e21.

132. Selman LE, Chao D, Sowden R, Marshall S, Chamberlain C, Koffman J: **Bereavement Support on the Frontline of COVID-19: Recommendations for Hospital Clinicians**. *Journal of Pain & Symptom Management* 2020, **60**(2):e81-e86.

133. Semaan A, Audet C, Huysmans E, Afolabi B, Assarag B, Banke-Thomas A, Blencowe H, Caluwaerts S, Campbell OMR, Cavallaro FL *et al*: **Voices from the frontline: findings from a thematic analysis of a rapid online global survey of maternal and newborn health professionals facing the COVID-19 pandemic**. *BMJ Global Health* 2020, **5**(6):e002967.

134. Sinvani L: **The COVID‐19 Pandemic: Experiences of a Geriatrician‐Hospitalist Caring for Older Adults**. *Journal of the American Geriatrics Society* 2020, **68**:934-935.

135. Soh M, Hifumi T, Iwasaki T, Miura Y, Otani N, Ishimatsu S: **Impaired mental health status following ICU care in a patient with COVID-19**. *Acute medicine & surgery* 2020, **7**:e562.

136. Sprung CL, Joynt GM, Christian MD, Truog RD, Rello J, Nates JL: **Adult ICU Triage During the Coronavirus Disease 2019 Pandemic: Who Will Live and Who Will Die? Recommendations to Improve Survival**. *Critical care medicine* 2020, **48**(8):1196-1202.

137. Stephens AJ, Barton JR, Bentum N-AA, Blackwell SC, Sibai BM: **General Guidelines in the Management of an Obstetrical Patient on the Labor and Delivery Unit during the COVID-19 Pandemic**. *American Journal of Perinatology* 2020, **37**(8):829-836.

138. Stephens EH, Dearani JA, Guleserian KJ, Overman DM, Tweddell JS, Backer CL, Romano JC, Bacha E: **COVID-19: Crisis management in congenital heart surgery**. *Journal of Thoracic & Cardiovascular Surgery* 2020, **160**(2):522-528.

139. Stilos K, Moore JD: **REFLECTIONS ON PRACTICE How COVID-19 has changed the dying experience for acute care patients and their families**. *Canadian Oncology Nursing Journal / Revue canadienne de soins infirmiers en oncologie; Vol 30, No 3 (2020)* 2020, **30**(3):218-219.

140. Sun N, Wei L, Wang H, Wang X, Gao M, Hu X, Shi S: **Qualitative study of the psychological experience of COVID-19 patients during hospitalization**. *Journal of affective disorders* 2020, **278**:15-22.

141. Tan R, Yu T, Luo K, Teng F, Liu Y, Luo J, Hu D: **Experiences of clinical first‐line nurses treating patients with COVID‐19: A qualitative study**. *Journal of Nursing Management (John Wiley & Sons, Inc)* 2020, **28**(6):1381-1390.

142. Tanzi S, Alquati S, Martucci G, De Panfilis L: **Learning a palliative care approach during the COVID-19 pandemic: A case study in an Infectious Diseases Unit**. *Palliative Medicine* 2020, **34**(9):1220-1227.

143. Tey J, Ho S, Choo BA, Ho F, Yap SP, Tuan JKL, Leong CN, Cheo T, Sommat K, Wang MLC: **Navigating the challenges of the COVID-19 outbreak: Perspectives from the radiation oncology service in Singapore**. *Radiotherapy and Oncology* 2020, **148**:189-193.

144. Tingey JL, Bentley JA, Hosey MM: **COVID-19: Understanding and mitigating trauma in ICU survivors**. *Psychological trauma : theory, research, practice and policy* 2020, **12**(S1):S100-S104.

145. Turale S, Meechamnan C, Kunaviktikul W: **Challenging times: ethics, nursing and the COVID-19 pandemic**. *International Nursing Review* 2020, **67**(2):164-167.

146. Valdes E, Agarwal S, Carroll E, Kvernland A, Bondi S, Snyder T, Kwon P, Frontera J, Gurin L, Czeisler B *et al*: **Special considerations in the assessment of catastrophic brain injury and determination of brain death in patients with SARS-CoV-2**. *Journal of the Neurological Sciences* 2020, **417**:117087.

147. Viaux S, Maurice P, Cohen D, Jouannic JM: **Giving birth under lockdown during the COVID-19 epidemic**. *Journal of Gynecology Obstetrics and Human Reproduction* 2020, **49**(6):101785.

148. Wallace CL, Wladkowski SP, Gibson A, White P: **Grief During the COVID-19 Pandemic: Considerations for Palliative Care Providers**. *Journal of Pain & Symptom Management* 2020, **60**(1):e70-e76.

149. Webb H, Parson M, Hodgson LE, Daswani K: **Virtual visiting and other technological adaptations for critical care**. *Future healthcare journal* 2020, **7**(3):e93-e95.

150. Wei E, Segall J, Villanueva Y, Dang LB, Gasca VI, Gonzalez MP, Roman M, Mendez-Justiniano I, Cohen AG, Cho HJ: **Coping With Trauma, Celebrating Life: Reinventing Patient And Staff Support During The COVID-19 Pandemic**. *Health Affairs* 2020, **39**(9):1597-1600.

151. Wynne KJ, Petrova M, Coghlan R: **Dying individuals and suffering populations: applying a population-level bioethics lens to palliative care in humanitarian contexts: before, during and after the COVID-19 pandemic**. *Journal of Medical Ethics* 2020, **46**(8):514-525.

152. Yamamoto V, Bolanos JF, Fiallos J, Strand SE, Morris K, Shahrokhinia S, Cushing TR, Hopp L, Tiwari A, Hariri R *et al*: **COVID-19: Review of a 21st Century Pandemic from Etiology to Neuro-psychiatric Implications**. *Journal of Alzheimer's Disease* 2020, **77**(2):459-504.

153. Yardley S, Rolph M: **Death and dying during the pandemic**. *The BMJ* 2020, **369**:m1472.

154. Zeh RD, Santry HP, Monsour C, Sumski AA, Bridges JFP, Tsung A, Pawlik TM, Cloyd JM: **Impact of visitor restriction rules on the postoperative experience of COVID-19 negative patients undergoing surgery**. *Surgery* 2020, **168**(5):770-776.

155. Zeneli A, Altini M, Bragagni M, Gentili N, Prati S, Golinucci M, Rustignoli M, Montalti S: **Mitigating strategies and nursing response for cancer care management during the COVID-19 pandemic: an Italian experience**. *International nursing review* 2020, **00**:1-11.
